# Synthetic Exploration of Bis(phenolate) Aza-BODIPYs and Heavier Group 13 Chelates

**DOI:** 10.3390/molecules27238256

**Published:** 2022-11-26

**Authors:** Aiden M. Lane, Ny T. C. Luong, Jordan C. Kelly, Martin J. Neal, Jeremiah Jamrom, Aaron J. Bloomfield, Paul A. Lummis, Thomas D. Montgomery, Daniel T. Chase

**Affiliations:** 1 Department of Chemistry and Biochemistry, St. Mary’s College of Maryland, St. Mary’s City, MD 20686, USA; 2 Department of Chemistry and Biochemistry, Duquesne University, Pittsburg, PA 15282, USA

**Keywords:** aza-DIPY, aza-BODIPY, aza-ALDIPY, aza-GADIPY, aza-INDIPY

## Abstract

A series of boron, aluminum, gallium, and indium chelates containing the underexplored bis(phenolate) aza-dipyrromethene (aza-DIPY) core were prepared. These compounds were found to possess near-infrared absorption and emission profiles in the 710 to 770 nm domain and exhibit quantum yield values up to 14%. X-ray diffraction analysis revealed that heavier group 13 bis(phenolate) aza-DIPY chelates possessed octahedral geometries with either THF or pyridine groups occupying the axial positions as opposed to the tetrahedral geometry of the boron chelate.

## 1. Introduction

**Figure 1:**
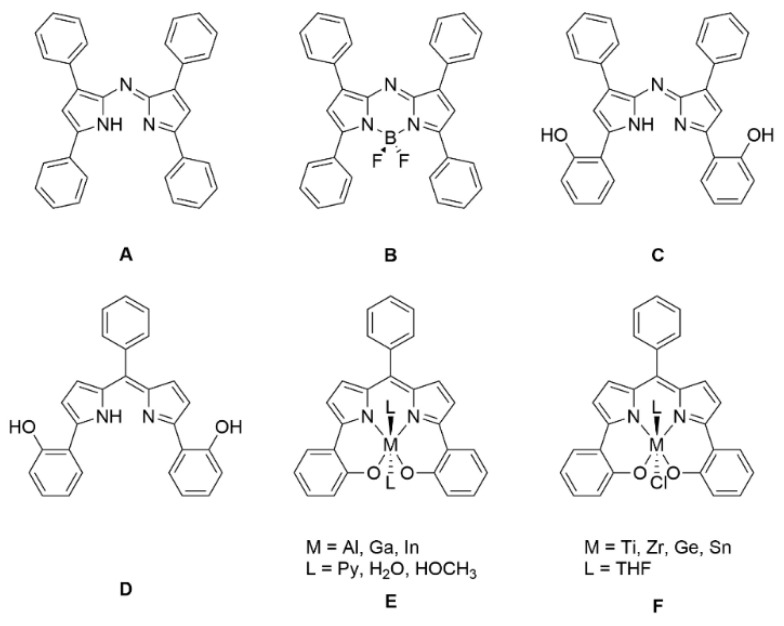
Aza-DIPY (**A**), aza-BODIPY (**B**), bis(phenolate) aza-DIPY (**C**), bis(phenolate) DIPY (**D**), bis(phenolate) DIPY main group chelates (**E**) and bis(phenolate) transition metal chelates (**F**).

Since its resurgence nearly two decades ago, the aza-dipyrromethene (aza-DIPY) core (**A**, Figure 1) has received significant attention due to its intense absorbing and emitting profile in the near-infrared (NIR) region which makes them exciting candidates for application in both the physical and biological sciences [1,2,3,4]. From the perspective of effectively modulating these profiles, boron difluoride chelates of the aza-DIPY core (aza-BODIPYs, (**B**) represent the majority of literature examples as numerous strategies are known to push absorption and emission maxima beyond 800 nm [5,6,7,8,9,10].

In conjunction with our prior work functionalizing aza-BODIPYs through the direct incorporation of nitro groups [11], we became interested with the bis(phenolate) aza-DIPY core structure (**C**), having *ortho*-functionalized phenolic groups present on the proximal rings. Motivation for this core structure stemmed from the previous work done by Burgess and others which demonstrated that bis(phenolate) aza-BODIPYs can achieve absorption and emission maxima upwards of 780 nm [12,13,14,15,16,17,18]. Additionally, very recent work by Sauvé and coworkers detailed that the addition of phenylacetylene groups on the 2- and 6-positions of the bis(phenolate) aza-BODIPY core can further shift absorption and emission maxima past 820 nm [19].

The structurally related bis(phenolate) dipyrromethene core (**D**), whose boron chelates were also originally reported by Burgess and coworkers [20], represents a secondary source of inspiration as numerous elements beyond boron have also been chelated to afford a diverse array of compounds with interesting structural and photophysical properties. For example, Nabeshima and coworkers explored the insertion of heavier Group 13 centers (Al, Ga, In, **E**) and found that such complexes were able to serve as efficient luminescent sensors to both alkaline earth and transition metals [21,22,23]. Further work by their group also considered the insertion of Group 14 and 15 centers which yielded chelates that incorporate both bent and linear oxygen bonds and serve as zwitterionic NIR pH sensors [24,25,26,27]. Significant work on inserting transition metal centers into the bis(phenolate) DIPY core has also been performed. For example, Nozaki and coworkers explored Group 4 and heavier Group 14 (Ti, Zr, Ge, Sn, **F**) DIPY chelates as catalysts for the copolymerization of epoxides with carbon dioxide [28]. Work by Thomas and others have also demonstrated that DIPY chelates containing Mn can serve as catalysts for olefin epoxidations [29,30,31]. Furthermore, numerous reports have indicated that both mid- and late-transition metal DIPY chelates can exhibit both innocent and non-innocent redox properties [32,33,34,35]. Indeed, a recent 2019 report by Kadish and coworkers [36] directly suggested that such behavior with bis(phenolate) DIPYs may very well serve as an analogy for the well-known corrole macrocycle which has received significant attention in its own right over the past three decades. This analogy is also the subject of a very recent review by Paolesse and coworkers [37].

To the best of our knowledge, no chelates outside of boron that contain the bis(phenolate) aza-DIPY core structure are currently known to the literature. Given the likely structural properties that such chelates will share with their DIPY congeners in addition to their expected NIR traits, further research is of clear interest. Here, we disclose the synthesis of a series of aza-BODIPY (**1a-b**), aza-ALDIPY (**2a-b**), aza-GADIPY (**3a-b**), and aza-INDIPY (**4a-b**) complexes. This series of compounds lays the groundwork for a thorough investigation of this comparatively underexplored class of molecules and validates their expected structural and spectral properties. In addition to their ^1^H, ^11^B, ^13^C and HRMS data, we also report the X-ray structures of **1a**, **2a-b**, **3a-b**, and **4a-b** which unambiguously confirm their structural identities. Finally, the computational and photophysical properties of **1a-b**, **2a-b**, **3a-b**, and **4a-b** are discussed and compared.

## 2. Results and Discussion

### 2.1. Synthesis and Structural Characterization

**Scheme 1:**
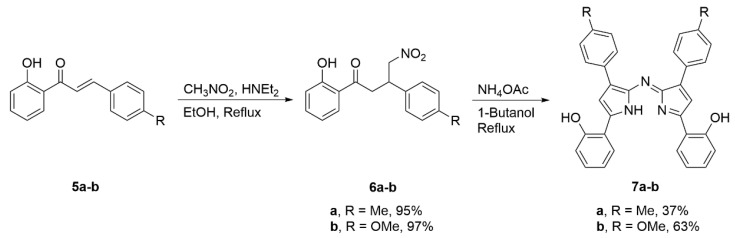
Synthesis of bis(phenolate) aza-DIPY cores **7a-b**.

The synthesis of bis(phenolate) aza-MDIPYs **1a-b**, **2a-b**, **3a-b**, and **4a-b** follows the well-established procedures previously outlined by Burgess [18] and Nabeshima [23] as depicted in Scheme 1. Bis(phenolate) aza-DIPY core structures **7a-b**, containing *para*-functionalized methyl- and methoxyphenyl groups, were specifically chosen as they possess relatively similar electron donating capabilities and will likely exhibit the most bathochromically shifted absorption and emission spectra based on prior work by ourselves and others [11,12,13,14,15,16,17,18]. The first step involves subjecting known chalcones **5a-b** to a Henry reaction with nitromethane to give compounds **6a-b** in excellent yields (>95%). Next, **6a-b** are reacted with an excess of ammonium acetate in refluxing 1-butanol to generate bis(phenolate) aza-DIPYs **7a-b** in moderate yields (37–63%).

**Scheme 2:**
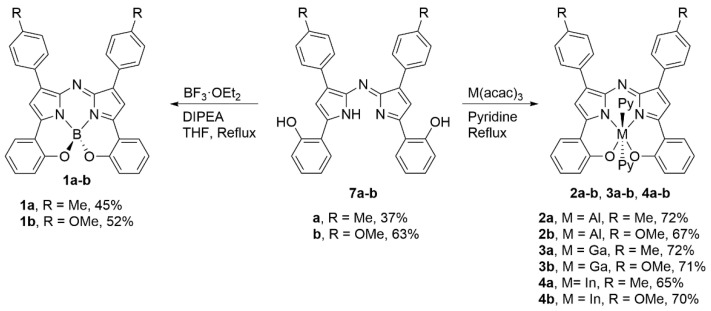
Synthesis of bis(phenolate) aza-BODIPYs **1a-b**, aza-ALDIPYs **2a-b**, aza-GADIPYs **3a-b**, and aza-INDIPYs **4a-b**.

To form bis(phenolate) aza-BODIPYs, the final step involves subjecting **7a-b** to a BF_3_·OEt_2_/DIPEA mixture in refluxing THF, forming **1a-b** in moderate yields (45–52%) as depicted in Scheme 2. To generate heavier Group 13 derivatives, 1.5 equivalents of either Al(acac)_3_, Ga(acac)_3_, or In(acac)_3_ were added to the core in refluxing pyridine to form the corresponding bis(phenolate) aza-ALDIPYs (**2a-b**), aza-GADIPYs (**3a-b**), and aza-INDIPYs (**4a-b**), respectively, in good yields (65–72%).

All reactions were monitored through observation of a characteristic upfield shift of the pyrrolic ^1^H signal correlating to the 2- and 6-positions of the bis(phenolate) aza-DIPY core. Additionally, boron chelations were followed through the expected appearance of a ^11^B singlet between −3.5 and −2.5 ppm. For aluminum, gallium, and indium chelations, we observed the appearance of pyridine signals in an approximate 2:1 ratio with respect to the bis(phenolate) aza-DIPY core, implying the inclusion of two axially bound pyridine units to the metal center, completing their expected octahedral geometries.

### 2.2. Crystallography

**Figure 2:**
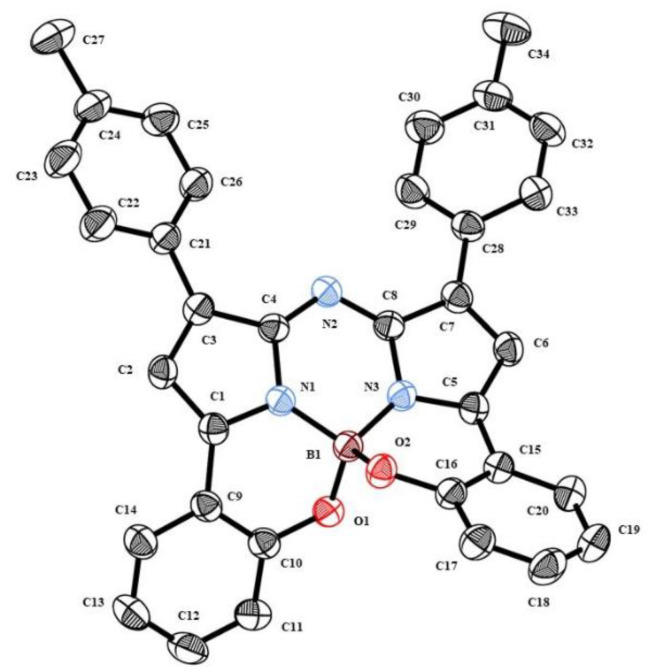
Molecular structure of **1a**. Ellipsoids are drawn at the 40% probability level. Hydrogen atoms have been removed for clarity.

Single crystals of bis(phenolate) aza-BODIPY **1a** were obtained through the slow vapor diffusion of a THF solution of **1a** with n-pentane. Analysis of the diffraction data unambiguously confirms the structural identity of **1a** and its expected tetrahedral boron center (Figure 2). To accommodate this coordination geometry, both phenolic rings exhibit significant flexing as demonstrated by torsion angles of −22.23(9)° and −24.41(4)° along C2–C1–C9–C14 and C6–C5–C15–C20, respectively. 

**Figure 3:**
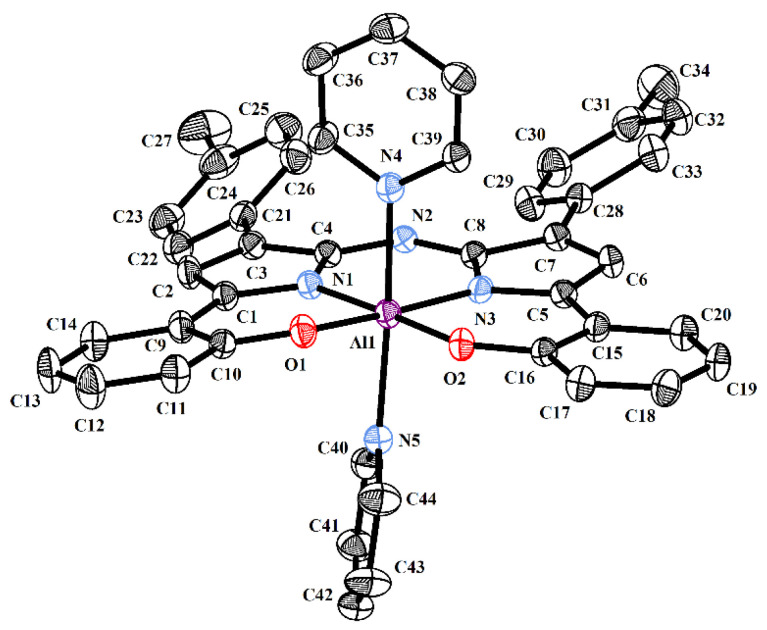
Molecular structure of **2a**. Ellipsoids are drawn at the 40% probability level. Hydrogen atoms have been removed for clarity.

**Figure 4:**
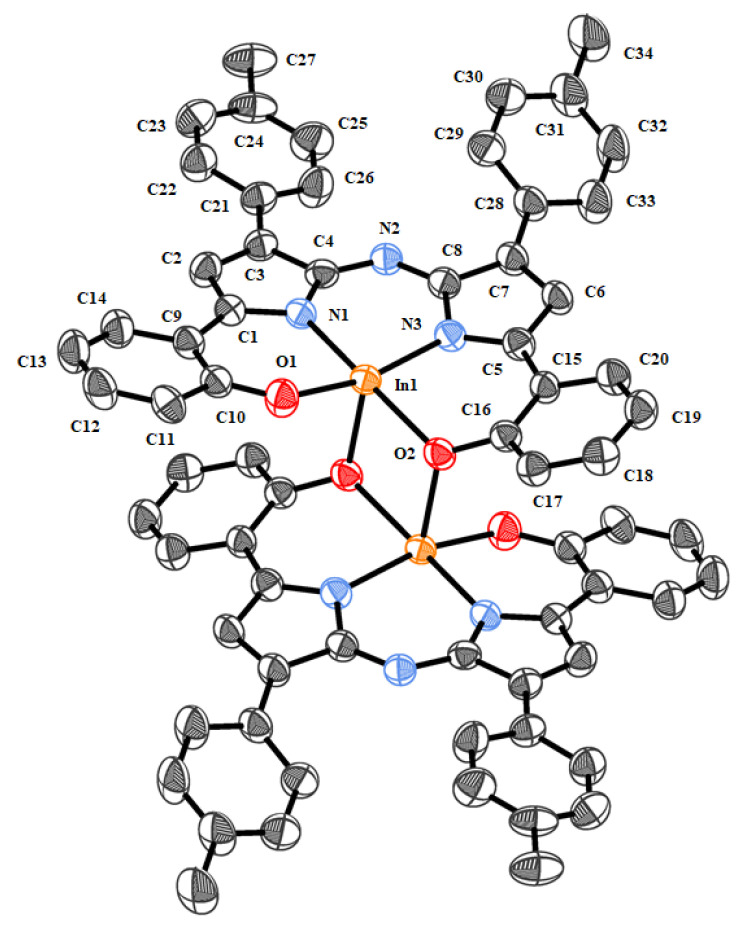
Molecular structure of **4a’**. Ellipsoids are drawn at the 40% probability level. Axial THF groups and hydrogen atoms have been removed for clarity.

Single crystals of bis(phenolate) aza-ALDIPY **2a** were obtained through layering n-hexane in a 1:1 pyridine:toluene solution followed by slow evaporation. As seen in Figure 3, two pyridine groups are bound axially to the Al center, giving a nearly linear angle of 176.55(7)° along the N4–Al1–N5 axis. The Al center also fits within the plane of the bis(phenolate) aza-DIPY core and we observe the phenolic rings puckering slightly inwards, resulting in an interplanar angle of 3.31(8)°. These structural trends are similarly found with the diffraction data of **2b**, **3a-b**, and **4a-b** (see the Supporting Information for full details). Interestingly, when THF was used as a coordinating solvent instead of pyridine, single crystals of bis(phenolate) aza-INDIPY **4a’** were obtained through layering n-decane over a saturated THF solution. As seen in Figure 4, **4a’** incorporates two identical octahedral aza-INDIPY cores that are dimerized together forming a central four-membered ring that contains an In_2_O_2_ moiety (In1-O2: 2.141 Å; In1-O2’: 2.224 Å). The axially bound THF groups (omitted for clarity, see Supporting Information) are also moderately bent out of linearity as evidenced from the 163.81(12)° angle along the O3–In1–O2’ axis. There is also significant twisting (17.44(3)°) between the axially bound phenolic group and the aza-DIPY core to accommodate dimerization which also explains the discrete In-O bond lengths.

### 2.3. Computational Measurements

**Figure 5:**
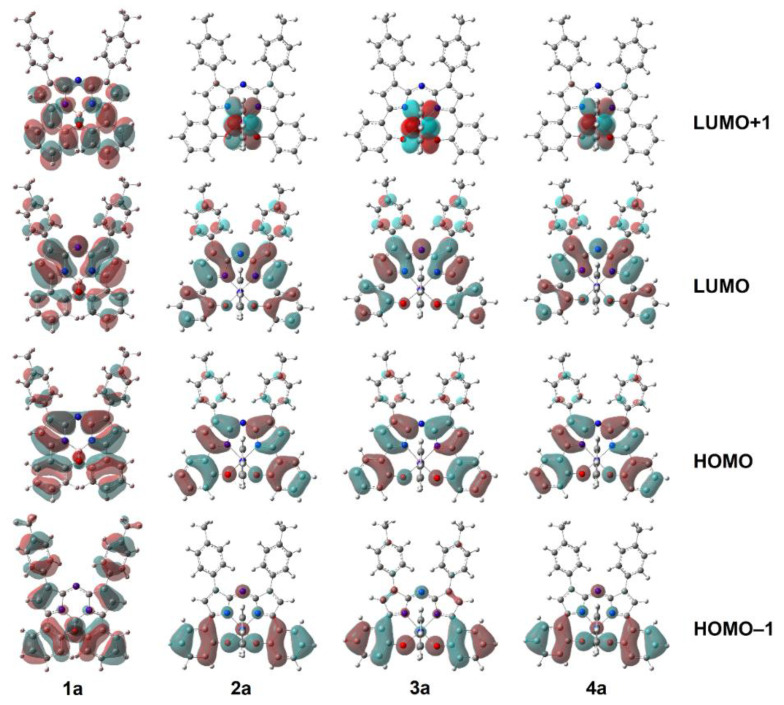
HOMO-1, HOMO, LUMO, and LUMO+1 plots of **1a**, **2a**, **3a**, and **4a**. Calculations were performed at the B3LYP/jul-cc-pVDZ level of theory for **1a**, **2a**, and **3a** and the B3LYP/LanL2DZ level of theory for **4a** with MeOH as the implicit solvent for all compounds.

To better understand the photophysical properties of **1a-b**, **2a-b**, **3a-b**¸and **4a-b**, density functional theory (DFT) calculations were performed [38,39]. All data are given in Table 1 with the frontier molecular orbitals of **1a**, **2a, 3a**, and **4a** displayed in Figure 5 (see the Supporting Information for full details). Using the experimentally determined crystal structure of **2a** as a reference, the computed ground state geometries were found to be in excellent agreement using the B3LYP functional with Dunning’s jul-cc-pVDZ basis set [40]. The jul-cc-pVDZ basis set was selected due to its good performance in a variety of systems [41] and in our prior studies, we found it gave the best balance of accuracy and computational cost for functionalized DIPYs [11]. With the optimized structures in hand, TD-DFT calculations using B3LYP/jul-cc-pVDZ for **1a-b**, **2a-b**, and **3a-b** with MeOH as the implicit solvent [42,43] were performed; for **4a-b**, B3LYP/LanL2DZ was used for both geometry optimization and TD-DFT calculations to accommodate the In nuclei [44,45]. Overall, our computations generated theoretical λ_abs_ values that closely matched their experimental values with deviations below 5%; the sole exception to this was bis(phenolate) aza-BODIPY **1a** which exhibited a deviation of 9.1%.

As seen in Figure 5, both methyl and methoxy-functionalized bis(phenolate) aza-MDIPYs possess little orbital density on the distal arenes which suggests that such functionalization bears little influence. Instead, the majority of HOMO and LUMO density resides on the aza-DIPY core, resulting in the expected π-π* transitions. Furthermore, little to no orbital density was found on the metal centers which agrees with numerous aza-DIPY studies performed previously [12,13,14,15,16,17,18,46,47]. Perhaps the greatest difference between the frontier molecular orbital plots of these compounds involves the LUMO+1 orbital as **1a-b** has orbital density spread throughout the proximal rings and aza-DIPY core while **2a-b**, **3a-b**, and **4a-b** have orbital density exclusively on the axially bound pyridines. These structures were also visualized in the DIPY plane, with the images included in the SI.

### 2.4. Photophysical Properties

#### 2.4.1. Electronic Absorption Spectra

**Figure 6:**
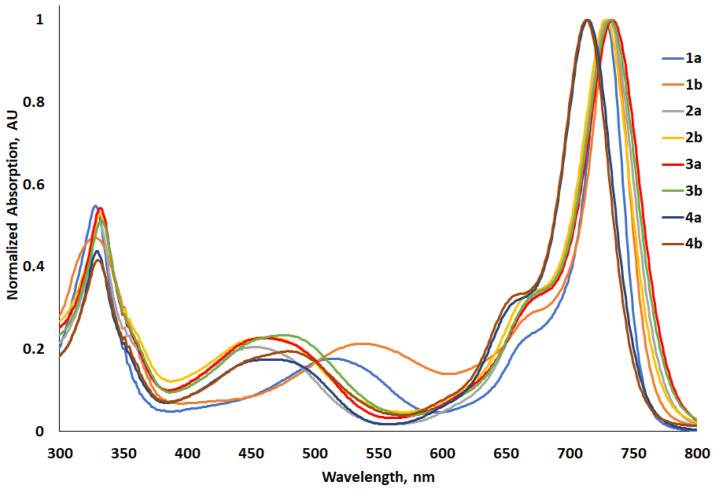
Absorption spectra of **1a-b**, **2a-b**, **3a-b**, and **4a-b** in DMSO. All spectra are recorded at a concentration of 10 μM.

The absorption spectra for **1a-b**, **2a-b**, **3a-b** and **4a-b** in DMSO are shown in Figure 6 and listed in Table 1. As expected with most aza-BODIPYs, all compounds exhibited intense absorption maxima between 715–735 nm with extinction coefficients between 30,000 and 52,000 M^−1^ cm^−1^. Also present are smaller absorption maxima located at the 650 nm, 450 nm, and 325 nm ranges. Interestingly, **1a-b** exhibit bathochromically shifted absorptions in the 525 nm range as compared to **2a-b**, **3a-b**, and **4a-b** which give them a purple hue compared to the green color that the remaining complexes possess. As predicted by the frontier molecular orbital plots mentioned above, *para*-functionalization on the distal positions of the bis(phenolate) aza-DIPY core had little effect on the absorption maxima which was similarly observed by Burgess and coworkers regarding aza-BODIPYs [18]. The general trend was found to be B > Ga > Al > In with bis(phenolate) aza-BODIPYs **1a-b** exhibiting the most bathochromically shifted absorption spectra which fits well with prior observations found by Nabeshima and co-workers [23].

#### 2.4.2. Electronic Emission Spectra

**Figure 7:**
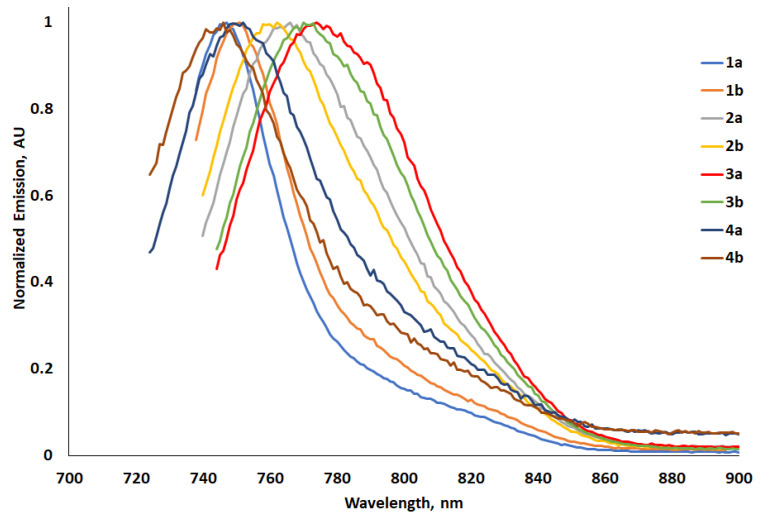
Emission spectra of **1a-b**, **2a-b**, **3a-b**, and **4a-b** in DMSO. All spectra are recorded at a concentration of 10 μM. λ_abs_ was used as the excitation wavelength for λ_em_.

The emission spectra for **1a-b**, **2a-b**, **3a-b** and **4a-b** in DMSO are shown in Figure 7 and listed in Table 1. Unlike the absorption data, the general emission trend stands at Ga > Al > B > In with bis(phenolate) aza-GADIPYs **2a-b** exhibiting the most bathochromically shifted spectra. As found with the observations originally made by both Nabeshima and Burgess [18,23], methyl functionalized derivatives exhibited bathochromically shifted emission maxima when compared to their methoxy functionalized derivatives. With respect to Stokes shifts, the general trend stands at Ga > In > Al > B; this observation is reasoned to be due to the difference between the octahedral geometry of **2a-b**, **3a-b**, and **4a-b** compared to the tetrahedral geometry of **1a-b**. Quantum yields for **1a-b**, **2a-b**, **3a-b**, and **4a-b** were also measured in MeOH using an internal integrating sphere and their values are given in Table 1. It was found that quantum yields increased from **1a-b** to **2a-b** but then decreased as a function of increasing metal size.

## 3. Materials and Methods

### 3.1. General Considerations

Unless otherwise noted, solvents and reagents were purchased from either Thermo Fisher or Sigma Aldrich and used as received. Flash chromatography was performed using P60 silica purchased from SiliCycle. THF was distilled from sodium/benzophenone under a nitrogen atmosphere. Bis(phenolate) aza-BODIPY **7b** and their precursors, **5b** and **6b**, were synthesized by their previously reported methods [18,23].

NMR spectra were recorded using a JEOL JMN-ECSZ400R 400 MHz (^1^H: 399.78 MHz; ^13^C: 100.52 MHz) spectrometer at room temperature. Chemical shifts (δ) are expressed in ppm relative to the residual chloroform (^1^H: 7.27 ppm; ^13^C: 77.2 ppm) or DMSO-d^6^ (^1^H: 2.50 ppm; ^13^C: 39.5 ppm) in solution. Coupling constants are expressed in hertz. UV-Visible spectra were recorded on a Cary 100 UV-Vis spectrometer. Fluorescence spectra were recorded on a PTI HORIBA QM-40 spectrofluorometer and quantum yields were obtained using a KSPHERE petite integrating sphere. Single crystal XRD measurements were collected from a Bruker Smart Apex II CCD diffractometer using graphite-monochromated Mo-K (λ_α_ = 0.71073 Å) radiation. Molecular crystal structure images were processed through CrystalMaker. HRMS-ESI measurements were taken on an Agilent 6530 high resolution quadrupole time of flight spectrometer (QTOF) utilizing an Agilent 1100 liquid chromatography system with model G1212A binary pump and G1367 autosampler. Samples were introduced via direct injection rather than isolating species with column chromatography to monitor changing levels of starting material as well as any potential side reactions which were ionized using a Dual Agilent Jet Stream G1958-66268 electrospray ionization source. Immediately prior to sample introduction, the QTOF was calibrated in the 50–1700 mass range for the 4 GHz high resolution mode. A mobile phase of HPLC acetone was utilized to reduce instrument noise related to the solvent and to ensure the analyte would stay in solution until ionized. Acetone was utilized as a needle wash to reduce contamination of samples. Samples were analyzed in the positive mode with a dragging gas temperature of 300 °C, a drying gas flow rate of 8 L/min, a nebulizer pressure of 35 psi, a sheath gas temperature of 350 °C, a sheath gas flow rate of 11 L/min, and a capillary voltage of 3500 V. 

### 3.2. Synthesis and Characterization

#### 3.2.1. 1,4-Product **6a**

Chalcone **5a** (6.00 g, 25.2 mmol) was dissolved in EtOH (150 mL) where diethylamine (13.0 mL, 126 mmol) and nitromethane (6.80 mL, 126 mmol) were added and the reaction mixture was heated to reflux and allowed to stir overnight. Upon cooling, the resulting mixture was evaporated to dryness and 1,4-product **6a** (7.17 g, 95%) was obtained as a tan brown oil. ^1^H NMR (CDCl_3_): δ 11.99 (s, 1H), 7.73 (dd, *J* = 1.6, 7.2 Hz, 1H), 7.48 (dt, *J* = 1.6, 7.6 Hz, 1H), 7.17 (m, 4H), 6.98 (d, *J* = 9.2 Hz), 6.91 (t, *J* = 8.4 Hz), 4.74 (m, 1H), 4.68 (m, 1H), 4.20 (quintet, 1H), 3.48 (m, 2H), 2.33 (s, 3H). ^13^C NMR (CDCl_3_): δ 202.9, 162.7, 138.0, 137.0, 135.7, 130.0, 129.7, 127.4, 119.3, 119.2, 118.9, 79.9, 41.3, 39.0, 21.3. HRMS (ESI) for C_17_H_17_NO_4_ [M + Na]^+^: calcd 322.1050, found 322.1090. 

#### 3.2.2. 1,4-Product **6b**

Chalcone **5b** (5.00 g, 19.7 mmol) was dissolved in EtOH (150 mL) where diethylamine (10.1 mL, 98.3 mmol) and nitromethane (5.26 mL, 98.3 mmol) were added and the reaction mixture was heated to reflux and allowed to stir overnight. Upon cooling, the resulting mixture was evaporated to dryness and 1,4-product **6b** (6.03 g, 97%) was obtained as a tan brown oil. The compound’s ^1^H NMR spectrum matched the known spectrum [18].

#### 3.2.3. Bis(phenolate) Aza-DIPY **7a**

1,4–Product **6a** (5.00 g, 16.7 mmol) and ammonium acetate (40.0 g, 519 mmol) were dissolved in 1-butanol (75 mL). The reaction mixture was heated to reflux and allowed to stir overnight. Upon cooling to room temperature, the resulting precipitate was collected over filter paper, washed with H_2_O (200 mL) and EtOH (200 mL), and the solid was allowed to dry in the oven at 85 °C to obtain **7a** (1.57 g, 37%) as a brown iridescent solid. ^1^H NMR (DMSO-d_6_): δ 8.09 (d, *J* = 7.6 Hz, 2H), 7.97 (d, *J* = 8.0 Hz, 4H), 7.70 (s, 2H), 7.38 (dt, *J* = 1.6, 7.6 Hz, 2H), 7.27 (d, *J* = 8.0 Hz, 4H), 7.12 (d, *J* = 8.0 Hz, 2H), 7.03 (t, *J* = 7.6 Hz, 2H), 2.40 (s, 6H). Due to insolubility, a ^13^C NMR spectrum could not be obtained. HRMS (ESI) for C_34_H_27_N_3_O_2_ [M + H]^+^: calcd 510.2176, found 510.2169. 

#### 3.2.4. Bis(phenolate) Aza-DIPY **7b**

1,4–Product **6b** (5.00 g, 15.9 mmol) and ammonium acetate (40.0 g, 519 mmol) were dissolved in 1-butanol (75 mL). The reaction mixture was heated to reflux and allowed to stir overnight. Upon cooling to room temperature, the resulting precipitate was collected over filter paper, washed with H_2_O (200 mL) and EtOH (200 mL), and the solid was allowed to dry in the oven at 85 °C to obtain **7b** (1.60 g, 37%) as a brown iridescent solid. The compound’s ^1^H NMR spectrum matched the known spectrum [18].

#### 3.2.5. Bis(phenolate) Aza-BODIPY **1a**

To a degassed (30 min) solution of THF, bis(phenolate) aza-DIPY **7a** (0.050 g, 0.098 mmol) was added along with *N*,*N*-diisopropylethylamine (0.26 mL, 1.5 mmol). BF_3_·OEt_2_ (0.24 mL, 2.0 mmol) was added carefully and the mixture was allowed to stir at reflux overnight. After completion by NMR, the mixture was evaporated to dryness and subsequently chromatographed on silica (CH_2_Cl_2_) to afford the appropriate bis(phenolate) aza-BODIPY **1a** (0.023 g, 45%) as an iridescent green powder. ^1^H NMR (DMSO-d^6^): δ 8.14 (d, *J* = 8.0 Hz, 4H), 8.09 (d, *J* = 8.0 Hz, 2H), 7.82 (s, 2H), 7.48 (t, *J* = 8.0 Hz, 2H), 7.39 (d, *J* = 8.0 Hz, 4H), 7.20 (t, *J* = 8.4 Hz, 2H), 6.99 (d, *J* = 8.0 Hz, 2H), 2.41 (s, 6H). ^13^C NMR (DMSO–d^6^): δ 155.4, 149.5, 144.0, 141.2, 139.7, 133.9, 129.7, 129.1, 128.7, 127.3, 121.3, 119.7, 118.4, 113.9, 21.1. ^11^B NMR (DMSO–d^6^): δ -3.36. HRMS (ESI) for C_34_H_24_BN_3_O_2_ [M + EtOH + NH_4_]^+^: calcd 581.2178, found 581.2717.

#### 3.2.6. Bis(phenolate) Aza-BODIPY **1b**

To a degassed (30 min) solution of THF, bis(phenolate) aza-DIPY **7b** (0.050 g, 0.092 mmol) was added along with *N*,*N*-diisopropylethylamine (0.24 mL, 1.4 mmol). BF_3_·OEt_2_ (0.23 mL, 1.8 mmol) was added carefully and the mixture was allowed to stir at reflux overnight. After completion by NMR, the mixture was evaporated to dryness and subsequently chromatographed on silica (CH_2_Cl_2_) to afford the appropriate bis(phenolate) aza-BODIPY **1b** (0.023 g, 52%) as an iridescent green powder. The compound’s ^1^H NMR spectrum matched the known spectrum [18].

#### 3.2.7. Bis(phenolate) Aza-ALDIPY **2a**

To a solution of pyridine (15 mL), bis(phenolate) aza-DIPY **7a** (0.150 g, 0.294 mmol) and Al(acac)_3_ (0.146 g, 0.441 mmol) were added and the mixture was heated to reflux overnight. Upon cooling to room temperature, hexanes (30 mL) was added to the mixture and cooled to 0 °C. Once cold, the resulting precipitate was collected over filter paper, washed with additional hexanes (30 mL) and evaporated from dryness to afford **2a** (0.212 g, 72%) as a dark green solid. ^1^H NMR (DMSO-d_6_): δ 8.58 (m, 4H), 7.97 (d, *J* = 8.0 Hz), 7.78 (m, 2H), 7.70 (dd, *J* = 2.0, 8.2 Hz, 2H), 7.38 (m, 4H), 7.37 (s, 2H), 7.23 (d, *J* = 8.0 Hz), 7.11 (dt, *J* = 0.8, 7.8 Hz, 2H), 6.69 (dd, *J* = 0.8, 7.8 Hz, 2H), 2.38 (s, 6H). ^13^C NMR (DMSO-d_6_): δ 163.8, 156.5, 149.6, 147.0, 140.3, 136.8, 136.1, 131.5, 131.3, 128.6, 128.0, 123.9, 121.0, 119.1, 115.2, 114.2, 20.9. HRMS (ESI) for C_34_H_24_AlN_3_O_2_ [M + 3H]^+^: calcd 536.1913, found 536.1911. 

#### 3.2.8. Bis(phenolate) Aza-ALDIPY **2b**

To a solution of pyridine (15 mL), bis(phenolate) aza-DIPY **7b** (0.150 g, 0.277 mmol) and Al(acac)_3_ (0.124 g, 0.415 mmol) were added and the mixture was heated to reflux overnight. Upon cooling to room temperature, hexanes (30 mL) was added to the mixture and cooled to 0 °C. Once cold, the resulting precipitate was collected over filter paper, washed with additional hexanes (30 mL) and evaporated from dryness to afford **1b** (0.134 g, 67%) as a dark green solid. ^1^H NMR (DMSO-d_6_): δ 8.57 (m, 4H), 8.00 (d, *J* = 8.8 Hz, 4H), 7.78 (m, 2H), 7.69 (dd, *J* = 1.2, 7.8Hz, 2H), 7.39 (m, 4H), 7.32 (s, 2H), 7.10 (dt, *J* = 1.2, 7.8 Hz, 2H), 6.99 (d, *J* = 8.8 Hz, 4H), 6.68 (d, *J* = 8.0 Hz, 2H), 6.56 (t, *J* = 8.0 Hz, 2H), 3.83 (s, 6H). ^13^C NMR (DMSO-d_6_): δ 163.8, 158.9, 156.5, 149.7, 149.9, 140.2, 139.2, 131.5, 130.1, 128.1, 126.8, 123.9, 121.1, 119.2, 115.2, 113.6, 55.2. HRMS (ESI) for C_34_H_24_AlN_3_O_4_ [M + 4H]^+^: calcd 569.1890, found 569.1899.

#### 3.2.9. Bis(phenolate) Aza-GADIPY **3a**

To a solution of pyridine (15 mL), bis(phenolate) aza-DIPY **7a** (0.150 g, 0.294 mmol) and Ga(acac)_3_ (0.162 g, 0.441 mmol) were added and the mixture was heated to reflux overnight. Upon cooling to room temperature, hexanes (30 mL) was added to the mixture and cooled to 0 °C. Once cold, the resulting precipitate was collected over filter paper, washed with additional hexanes (30 mL) and evaporated from dryness to afford **3a** (0.156 g, 72%) as a dark green solid. ^1^H NMR (DMSO-d_6_): δ 8.57 (m, 2H), 7.97 (d, *J* = 8.0 Hz, 4H), 7.79 (m, 2H), 7.74 (dt, *J* = 2.0, 8.2 Hz, 2H), 7.46 (s, 2H), 7.39 (m, 4H), 7.24 (d, *J* = 8.0 Hz, 4H), 7.12 (dt, *J* = 2.0, 7.2 Hz, 2H), 6.73 (dd, *J* = 1.2, 8.2 Hz, 2H), 6.59 (dt, 1.2, 7.4 Hz, 2H), 2.39 (s, 6H). ^13^C NMR (DMSO-d_6_): δ 165.8, 157.0, 149.6, 146.3, 140.4, 137.1, 136.2, 131.6, 131.1, 128.8, 128.7, 128.6, 123.9, 121.9, 117.7, 115.4, 114.4, 21.0. HRMS (ESI) for C_34_H_24_GaN_3_O_2_ [M + 3H]^+^: calcd 578.1354, found 578.1347. 

#### 3.2.10. Bis(phenolate) Aza-GADIPY **3b**

To a solution of pyridine (15 mL), bis(phenolate) aza-DIPY **7b** (0.150 g, 0.277 mmol) and Ga(acac)_3_ (0.152 g, 0.415 mmol) were added and the mixture was heated to reflux overnight. Upon cooling to room temperature, hexanes (30 mL) was added to the mixture and cooled to 0 °C. Once cold, the resulting precipitate was collected over filter paper, washed with additional hexanes (30 mL) and evaporated from dryness to afford **3b** (0.151 g, 71%) as a dark green solid. ^1^H NMR (DMSO-d_6_): δ 8.57 (m, 4H), 8.00 (d, *J* = 9.2 Hz, 4H), 7.79 (m, 2H), 7.73 (dt, *J* = 1.2, 8.2 Hz, 2H), 7.39 (m, 6H), 7.11 (dt, *J* = 1.6, 7.8 Hz, 2H), 7.00 (d, *J* = 9.2 Hz, 4H), 6.73 (d, *J* = 8.0 Hz, 2H), 6.58 (t, *J* = 8.0 Hz, 2H), 3.84 (s, 6H). ^13^C NMR (DMSO-d_6_): δ 165.8, 159.1, 157.0, 149.6, 146.2, 140.3, 136.3, 136.2, 131.6, 130.2, 128.6, 126.5, 124.0, 121.9, 117.8, 115.4, 113.7, 113.6, 55.2. HRMS (ESI) for C_34_H_24_GaN_3_O_4_ [M + 3H]^+^: calcd 610.1252, found 610.1278. 

#### 3.2.11. Bis(phenolate) Aza-INDIPY **4a**

To a solution of pyridine (15 mL), bis(phenolate) aza-DIPY **7a** (0.150 g, 0.294 mmol) and In(acac)_3_ (0.182 g, 0.441 mmol) were added and the mixture was heated to reflux overnight. Upon cooling to room temperature, hexanes (30 mL) was added to the mixture and cooled to 0 °C. Once cold, the resulting precipitate was collected over filter paper, washed with additional hexanes (30 mL) and evaporated from dryness to afford **4a** (0.149 g, 65%) as a dark green solid. ^1^H NMR (DMSO–d_6_): δ 8.57 (m, 4H), 7.94 (d, *J* = 8.4 Hz, 4H), 7.84 (dd, *J* = 1.6, 8.0 Hz, 2H), 7.79 (m, 2H), 7.59 (s, 2H), 7.38 (m, 4H), 7.23 (d, *J* = 8.0 Hz, 4H), 7.14 (dt, *J* = 1.6, 7.6 Hz, 2H), 6.78 (d, *J* = 8.0 Hz, 2H), 6.61 (dt, *J* = 0.8, 7.4 Hz, 2H), 2.39 (s, 6H). ^13^C NMR (DMSO–d_6_): δ 167.2, 158.7, 149.6, 146.0, 141.6, 137.1, 136.2, 131.3, 131.2, 129.9, 129.0, 128.7, 123.9, 123.2, 118.4, 116.0, 115.4, 21.0. HRMS (ESI) for C_34_H_24_InN_3_O_2_ [M + 3H]^+^: calcd 624.1137, found 624.1128.

#### 3.2.12. Bis(phenolate) Aza-INDIPY **4b**

To a solution of pyridine (15 mL), bis(phenolate) aza-DIPY **7b** (0.150 g, 0.277 mmol) and In(acac)_3_ (0.171 g, 0.415 mmol) were added and the mixture was heated to reflux overnight. Upon cooling to room temperature, hexanes (30 mL) was added to the mixture and cooled to 0 °C. Once cold, the resulting precipitate was collected over filter paper, washed with additional hexanes (30 mL) and evaporated from dryness to afford **4b** (0.157 g, 70%) as a dark green solid. ^1^H NMR (DMSO-d_6_): δ 8.57 (m, 4H), 7.98 (d, *J* = 8.0 Hz, 4H), 7.83 (dd, *J* = 1.6, 8.0 Hz, 2H), 7.79 (m, 2H), 7.53 (s, 2H), 7.38 (m, 4H), 7.13 (dt, *J* = 1.6, 7.6 Hz, 2H), 6.99 (d, *J* = 8.8 Hz, 4H), 6.78 (dd, *J* = 0.8, 8.0 Hz, 2H), 6.61 (dt, *J* = 0.8, 7.6 Hz, 2H), 3.84 (s, 6H). ^13^C NMR (DMSO-d_6_): δ 167.2, 159.1, 158.6, 149.6, 145.9, 141.4, 136.2, 131.2, 130.5, 129.8, 126.7, 123.9, 123.2, 118.5, 115.4, 115.2, 113.6, 55.2. HRMS (ESI) for C_34_H_24_InN_3_O_4_ [M + 2H]^+^: calcd 655.0957, found 655.0953.

## 4. Conclusions

The synthesis and characterization of stable NIR absorbing and emitting bis(phenolate) aza-BODIPYs **1a-b**, aza-ALDIPYs **2a-b**, aza-GADIPYs, **3a-b**, and aza-INDIPYs **4a-b** are reported. Single crystal X-ray diffraction data was able to elucidate that heavier group 13 analogues are able to exist as octahedral complexes that can support either THF or pyridine groups as coordinating ligands. Combined, these experimental results lead to the conclusion that the bis(phenolate) aza-DIPY core is a viable choice for chelating elements beyond boron. Future work will explore the structural consequences of chelating the bis(phenolate) aza-DIPY core to both main group and transition metals. 

## Figures and Tables

**Table 1:** Photophyiscal and computational data for bis(phenolate) aza-DIPYs **1a-b**, **2a-b**, **3a-b**, and **4a-b**.

	Photophysical ^a^							Computational ^d^			
Compound	λ_abs_	log ε	E_gap_^b^	λ_em_^c^	SS ^c^ (nm)	SS ^c^ (cm^−1^)	Φ^c^	E_HOMO_	E_LUMO_	E_Gap_	λ_Gap_
**1a**	728	4.50	1.71	747	19	350	13.7	−5.56	−3.66	1.90	652
**1b**	732	4.47	1.70	751	19	345	8.8	−5.21	−3.43	1.78	695
**2a**	731	4.48	1.70	766	35	625	7.5	−5.00	−3.20	1.80	689
**2b**	728	4.56	1.71	762	34	613	7.0	−4.97	−3.17	1.80	689
**3a**	734	4.58	1.69	774	40	704	6.1	−5.01	−3.21	1.80	688
**3b**	732	4.71	1.70	770	38	674	8.4	−4.98	−3.17	1.81	688
**4a**	714	4.67	1.74	752	38	708	0.3	−5.01	−3.14	1.87	661
**4b**	713	4.45	1.74	746	37	620	0.7	−4.98	3.11	1.87	665

^a^ Spectra were obtained in MeOH at 10 μM. Wavelengths are in nm. ^b^ Optical HOMO/LUMO gap was determined at λ_abs_. ^c^ λ_abs_ was used as the excitation wavelength for λ_em_. Stokes shifts (SS) are measured in both nm and cm^−1^. Quantum yields are measured using an internal integrating sphere in ambient air at room temperature and are reported in %. ^d^ Calculations were performed using B3LYP/jul-cc-pVDZ for **1a-b**, **2a-b**, and **3a-b** with MeOH as implicit solvent; B3LYP/LanL2DZ was used for **4a-b** with MeOH as implicit solvent.

## Supplementary Materials

The following supporting information can be downloaded at: https://www.mdpi.com/article/10.3390/molecules27238256/s1, Figures S1–S8: molecular orbital plots of **1a-b**, **2a-b**, **3a-b**, and **4a-b**, Figures S9–S16: single crystal X-ray diffraction analyses of **1a**, **2a-b**, **3a-b**, **4a-b**, and **4a’**, Tables S1 and S2: UV-Vis calculations with TD-DFT computations, Tables S3–S6: crystallographic structure data for **1a**, **2a-b**, **3a-b**, **4a-b**, and **4a’**, cartesian coordinates of **1a**, **2a-b**, **3a-b**, and **4a-b**, NMR spectra of **1a**, **2a-b**, **3a-b**, **4a-b**, and **4a’**. Data structures for **1a**, **2a-b**, **3a-b**, **4a-b**, and **4a’** were deposited in the Cambridge Crystallographic Data Center (CCDC) under the deposit numbers 2168300-2168305, 21903914, and 2215010 and can be obtained free of charge via http://www.ccdc.cam.ac.uk.

## Sample Availability

Samples of all compounds are available from the authors at request.
