# Supplementary material for: Synthetic Exploration of Bis(phenolate) Aza-BODIPYs and Heavier Group 13 Chelates
Source: Molecules. 2022 Nov 26;27(23):8256. doi: 10.3390/molecules27238256 (PMC9737893; doi:10.3390/molecules27238256)
Supplement: Supplementary file 1 [file molecules-27-08256-s001.zip › molecules-2027412-supplementary.pdf]

Supplementary Information

Synthetic Exploration of Bis(phenolate) aza-BODIPYs and Heavier Group 13 Chelates

Aiden M. Lane,† Ny T. C. Luong,† Jordan C. Kelly,‡ Martin J. Neal,‡ Jeremiah Jamrom,‡ Aaron J. Bloomfield,‡ Paul A. Lummis,‡ Thomas D. Montgomery,*‡ and Daniel T. Chase*†,

†Department of Chemistry and Biochemistry, St. Mary's College of Maryland, St. Mary's City, Maryland 20686

‡Department of Chemistry and Biochemistry, Duquesne University, Pittsburgh, Pennsylvania 15282

Email: dtchase@smcm.edu; montgomeryt1@duq.edu

Table of Contents

Cartesian Coordinates of Stationary Points for **1a-b**, **2a-b**, **3a-b**, and **4a-b** S2

HOMO/LUMO Energies and Orbital Maps for **1a-b**, **2a-b**, **3a-b**, and **4a-b** S20

Single crystal X-ray diffraction structures for **1a**, **2a-b**, **3a-b**, **4a-b**, and **4a'** S26

Crystallographic data and refinement parameters for **1a**, **2a-b**, **3a-b**, **4a-b**, and **4a'** S37

Crystallographic bond and dihedral angles for **1a**, **2a-b**, **3a-b**, **4a-b**, and **4a'** S41

References S43

NMR Spectra S44

Cartesian Coordinates

1a

Imaginary Freq	0	
Temperature	298.150	Kelvin
Pressure	1.00000	atm
Frequencies scaled by	1.0000	
Electronic Energy (EE)	-1649.987899	Hartree
Zero-point Energy Correction	0.495678	Hartree
Thermal Correction to Energy	0.526563	Hartree
Thermal Correction to Enthalpy	0.527508	Hartree
Thermal Correction to Free Energy	0.431019	Hartree
EE + Zero-point Energy	-1649.492221	Hartree
EE + Thermal Energy Correction	-1649.461335	Hartree
EE + Thermal Enthalpy Correction	-1649.460391	Hartree
EE + Thermal Free Energy Correction	-1649.556880	Hartree
E (Thermal)	330.424	kcal/mol
Heat Capacity (Cv)	124.980	cal/mol-kelvin
Entropy (S)	203.077	cal/mol-kelvin

B	-1.999020	-0.000000	-0.000007
C	6.345344	4.211769	-0.888230
O	-2.884194	-0.386858	-1.127632
N	1.005383	0.000007	-0.000017
N	-1.066528	1.171658	-0.232528
N	-1.066520	-1.171653	0.232506
O	-2.884185	0.386852	1.127627
C	4.913605	3.752410	-0.760561
C	3.181050	-2.062756	1.083446
H	2.893187	-1.081285	1.456653
C	6.345344	-4.211786	0.888276
C	-2.794884	-2.703337	-0.371722
C	-1.454915	-2.463405	0.115456
C	0.324567	-1.111237	0.268790
C	4.504009	-2.489904	1.215143
H	5.232178	-1.827863	1.688475
C	-3.418284	-3.968123	-0.345718
H	-2.926753	-4.789010	0.178473
C	0.811928	-2.471456	0.370739
C	-0.313432	-3.296866	0.266840
H	-0.323951	-4.381535	0.252010
C	0.324559	1.111247	-0.268819
C	2.210191	-2.892499	0.489965
C	4.913615	-3.752402	0.760567
C	3.943295	4.580905	-0.167782
H	4.231109	5.568200	0.200048
C	2.210178	2.892511	-0.489981

C	-3.437918	-1.620095	-1.051629
C	2.621401	4.163468	-0.034726
H	1.900213	4.825288	0.446451
C	3.943298	-4.580915	0.167824
H	4.231105	-5.568227	-0.199967
C	0.811916	2.471468	-0.370761
C	-2.794891	2.703333	0.371724
C	-1.454926	2.463408	-0.115465
C	-0.313446	3.296873	-0.266849
H	-0.323967	4.381543	-0.252010
C	4.503989	2.489932	-1.215191
H	5.232151	1.827909	-1.688558
C	-3.418297	3.968117	0.345730
H	-2.926773	4.789007	-0.178462
C	-4.640516	-4.173866	-0.973314
H	-5.117729	-5.153302	-0.937640
C	-4.657876	-1.851821	-1.706758
H	-5.131101	-1.024263	-2.235816
C	2.621406	-4.163476	0.034758
H	1.900214	-4.825312	-0.446391
C	3.181030	2.062787	-1.083503
H	2.893158	1.081333	-1.456749
C	-5.252999	3.110779	1.661643
H	-6.209327	3.268181	2.163024
C	-3.437916	1.620086	1.051632
C	-4.657868	1.851805	1.706773
H	-5.131086	1.024244	2.235832
C	-4.640524	4.173853	0.973338
H	-5.117741	5.153288	0.937672
C	-5.253001	-3.110797	-1.661618
H	-6.209333	-3.268204	-2.162990
H	6.822596	4.294686	0.100411
H	6.936630	3.509759	-1.490435
H	6.403795	5.204794	-1.357220
H	6.403772	-5.204710	1.357486
H	6.822563	-4.294943	-0.100359
H	6.936675	-3.509669	1.490311

1b

Imaginary Freq	0	
Temperature	298.150	Kelvin
Pressure	1.00000	atm
Frequencies scaled by	1.0000	
Electronic Energy (EE)	-1800.426880	Hartree
Zero-point Energy Correction	0.506095	Hartree
Thermal Correction to Energy	0.538360	Hartree
Thermal Correction to Enthalpy	0.539304	Hartree
Thermal Correction to Free Energy	0.441511	Hartree
EE + Zero-point Energy	-1799.920786	Hartree
EE + Thermal Energy Correction	-1799.888521	Hartree
EE + Thermal Enthalpy Correction	-1799.887576	Hartree
EE + Thermal Free Energy Correction	-1799.985369	Hartree
E (Thermal)	337.826	kcal/mol
Heat Capacity (Cv)	131.279	cal/mol-kelvin
Entropy (S)	205.823	cal/mol-kelvin

B	2.358032	-0.000000	-0.000000
O	-5.850579	4.077891	0.852432
O	3.244540	-0.370337	1.133354
N	-0.645566	0.000000	0.000000
N	1.426264	1.174739	0.215117
N	1.426263	-1.174739	-0.215118
O	3.244541	0.370337	-1.133354
C	-4.536387	3.759744	0.690236
C	-2.835577	-2.060781	-1.008421
H	-2.557031	-1.070371	-1.363805
O	-5.850580	-4.077890	-0.852432
C	3.156964	-2.696747	0.408352
C	1.814884	-2.465328	-0.079340
C	0.035159	-1.115608	-0.251274
C	-4.153513	-2.480389	-1.132230
H	-4.906178	-1.829367	-1.578094
C	3.782459	-3.960437	0.397101
H	3.290577	-4.788996	-0.114633
C	-0.452670	-2.477844	-0.332963
C	0.675630	-3.300968	-0.217602
H	0.688008	-4.385250	-0.186184
C	0.035159	1.115609	0.251274
C	-1.848484	-2.900476	-0.443330
C	-4.536388	-3.759744	-0.690236
C	-3.574377	4.611911	0.124042
H	-3.838637	5.604193	-0.234971
C	-1.848483	2.900477	0.443330
C	3.800338	-1.603676	1.071662
C	-2.253143	4.176874	0.009107

H	-1.526005	4.846903	-0.450844
C	-3.574378	-4.611910	-0.124042
H	-3.838637	-5.604192	0.234970
C	-0.452669	2.477845	0.332963
C	3.156965	2.696747	-0.408352
C	1.814885	2.465327	0.079340
C	0.675631	3.300969	0.217602
H	0.688009	4.385251	0.186183
C	-4.153512	2.480390	1.132231
H	-4.906177	1.829369	1.578096
C	3.782460	3.960436	-0.397101
H	3.290579	4.788996	0.114633
C	5.007196	-4.156251	1.023596
H	5.485679	-5.135449	0.999332
C	5.023064	-1.824715	1.725373
H	5.496746	-0.989362	2.241685
C	-2.253144	-4.176874	-0.009108
H	-1.526006	-4.846903	0.450843
C	-2.835575	2.060782	1.008422
H	-2.557030	1.070373	1.363806
C	-6.295041	5.371824	0.427710
H	-5.775173	6.168245	0.980227
H	-7.365429	5.405110	0.654833
H	-6.142716	5.505738	-0.653326
C	5.620260	3.083331	-1.695500
H	6.578448	3.232426	-2.195906
C	3.800339	1.603675	-1.071662
C	5.023065	1.824714	-1.725372
H	5.496747	0.989360	-2.241685
C	5.007198	4.156249	-1.023596
H	5.485681	5.135447	-0.999332
C	5.620258	-3.083333	1.695501
H	6.578446	-3.232428	2.195906
C	-6.295042	-5.371824	-0.427711
H	-6.142716	-5.505738	0.653325
H	-7.365430	-5.405110	-0.654834
H	-5.775174	-6.168244	-0.980228

2a

Imaginary Freq	0	
Temperature	298.150	Kelvin
Pressure	1.00000	atm
Frequencies scaled by	1.0000	
Electronic Energy (EE)	-2364.314438	Hartree
Zero-point Energy Correction	0.671516	Hartree
Thermal Correction to Energy	0.715441	Hartree
Thermal Correction to Enthalpy	0.716385	Hartree
Thermal Correction to Free Energy	0.587331	Hartree
EE + Zero-point Energy	-2363.642922	Hartree
EE + Thermal Energy Correction	-2363.598997	Hartree
EE + Thermal Enthalpy Correction	-2363.598053	Hartree
EE + Thermal Free Energy Correction	-2363.727107	Hartree
E (Thermal)	448.946	kcal/mol
Heat Capacity (Cv)	171.962	cal/mol-kelvin
Entropy (S)	271.617	cal/mol-kelvin

Al	-1.918981	-0.000003	-0.000322
C	7.257205	-3.495261	0.198355
O	-3.244461	1.288399	-0.019451
N	1.472151	0.000001	0.000201
N	-0.511583	-1.385720	0.024185
N	-0.511578	1.385720	-0.024032
O	-3.244470	-1.288408	0.018023
C	5.781456	-3.216364	0.183152
N	-1.969754	0.033718	2.121088
C	3.889165	1.768712	-0.692892
H	3.509007	0.845655	-1.126710
C	7.257213	3.495275	-0.197742
N	-1.968569	-0.033722	-2.121765
C	-2.008345	3.358851	-0.025370
C	-0.705089	2.725582	-0.035849
C	0.858984	1.169953	-0.046411
C	5.255124	2.040147	-0.727181
H	5.928794	1.319020	-1.196190
C	-2.099588	4.767897	-0.022061
H	-1.182028	5.356454	-0.024954
C	1.545983	2.438875	-0.080255
C	0.552035	3.399246	-0.066973
H	0.697083	4.473557	-0.109365
C	0.858977	-1.169950	0.046746
C	2.990168	2.675335	-0.109171
C	5.781461	3.216394	-0.182556
C	4.883419	-4.122058	-0.398365
H	5.261694	-5.049530	-0.834766
C	2.990157	-2.675332	0.109777

C	-3.214312	2.595075	-0.018331
C	3.519219	-3.858805	-0.435497
H	2.849141	-4.576501	-0.912027
C	4.883427	4.122037	0.399042
H	5.261702	5.049488	0.835490
C	1.545972	-2.438869	0.080793
C	-2.008350	-3.358854	0.025587
C	-0.705097	-2.725581	0.036166
C	0.552023	-3.399240	0.067535
H	0.697070	-4.473547	0.110026
C	5.255117	-2.040084	0.727701
H	5.928791	-1.318909	1.196633
C	-2.099594	-4.767902	0.023056
H	-1.182036	-5.356458	0.026730
C	-0.864160	-0.072786	-2.878633
H	0.087025	-0.087290	-2.348713
C	-3.315258	5.429497	-0.014079
H	-3.349404	6.519221	-0.011456
C	-4.445680	3.291770	-0.011222
H	-5.359303	2.694937	-0.006806
C	3.519228	3.858770	0.436181
H	2.849151	4.576433	0.912760
C	3.889154	-1.768662	0.693420
H	3.508998	-0.845569	1.127162
C	-2.146794	0.075750	4.899589
H	-2.216101	0.092067	5.988059
C	-4.498932	-4.675406	0.008927
H	-5.468180	-5.178523	0.002654
C	-3.214316	-2.595083	0.017617
C	-4.445680	-3.291784	0.010294
H	-5.359301	-2.694954	0.005114
C	-0.865716	0.072682	2.878502
H	0.085730	0.087109	2.349043
C	-3.161576	-0.015597	-2.733165
H	-4.026939	0.017026	-2.073404
C	-3.315259	-5.429508	0.014925
H	-3.349402	-6.519233	0.012948
C	-2.144236	-0.075763	-4.900357
H	-2.213006	-0.092085	-5.988862
C	-3.296682	0.035786	4.114700
H	-4.289519	0.019870	4.562562
C	-3.294509	-0.035693	-4.116038
H	-4.287125	-0.019695	-4.564389
C	-0.906282	0.094470	4.267088
H	0.022367	0.125709	4.835443
C	-4.498932	4.675391	-0.009086

H	-5.468183	5.178504	-0.002971
C	-0.904041	-0.094583	-4.267240
H	0.024888	-0.125907	-4.835132
C	-3.163069	0.015691	2.731891
H	-4.028119	-0.016846	2.071710
H	7.463078	4.544665	-0.449275
H	7.703745	3.306839	0.790562
H	7.776596	2.856320	-0.923336
H	7.463068	-4.544569	0.450240
H	7.776627	-2.856077	0.923720
H	7.703694	-3.307168	-0.790031

2b

Imaginary Freq	0	
Temperature	298.150	Kelvin
Pressure	1.00000	atm
Frequencies scaled by	1.0000	
Electronic Energy (EE)	-2514.752698	Hartree
Zero-point Energy Correction	0.681941	Hartree
Thermal Correction to Energy	0.727221	Hartree
Thermal Correction to Enthalpy	0.728166	Hartree
Thermal Correction to Free Energy	0.598381	Hartree
EE + Zero-point Energy	-2514.070757	Hartree
EE + Thermal Energy Correction	-2514.025477	Hartree
EE + Thermal Enthalpy Correction	-2514.024533	Hartree
EE + Thermal Free Energy Correction	-2514.154317	Hartree
E (Thermal)	456.338	kcal/mol
Heat Capacity (Cv)	178.257	cal/mol-kelvin
Entropy (S)	273.155	cal/mol-kelvin

Al	2.237488	-0.000000	0.000000
O	-6.810739	3.441466	0.392474
O	3.564831	-1.295626	-0.013127
N	-1.165775	0.000000	0.000000
N	0.826105	1.391940	0.018500
N	0.826105	-1.391940	-0.018500
O	3.564831	1.295625	0.013128
C	-5.461865	3.262600	0.274954
N	2.297958	-0.024944	2.153466
C	-3.585172	-1.803251	-0.762602
H	-3.204411	-0.883382	-1.200529
O	-6.810739	-3.441465	-0.392474
N	2.297958	0.024944	-2.153465
C	2.327767	-3.375492	-0.004073
C	1.020461	-2.738970	-0.025397
C	-0.552916	-1.175053	-0.049048
C	-4.946130	-2.079586	-0.831527

H	-5.630285	-1.386082	-1.322050
C	2.422962	-4.789104	0.012964
H	1.508319	-5.381175	0.014249
C	-1.239541	-2.450862	-0.088831
C	-0.240200	-3.412201	-0.065761
H	-0.384313	-4.485883	-0.110535
C	-0.552916	1.175053	0.049048
C	-2.685497	-2.697361	-0.140510
C	-5.461866	-3.262600	-0.274954
C	-4.590167	4.165485	-0.352436
H	-4.955692	5.086098	-0.802692
C	-2.685497	2.697362	0.140510
C	3.538246	-2.609652	-0.000429
C	-3.224446	3.874660	-0.410436
H	-2.565056	4.580120	-0.917784
C	-4.590167	-4.165485	0.352436
H	-4.955692	-5.086098	0.802690
C	-1.239540	2.450862	0.088831
C	2.327767	3.375491	0.004072
C	1.020462	2.738970	0.025397
C	-0.240199	3.412201	0.065761
H	-0.384313	4.485883	0.110535
C	-4.946129	2.079587	0.831527
H	-5.630284	1.386083	1.322051
C	2.422963	4.789104	-0.012964
H	1.508320	5.381175	-0.014251
C	1.194000	0.060724	-2.921616
H	0.239416	0.077192	-2.400873
C	3.644034	-5.449528	0.030491
H	3.679898	-6.539163	0.044075
C	4.774799	-3.304931	0.015774
H	5.688062	-2.707759	0.016908
C	-3.224447	-3.874660	0.410435
H	-2.565056	-4.580120	0.917783
C	-3.585172	1.803251	0.762603
H	-3.204410	0.883383	1.200530
C	2.487611	-0.055489	4.944086
H	2.561532	-0.067290	6.032126
C	-7.384385	4.635664	-0.148570
H	-6.965282	5.530393	0.335501
H	-8.456636	4.571845	0.063876
H	-7.227391	4.692800	-1.236056
C	4.830663	4.692559	-0.031014
H	5.800249	5.194650	-0.044270
C	3.538246	2.609652	0.000429
C	4.774800	3.304930	-0.015773

H	5.688062	2.707759	-0.016908
C	1.194000	-0.060723	2.921616
H	0.239416	-0.077192	2.400873
C	3.497722	0.004615	-2.764627
H	4.362774	-0.024765	-2.106199
C	3.644035	5.449527	-0.030492
H	3.679899	6.539163	-0.044076
C	2.487612	0.055489	-4.944085
H	2.561533	0.067290	-6.032126
C	3.636714	-0.018968	4.150934
H	4.631401	-0.001444	4.594709
C	3.636715	0.018967	-4.150933
H	4.631401	0.001443	-4.594708
C	1.241564	-0.076654	4.314105
H	0.315406	-0.105436	4.886703
C	4.830663	-4.692559	0.031014
H	5.800248	-5.194651	0.044270
C	-7.384385	-4.635664	0.148569
H	-7.227391	-4.692801	1.236056
C	1.241565	0.076654	-4.314104
H	0.315407	0.105437	-4.886703
C	3.497721	-0.004616	2.764628
H	4.362774	0.024765	2.106200
H	-8.456636	-4.571845	-0.063876
H	-6.965282	-5.530393	-0.335502

3a

Imaginary Freq	0	
Temperature	298.150	Kelvin
Pressure	1.00000	atm
Frequencies scaled by	1.0000	
Electronic Energy (EE)	-4046.646586	Hartree
Zero-point Energy Correction	0.669446	Hartree
Thermal Correction to Energy	0.714323	Hartree
Thermal Correction to Enthalpy	0.715267	Hartree
Thermal Correction to Free Energy	0.582304	Hartree
EE + Zero-point Energy	-4045.977139	Hartree
EE + Thermal Energy Correction	-4045.932263	Hartree
EE + Thermal Enthalpy Correction	-4045.931318	Hartree
EE + Thermal Free Energy Correction	-4046.064282	Hartree
E (Thermal)	448.244	kcal/mol
Heat Capacity (Cv)	173.375	cal/mol-kelvin
Entropy (S)	279.845	cal/mol-kelvin

Ga	-1.832979	0.000000	0.000000
C	7.386492	3.533959	-0.344098
O	-3.229624	-1.346860	-0.033122

N	1.576754	-0.000000	-0.000000
N	-0.402449	1.414593	0.017391
N	-0.402450	-1.414593	-0.017391
O	-3.229623	1.346861	0.033122
C	5.905371	3.247686	-0.279923
N	-1.926884	0.042989	-2.220100
C	3.984798	-1.831027	0.804553
H	3.586183	-0.941617	1.287980
C	7.386491	-3.533960	0.344098
N	-1.926884	-0.042989	2.220100
C	-1.904728	-3.397440	-0.077397
C	-0.598278	-2.757615	-0.034413
C	0.972461	-1.180151	0.028467
C	5.352031	-2.108466	0.883376
H	6.002089	-1.424286	1.433395
C	-1.970860	-4.814013	-0.125332
H	-1.042411	-5.383522	-0.133542
C	1.662322	-2.454585	0.052338
C	0.670531	-3.421905	0.004472
H	0.826161	-4.494469	0.032162
C	0.972461	1.180151	-0.028467
C	3.110362	-2.694262	0.117283
C	5.905371	-3.247687	0.279922
C	5.032740	4.109769	0.406977
H	5.430388	5.004830	0.890883
C	3.110363	2.694262	-0.117283
C	-3.137252	-2.660181	-0.072819
C	3.667160	3.841164	0.487080
H	3.020870	4.523364	1.041438
C	5.032739	-4.109770	-0.406977
H	5.430387	-5.004830	-0.890883
C	1.662322	2.454585	-0.052338
C	-1.904728	3.397440	0.077397
C	-0.598277	2.757615	0.034413
C	0.670532	3.421905	-0.004472
H	0.826162	4.494469	-0.032162
C	5.352032	2.108465	-0.883376
H	6.002089	1.424285	-1.433395
C	-1.970859	4.814014	0.125332
H	-1.042410	5.383523	0.133542
C	-0.827587	0.031973	2.989562
H	0.125733	0.111066	2.470654
C	-3.172087	-5.506092	-0.165362
H	-3.178634	-6.595653	-0.202679
C	-4.354325	-3.392715	-0.112226
H	-5.281926	-2.818138	-0.106593

C	3.667159	-3.841164	-0.487080
H	3.020870	-4.523365	-1.041438
C	3.984798	1.831026	-0.804553
H	3.586184	0.941616	-1.287980
C	-2.127652	0.088319	-5.004006
H	-2.206207	0.105928	-6.091645
C	-4.377599	4.779078	0.157514
H	-5.334924	5.303449	0.187969
C	-3.137251	2.660181	0.072819
C	-4.354325	3.392715	0.112226
H	-5.281925	2.818139	0.106594
C	-0.827587	-0.031971	-2.989562
H	0.125733	-0.111063	-2.470654
C	-3.126778	-0.140710	2.819496
H	-3.986057	-0.202211	2.155201
C	-3.172086	5.506092	0.165362
H	-3.178633	6.595654	0.202679
C	-2.127651	-0.088319	5.004006
H	-2.206206	-0.105928	6.091645
C	-3.271256	0.166086	-4.205612
H	-4.264791	0.245863	-4.645203
C	-3.271255	-0.166087	4.205613
H	-4.264790	-0.245865	4.645204
C	-0.881067	-0.012218	-4.382147
H	0.039509	-0.075367	-4.961057
C	-4.377600	-4.779078	-0.157514
H	-5.334925	-5.303448	-0.187969
C	-0.881067	0.012220	4.382147
H	0.039509	0.075370	4.961057
C	-3.126779	0.140709	-2.819495
H	-3.986057	0.202208	-2.155200
H	7.578959	-4.594986	0.557890
H	7.875814	-3.303951	-0.615442
H	7.875291	-2.929749	1.119861
H	7.578959	4.594985	-0.557889
H	7.875291	2.929748	-1.119862
H	7.875814	3.303949	0.615441

3b

Imaginary Freq	0	
Temperature	298.150	Kelvin
Pressure	1.00000	atm
Frequencies scaled by	1.0000	
Electronic Energy (EE)	-4197.084828	Hartree
Zero-point Energy Correction	0.679741	Hartree
Thermal Correction to Energy	0.726045	Hartree
Thermal Correction to Enthalpy	0.726989	Hartree
Thermal Correction to Free Energy	0.592356	Hartree
EE + Zero-point Energy	-4196.405087	Hartree
EE + Thermal Energy Correction	-4196.358783	Hartree
EE + Thermal Enthalpy Correction	-4196.357839	Hartree
EE + Thermal Free Energy Correction	-4196.492472	Hartree
E (Thermal)	455.600	kcal/mol
Heat Capacity (Cv)	179.724	cal/mol-kelvin
Entropy (S)	283.359	cal/mol-kelvin

Ga	2.140625	-0.000001	0.000001
O	-6.931023	-3.415960	-0.400335
O	3.538409	1.346295	0.029011
N	-1.267770	0.000000	-0.000002
N	0.711317	-1.414325	-0.025632
N	0.711318	1.414325	0.025630
O	3.538408	-1.346297	-0.029007
C	-5.581231	-3.244178	-0.283250
N	2.235618	0.043677	-2.221085
C	-3.696917	1.795329	0.772585
H	-3.311441	0.877917	1.211665
O	-6.931020	3.415966	0.400333
N	2.235612	-0.043678	2.221086
C	2.214497	3.397861	0.017996
C	0.907069	2.757801	0.037597
C	-0.664208	1.179731	0.054333
C	-5.059330	2.064494	0.841118
H	-5.739862	1.367819	1.332168
C	2.281649	4.814967	0.001007
H	1.353454	5.384977	-0.001776
C	-1.354880	2.453925	0.098649
C	-0.360682	3.421372	0.080310
H	-0.514910	4.493324	0.129896
C	-0.664209	-1.179731	-0.054336
C	-2.802134	2.693679	0.149711
C	-5.581229	3.244183	0.283247
C	-4.714333	-4.150984	0.345133
H	-5.084766	-5.069049	0.796570
C	-2.802135	-2.693678	-0.149715

C	3.446658	2.660229	0.015613
C	-3.347058	-3.867432	0.402734
H	-2.691381	-4.575630	0.911081
C	-4.714329	4.150989	-0.345135
H	-5.084761	5.069054	-0.796571
C	-1.354882	-2.453924	-0.098652
C	2.214495	-3.397863	-0.017996
C	0.907067	-2.757802	-0.037598
C	-0.360685	-3.421372	-0.080312
H	-0.514913	-4.493324	-0.129898
C	-5.059331	-2.064490	-0.841122
H	-5.739862	-1.367815	-1.332172
C	2.281646	-4.814969	-0.001007
H	1.353450	-5.384978	0.001774
C	1.133129	-0.079374	2.988768
H	0.177322	-0.087295	2.468445
C	3.483444	5.507368	-0.015146
H	3.490524	6.597508	-0.028926
C	4.664285	3.392825	0.001003
H	5.591529	2.817609	0.001099
C	-3.347055	3.867435	-0.402737
H	-2.691378	4.575633	-0.911083
C	-3.696918	-1.795326	-0.772589
H	-3.311441	-0.877915	-1.211670
C	2.436142	0.094601	-5.004891
H	2.514603	0.114379	-6.092506
C	-7.511041	-4.606243	0.142601
H	-7.096816	-5.503948	-0.340145
H	-8.582953	-4.536952	-0.069825
H	-7.354215	-4.662542	1.230155
C	4.688587	-4.779898	0.014068
H	5.646331	-5.304298	0.026111
C	3.446656	-2.660232	-0.015610
C	4.664283	-3.392829	-0.000999
H	5.591527	-2.817613	-0.001093
C	1.133136	0.079352	-2.988769
H	0.177328	0.087257	-2.468449
C	3.438522	-0.033297	2.822132
H	4.300558	-0.003072	2.159279
C	3.483441	-5.507370	0.015147
H	3.490520	-6.597511	0.028926
C	2.436129	-0.094603	5.004894
H	2.514587	-0.114382	6.092509
C	3.583047	0.058160	-4.208270
H	4.579131	0.048667	-4.649233
C	3.583036	-0.058140	4.208275

H	4.579118	-0.048630	4.649242
C	1.186393	0.105228	-4.381306
H	0.263212	0.133330	-4.958844
C	4.688590	4.779895	-0.014065
H	5.646334	5.304294	-0.026106
C	-7.511037	4.606250	-0.142601
H	-7.354212	4.662550	-1.230156
C	1.186383	-0.105251	4.381305
H	0.263201	-0.133371	4.958840
C	3.438529	0.033317	-2.822126
H	4.300564	0.003109	-2.159271
H	-8.582949	4.536961	0.069825
H	-7.096811	5.503954	0.340145

4a

Imaginary Freq	0	
Temperature	298.150	Kelvin
Pressure	1.00000	atm
Frequencies scaled by	1.0000	
Electronic Energy (EE)	-2123.677773	Hartree
Zero-point Energy Correction	0.668011	Hartree
Thermal Correction to Energy	0.713478	Hartree
Thermal Correction to Enthalpy	0.714423	Hartree
Thermal Correction to Free Energy	0.580827	Hartree
EE + Zero-point Energy	-2123.009762	Hartree
EE + Thermal Energy Correction	-2122.964295	Hartree
EE + Thermal Enthalpy Correction	-2122.963350	Hartree
EE + Thermal Free Energy Correction	-2123.096946	Hartree
E (Thermal)	447.714	kcal/mol
Heat Capacity (Cv)	174.185	cal/mol-kelvin
Entropy (S)	281.175	cal/mol-kelvin

In	1.870064	-0.000000	0.000001
C	-7.528220	3.381782	0.514950
O	3.206026	-1.520672	-0.002893
N	-1.650367	0.000000	-0.000000
N	0.303449	1.461927	-0.002767
N	0.303449	-1.461927	0.002768
O	3.206026	1.520672	0.002895
C	-6.042668	3.129788	0.416172
N	1.967006	-0.002528	2.354899
C	-4.068044	-1.789066	-0.938496
H	-3.630408	-0.930920	-1.444936
C	-7.528220	-3.381782	-0.514953
N	1.967008	0.002528	-2.354898
C	1.756745	-3.495333	0.055207
C	0.466185	-2.809255	0.003901

C	-1.065086	-1.190431	-0.050744
C	-5.440667	-2.030152	-1.045145
H	-6.055817	-1.348396	-1.636692
C	1.752906	-4.913962	0.114843
H	0.796737	-5.434651	0.128701
C	-1.786145	-2.447820	-0.103450
C	-0.821062	-3.441111	-0.057959
H	-1.009040	-4.507841	-0.106079
C	-1.065086	1.190431	0.050744
C	-3.238484	-2.650416	-0.196628
C	-6.042669	-3.129787	-0.416175
C	-5.214196	3.990756	-0.325190
H	-5.651132	4.855039	-0.830828
C	-3.238484	2.650416	0.196626
C	3.033533	-2.827964	0.047673
C	-3.843786	3.758707	-0.432476
H	-3.231240	4.437660	-1.027842
C	-5.214196	-3.990756	0.325188
H	-5.651132	-4.855039	0.830826
C	-1.786145	2.447820	0.103449
C	1.756746	3.495333	-0.055207
C	0.466185	2.809255	-0.003900
C	-0.821062	3.441111	0.057959
H	-1.009040	4.507841	0.106079
C	-5.440667	2.030153	1.045143
H	-6.055817	1.348396	1.636690
C	1.752906	4.913961	-0.114843
H	0.796738	5.434651	-0.128701
C	0.856059	0.020452	-3.113137
H	-0.094925	0.036417	-2.582642
C	2.913501	-5.672050	0.162257
H	2.857792	-6.759892	0.209407
C	4.206306	-3.629043	0.093261
H	5.163496	-3.105030	0.084117
C	-3.843786	-3.758706	0.432474
H	-3.231241	-4.437659	1.027841
C	-4.068044	1.789066	0.938494
H	-3.630408	0.930921	1.444934
C	2.137620	0.001459	5.142945
H	2.204177	0.003172	6.231508
C	4.156770	5.014268	-0.149500
H	5.084469	5.589106	-0.185204
C	3.033533	2.827963	-0.047671
C	4.206307	3.629043	-0.093258
H	5.163496	3.105029	-0.084114
C	0.856058	-0.020448	3.113138

H	-0.094927	-0.036411	2.582643
C	3.163081	-0.016901	-2.971347
H	4.038965	-0.031019	-2.323976
C	2.913502	5.672049	-0.162256
H	2.857793	6.759892	-0.209406
C	2.137622	-0.001458	-5.142944
H	2.204180	-0.003172	-6.231506
C	3.293703	0.019272	4.359005
H	4.285084	0.035127	4.810200
C	3.293705	-0.019275	-4.359003
H	4.285087	-0.035133	-4.810197
C	0.894504	-0.018701	4.506427
H	-0.034720	-0.033198	5.074690
C	4.156769	-5.014268	0.149502
H	5.084468	-5.589106	0.185207
C	0.894506	0.018705	-4.506426
H	-0.034718	0.033204	-5.074689
C	3.163079	0.016898	2.971349
H	4.038964	0.031013	2.323979
H	-7.737642	-4.418875	-0.815063
H	-8.022729	-3.221825	0.455707
H	-7.997862	-2.710099	-1.245635
H	-7.737642	4.418876	0.815058
H	-7.997862	2.710101	1.245632
H	-8.022729	3.221823	-0.455710

4b

Imaginary Freq	0	
Temperature	298.150	Kelvin
Pressure	1.00000	atm
Frequencies scaled by	1.0000	
Electronic Energy (EE)	-2274.115932	Hartree
Zero-point Energy Correction	0.678338	Hartree
Thermal Correction to Energy	0.725240	Hartree
Thermal Correction to Enthalpy	0.726185	Hartree
Thermal Correction to Free Energy	0.590678	Hartree
EE + Zero-point Energy	-2273.437595	Hartree
EE + Thermal Energy Correction	-2273.390692	Hartree
EE + Thermal Enthalpy Correction	-2273.389748	Hartree
EE + Thermal Free Energy Correction	-2273.525254	Hartree
E (Thermal)	455.095	kcal/mol
Heat Capacity (Cv)	180.512	cal/mol-kelvin
Entropy (S)	285.198	cal/mol-kelvin

In	-2.287835	0.000005	0.000003
O	6.959919	-3.270370	0.574065
O	-3.626027	1.518222	-0.048641

N	1.230054	-0.000001	-0.000006
N	-0.723071	-1.461298	0.044851
N	-0.723066	1.461303	-0.044852
O	-3.626032	-1.518208	0.048653
C	5.608674	-3.131520	0.429209
N	-2.385802	0.073950	2.354747
C	3.680432	1.727293	-0.875118
H	3.263893	0.818127	-1.303024
O	6.959927	3.270347	-0.574112
N	-2.385813	-0.073940	-2.354740
C	-2.177950	3.494772	-0.066539
C	-0.886005	2.808413	-0.085569
C	0.646110	1.189466	-0.080274
C	5.047384	1.963240	-0.972426
H	5.700903	1.248654	-1.474318
C	-2.175691	4.914539	-0.064732
H	-1.220042	5.436294	-0.070857
C	1.368426	2.445647	-0.161705
C	0.400000	3.438660	-0.156310
H	0.585659	4.503876	-0.234971
C	0.646106	-1.189467	0.080265
C	2.820349	2.649221	-0.238201
C	5.608682	3.131502	-0.429248
C	4.776929	-4.060990	-0.213250
H	5.178669	-4.971166	-0.653818
C	2.820341	-2.649229	0.238179
C	-3.454189	2.826701	-0.050523
C	3.404424	-3.810994	-0.299109
H	2.775899	-4.536580	-0.817244
C	4.776945	4.060975	0.213217
H	5.178690	4.971149	0.653784
C	1.368418	-2.445650	0.161691
C	-2.177962	-3.494762	0.066547
C	-0.886015	-2.808408	0.085569
C	0.399989	-3.438660	0.156303
H	0.585644	-4.503876	0.234963
C	5.047383	-1.963255	0.972390
H	5.700907	-1.248672	1.474278
C	-2.175708	-4.914530	0.064741
H	-1.220061	-5.436288	0.070862
C	-1.275642	-0.132357	-3.112019
H	-0.324751	-0.145093	-2.581203
C	-3.337014	5.673134	-0.051111
H	-3.282111	6.762060	-0.048840
C	-4.627713	3.627970	-0.039314
H	-5.584396	3.102998	-0.029245

C	3.404439	3.810983	0.299084
H	2.775920	4.536571	0.817224
C	3.680431	-1.727304	0.875090
H	3.263898	-0.818136	1.302998
C	-2.557766	0.157633	5.141465
H	-2.624858	0.190275	6.229510
C	7.578864	-4.447818	0.046748
H	7.176748	-5.353941	0.524040
H	8.644415	-4.352335	0.280094
H	7.445140	-4.511133	-1.043543
C	-4.579709	-5.014417	0.039347
H	-5.508002	-5.589335	0.029076
C	-3.454198	-2.826687	0.050536
C	-4.627726	-3.627952	0.039335
H	-5.584407	-3.102977	0.029270
C	-1.275627	0.132365	3.112020
H	-0.324738	0.145099	2.581200
C	-3.581747	-0.056867	-2.971430
H	-4.456798	-0.008095	-2.324599
C	-3.337033	-5.673121	0.051127
H	-3.282135	-6.762047	0.048857
C	-2.557790	-0.157623	-5.141458
H	-2.624887	-0.190264	-6.229503
C	-3.713071	0.097541	4.358468
H	-4.704342	0.081773	4.809926
C	-3.713092	-0.097528	-4.358456
H	-4.704365	-0.081759	-4.809909
C	-1.314741	0.175148	4.504668
H	-0.386120	0.221476	5.072225
C	-4.579692	5.014435	-0.039325
H	-5.507983	5.589356	-0.029049
C	7.578879	4.447793	-0.046797
H	7.445162	4.511107	1.043494
C	-1.314762	-0.175140	-4.504667
H	-0.386144	-0.221470	-5.072228
C	-3.581733	0.056879	2.971442
H	-4.456787	0.008108	2.324615
H	8.644428	4.352307	-0.280150
H	7.176763	5.353917	-0.524087

UV-Vis Calculations with TD-DFT[2]

Theory Level: **1a-b**, **2a-b** and **3a-b:** TD-DFT (B3LYP/jul-cc-pvDz) // DFT(B3LYP/jul-cc-pvDz)
4a-b: TD-DFT (B3LYP/LANL2DZ) // DFT (B3LYP/LANL2DZ)

Structure	Solvent	λ_{max} (nm)	Excitation Energy (eV)	Oscillator Strength
**1a**	Methanol	651.99	1.9016	0.5438
**1a**	Methanol	664.42	1.8661	0.5858
**2a**	Methanol	689.14	1.7991	0.5213
**2b**	Methanol	689.19	1.7990	0.552
**3a**	Methanol	687.73	1.8028	0.5241
**3b**	Methanol	688.02	1.8021	0.5539
**4a**	Methanol	661.47	1.8744	0.6207
**4b**	Methanol	664.89	1.8647	0.646

Table S1. UV-Vis of **1a-b**, **2a-b**, **3a-b**, and **4a-b** in MeOH as implicit solvent.

Structure	HOMO (eV)	LUMO (eV)
**1a**	-5.56	-3.66
**1b**	-5.21	-3.43
**2a**	-5.00	-3.20
**2b**	-4.97	-3.17
**3a**	-5.01	-3.21
**3b**	-4.98	-3.17
**4a**	-5.01	-3.14
**4b**	-4.98	-3.11

Table S2. HOMO and LUMO energies of **1a-b**, **2a-b**, **3a-b**, and **4a-b** in MeOH as implicit solvent.

Molecular Orbital Diagrams

Theory Level: **1a-b**, **2a-b**, and **3a-b:** TD-DFT (B3LYP/jul-cc-pvDz) // DFT(B3LYP/jul-cc-pvDz)
4a-b: TD-DFT (B3LYP/LANL2DZ) // DFT (B3LYP/LANL2DZ)

Notes: Natural Transition Orbitals (NTO) calculated for the first excited state during the TD-DFT calculations. HOMO and LUMO visualized using GaussView.

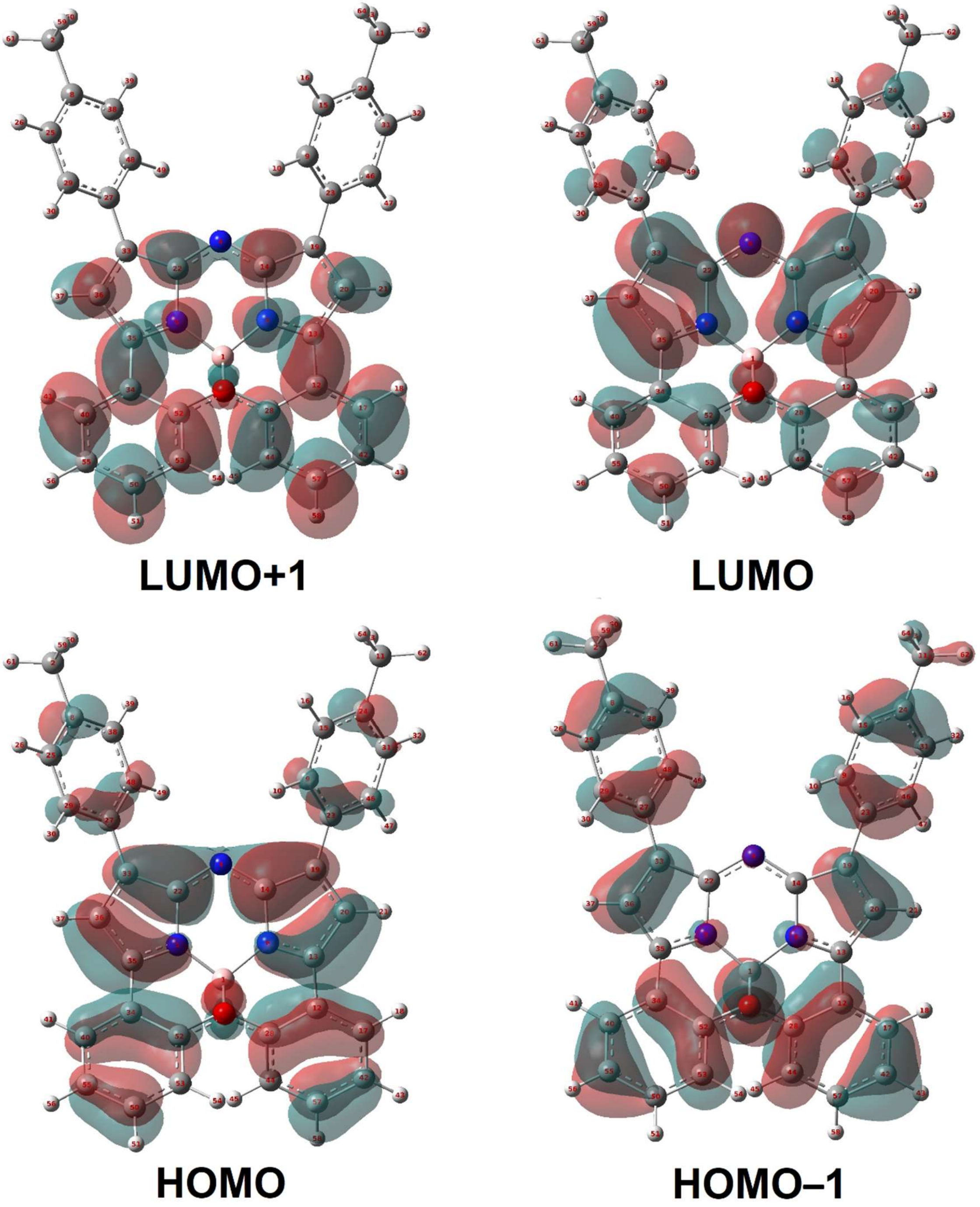

Figure S1. Frontier Molecular Orbital Plots of **1a**.

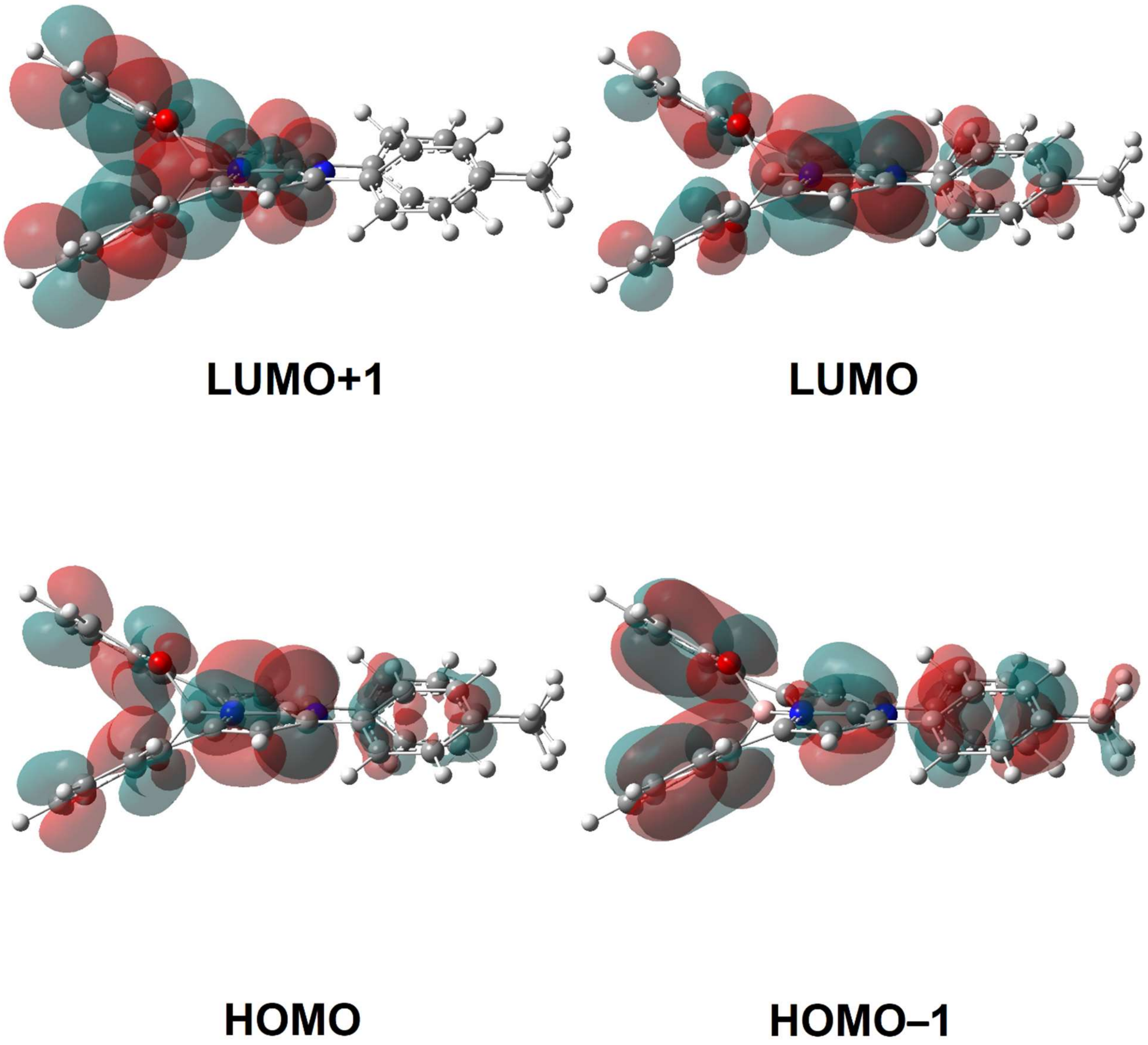

Figure S2. Horizontal view of Frontier Molecular Orbital Plots of **1a**.

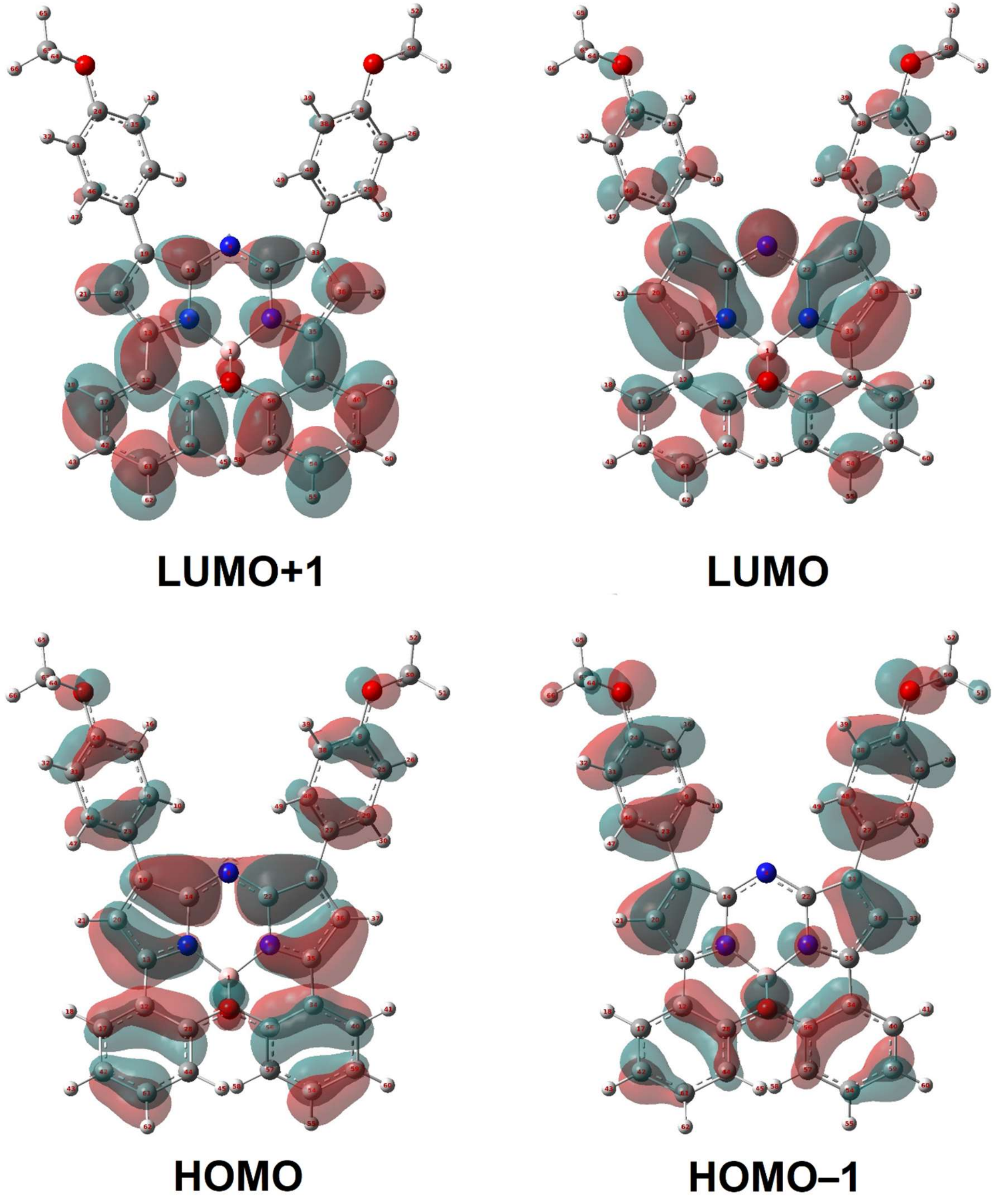

Figure S3. Frontier Molecular Orbital Plots of **1b**.

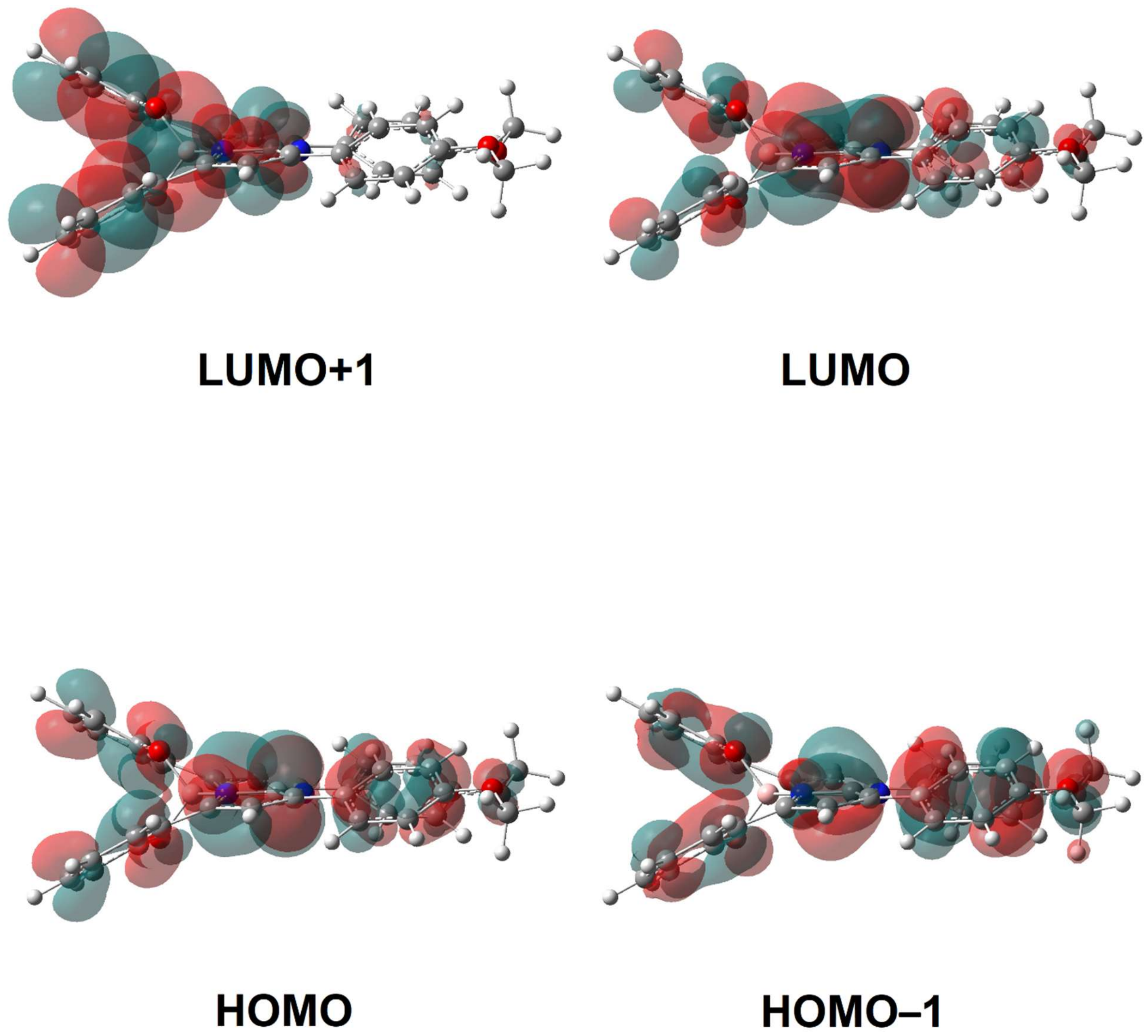

Figure S4. Horizontal view of Frontier Molecular Orbital Plots of **1b**.

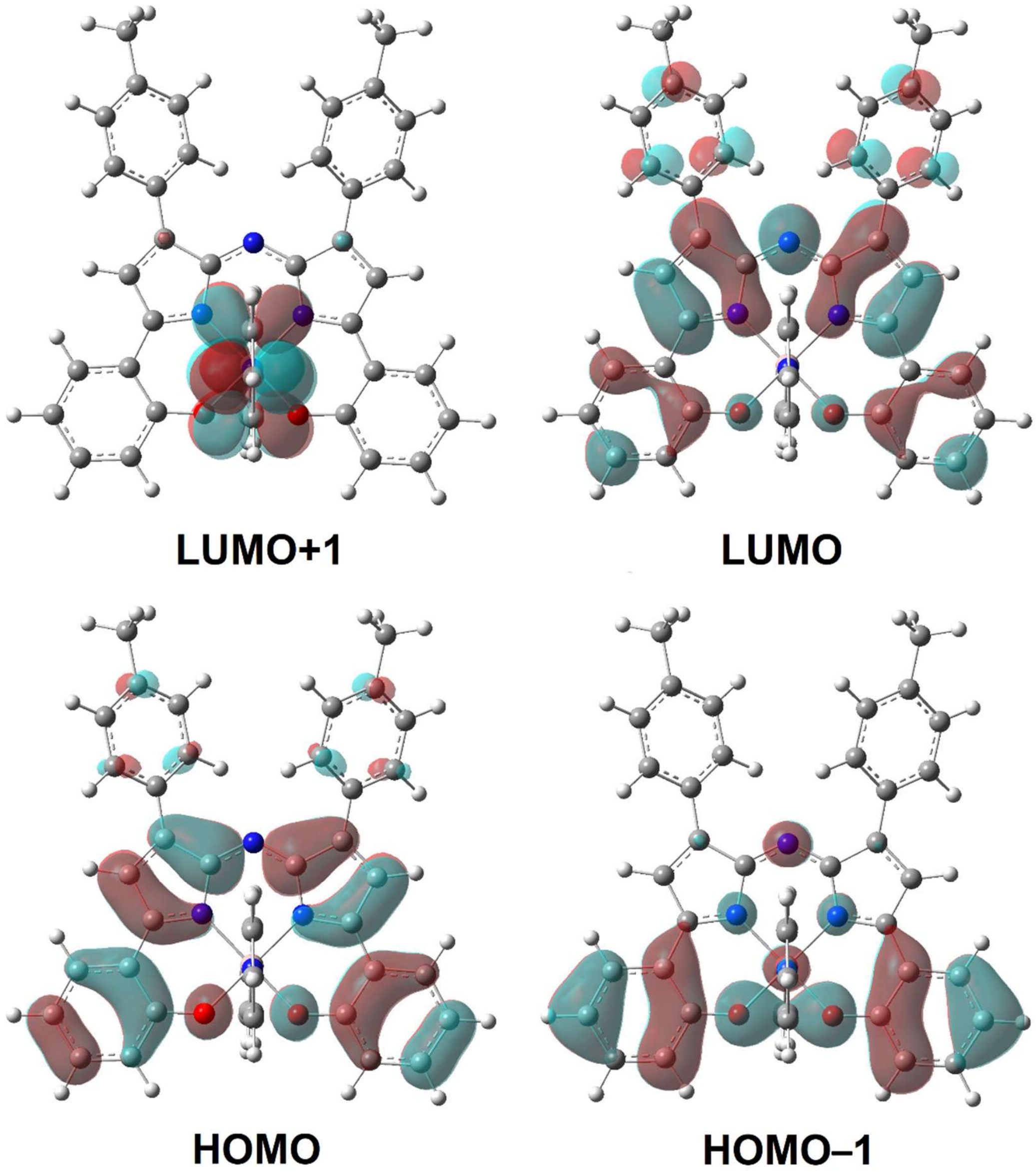

Figure S5. Frontier Molecular Orbital Plots of **2a**.

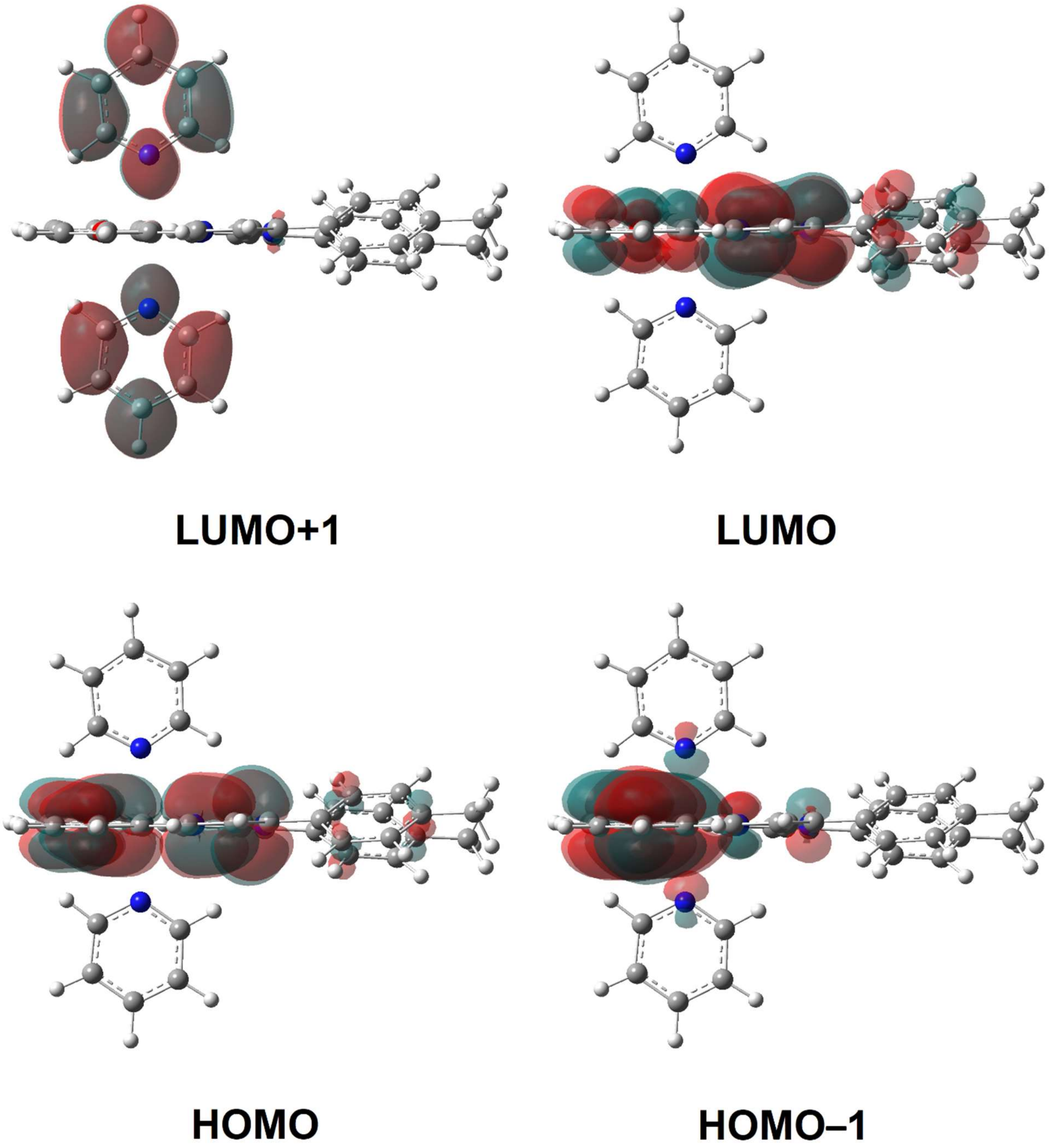

Figure S6. Horizontal view of Frontier Molecular Orbital Plots of **2a**.

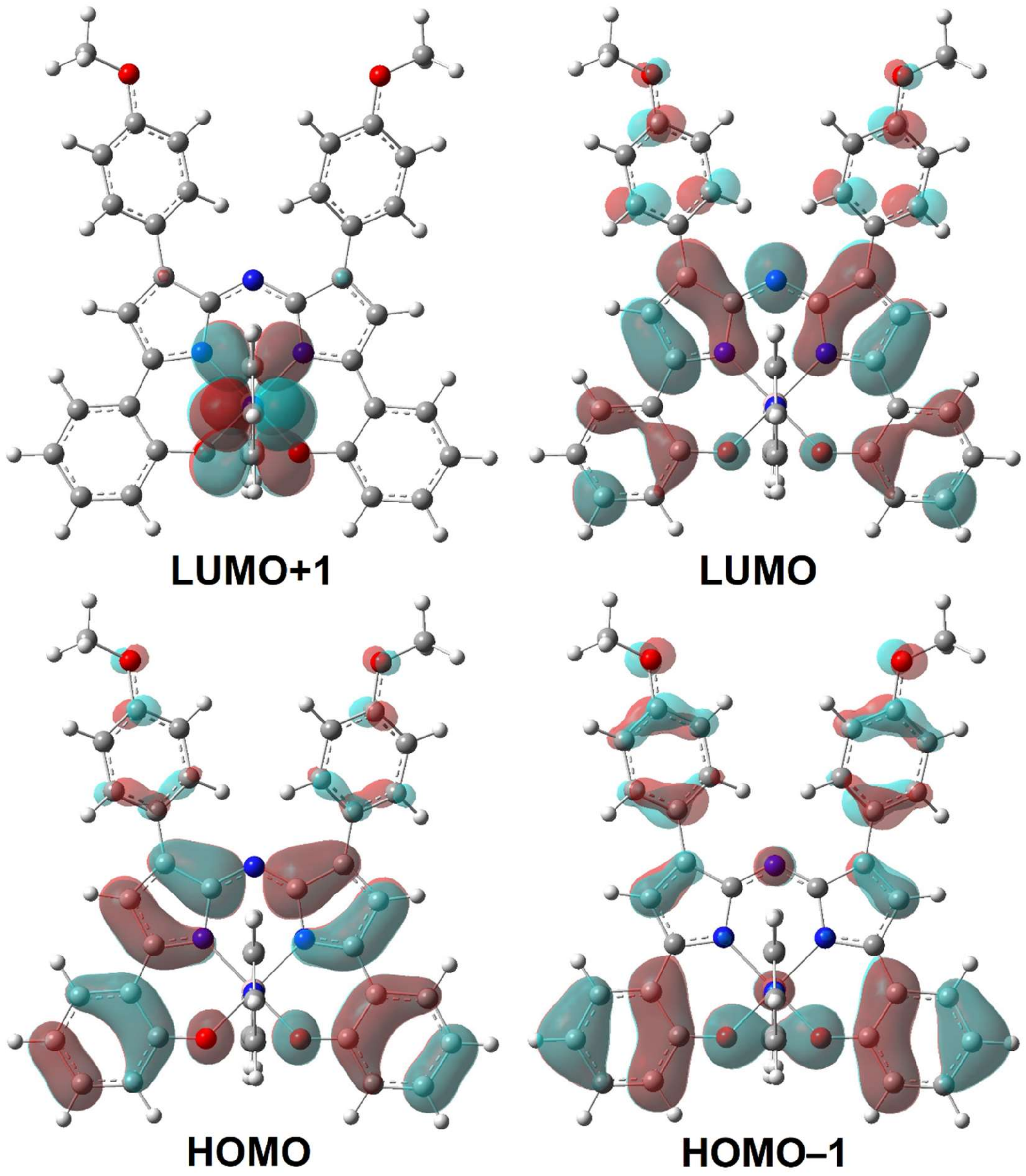

Figure S7. Frontier Molecular Orbital Plots of **2b**.

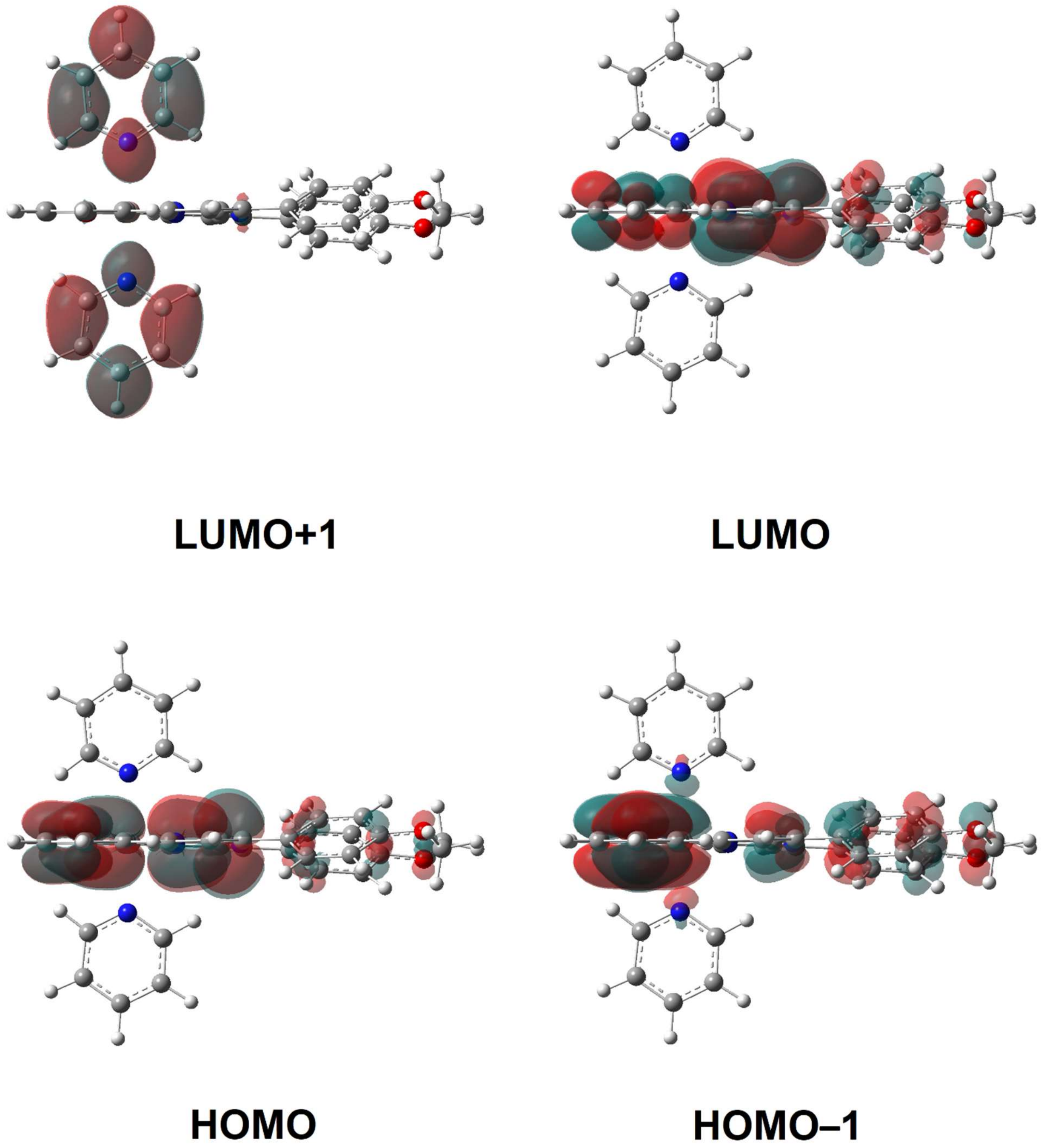

Figure S8. Horizontal view of Frontier Molecular Orbital Plots of **2b**.

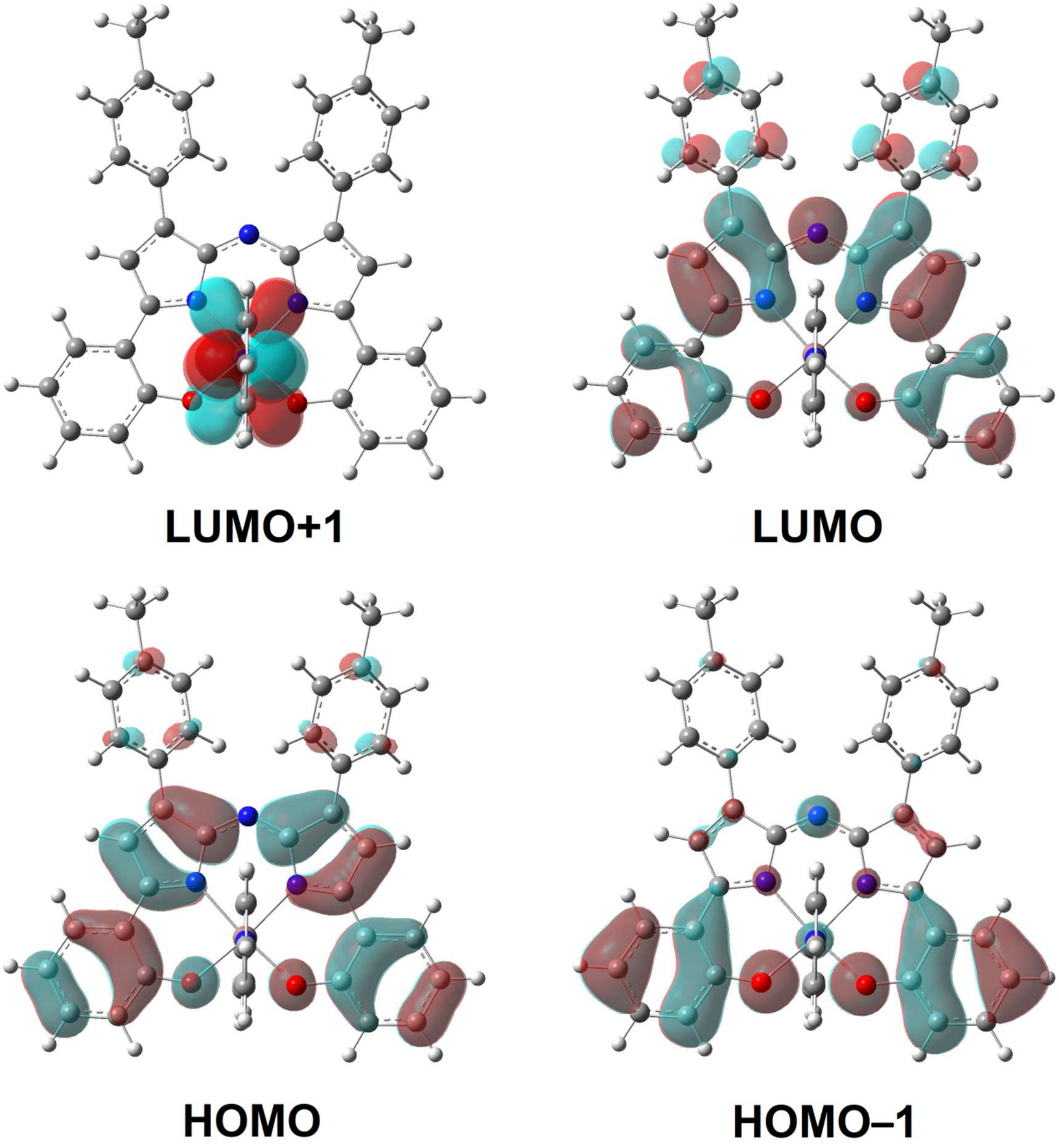

Figure S9. Frontier Molecular Orbital Plots of **3a**.

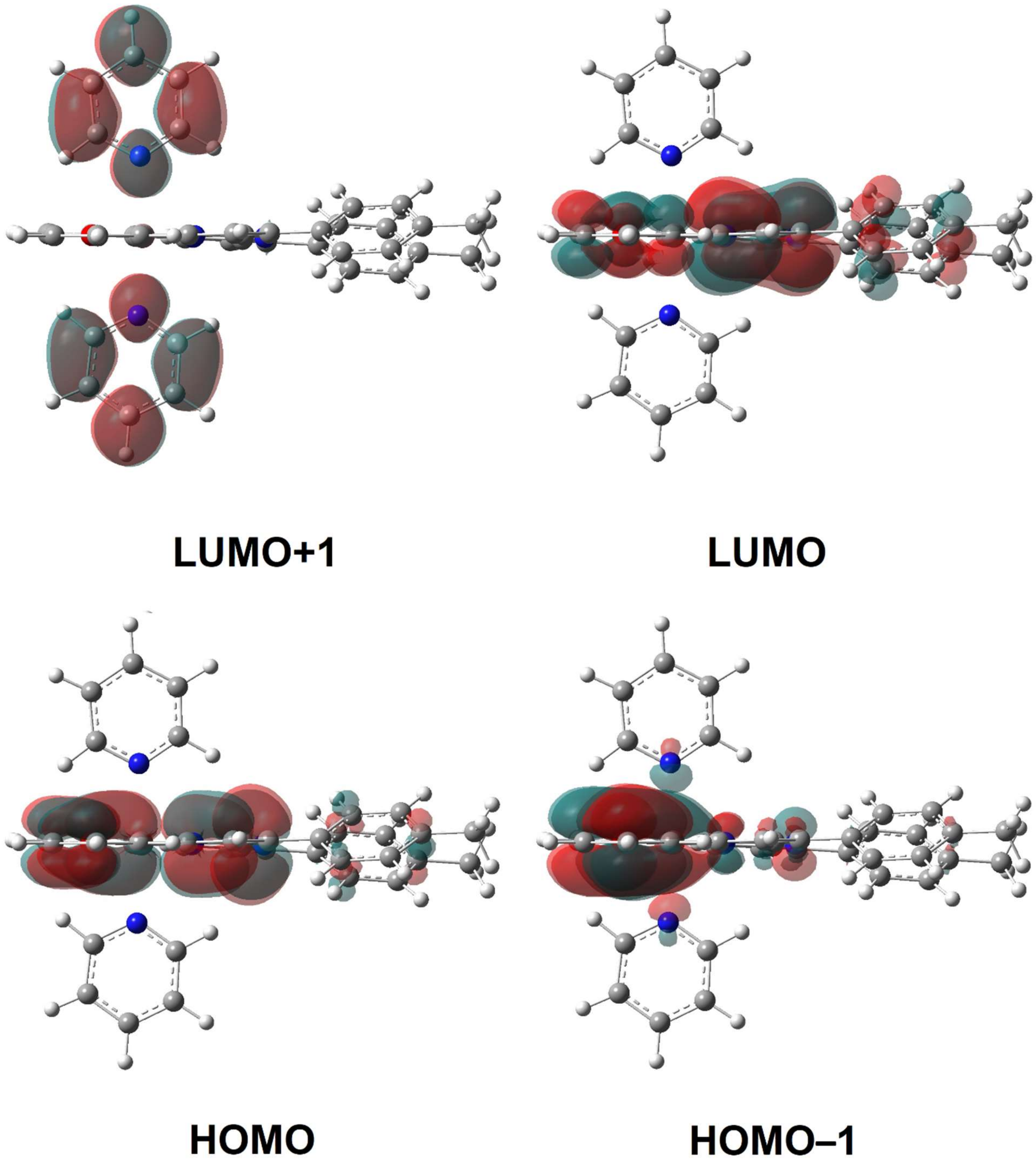

Figure S10. Horizontal view of Frontier Molecular Orbital Plots of **3a**.

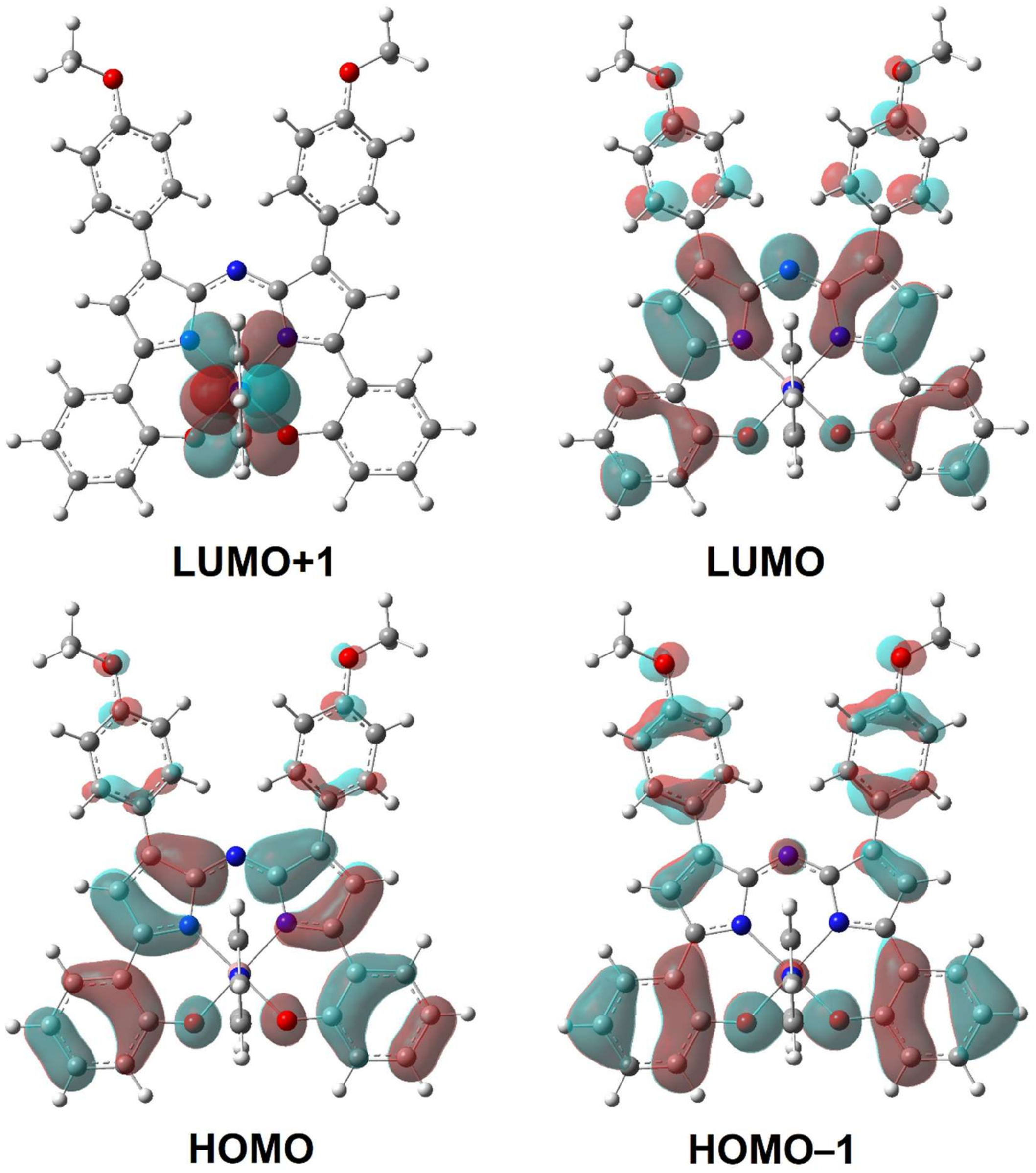

Figure S11. Frontier Molecular Orbital Plots of **3b**.

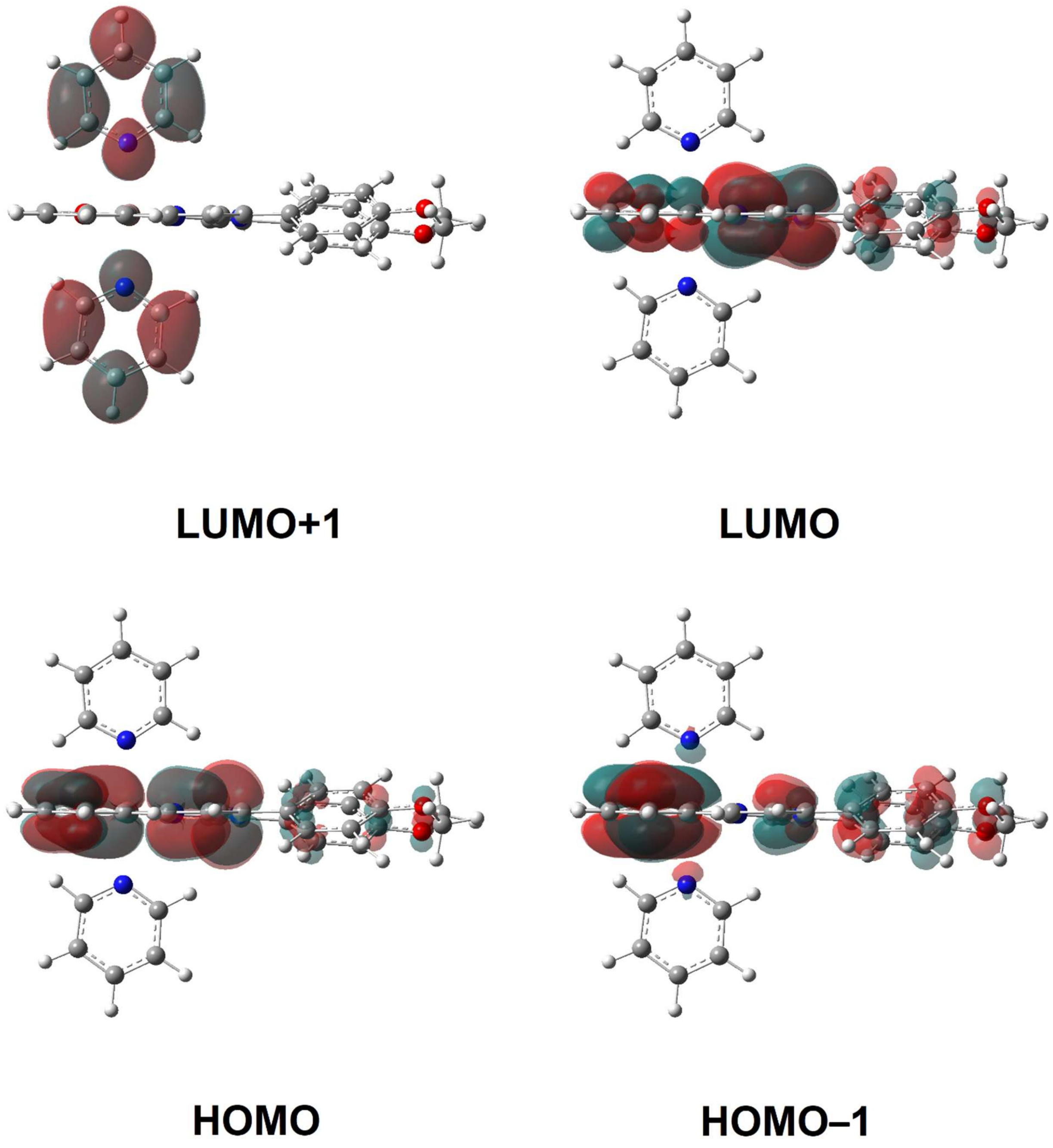

Figure S12. Horizontal view of Frontier Molecular Orbital Plots of **3b**.

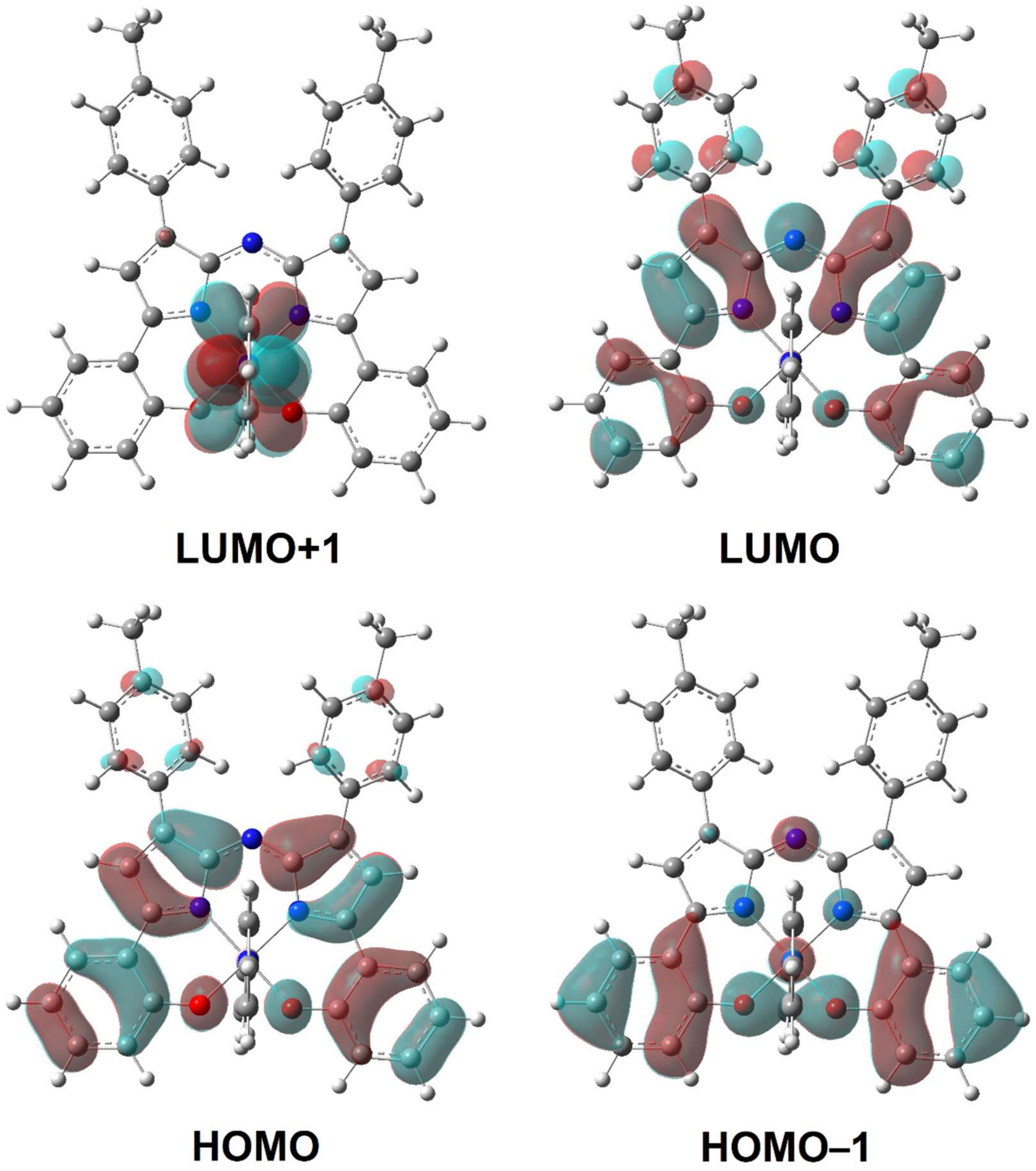

Figure S13. Frontier Molecular Orbital Plots of **4a**.

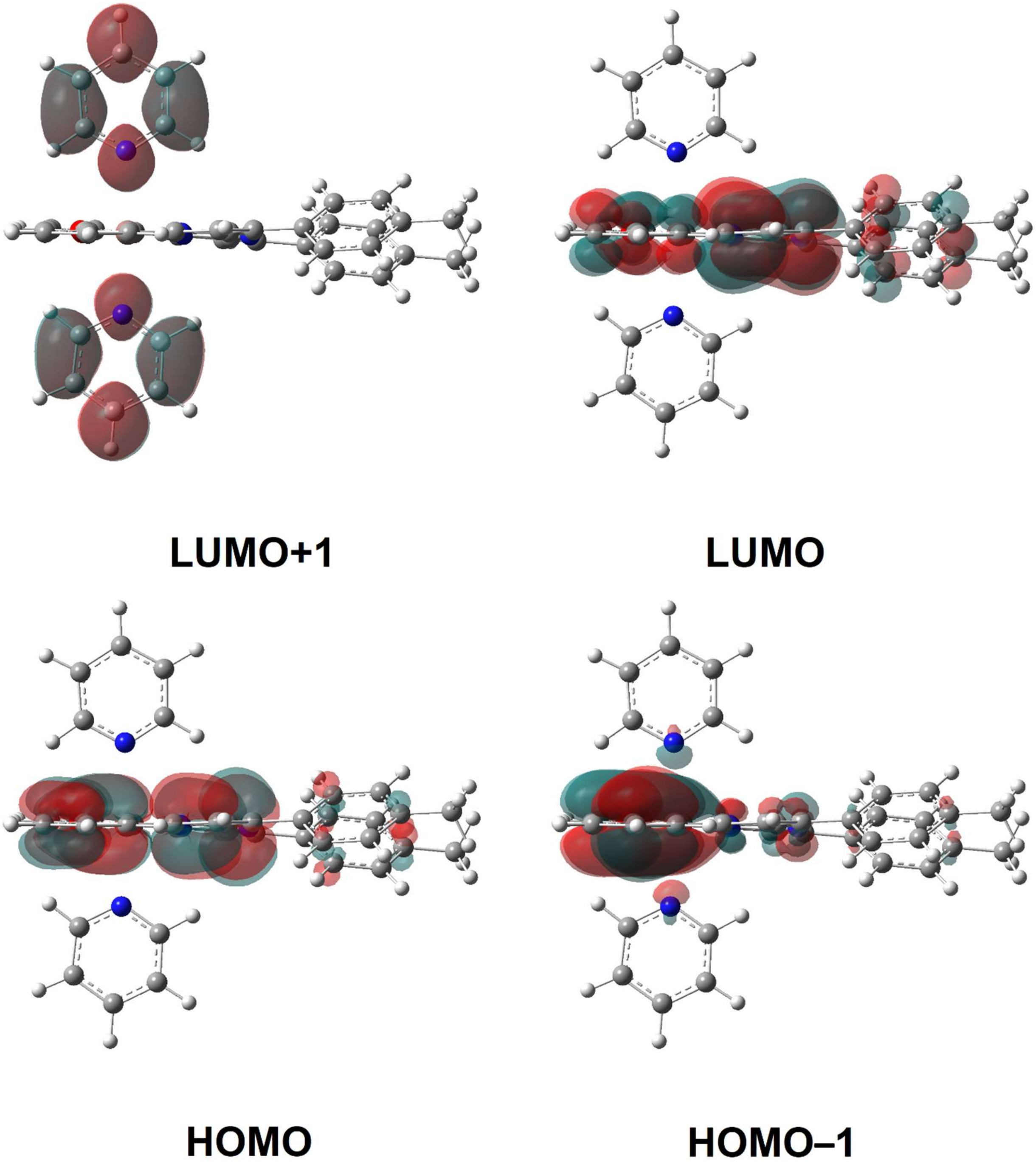

Figure S14. Horizontal view of Frontier Molecular Orbital Plots of **4a**.

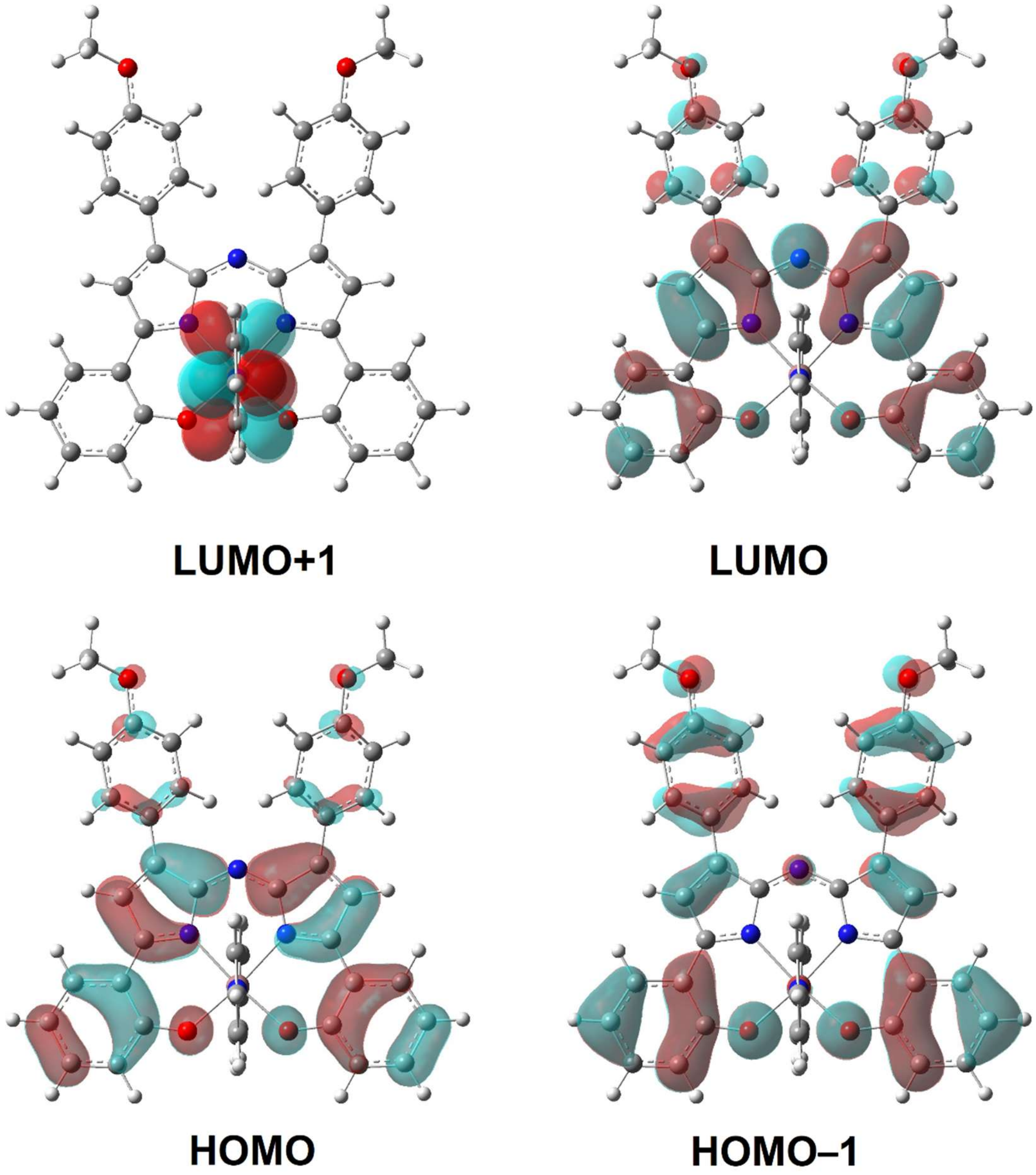

Figure S15. Frontier Molecular Orbital Plots of **4b**.

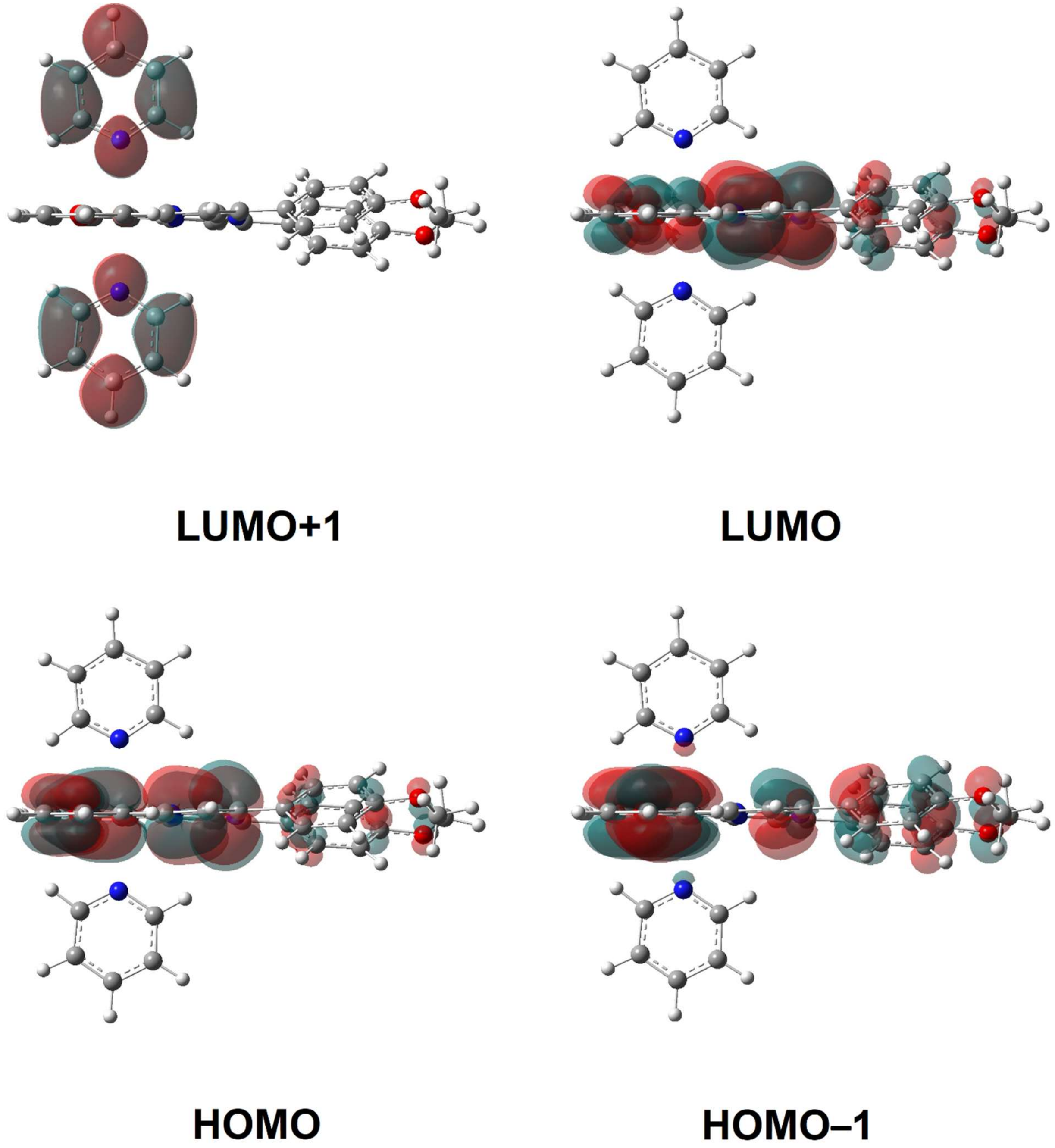

Figure S16. Horizontal view of Frontier Molecular Orbital Plots of **4b**.

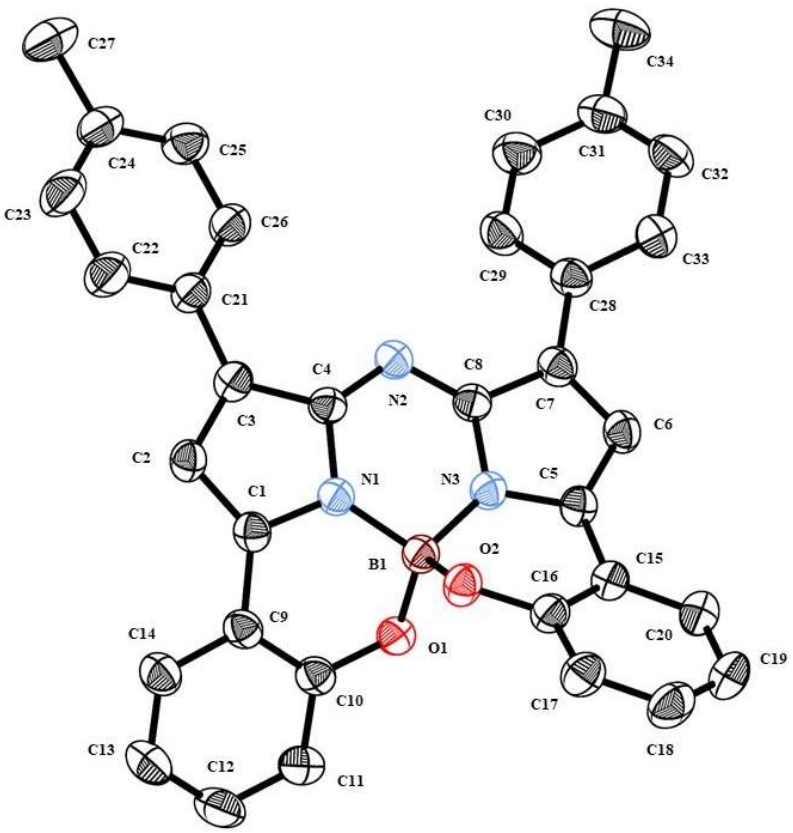

Figure S17. Single crystal X-ray diffraction structure of **1a**; ellipsoids are drawn at the 40% probability level. Hydrogen atoms omitted for clarity.

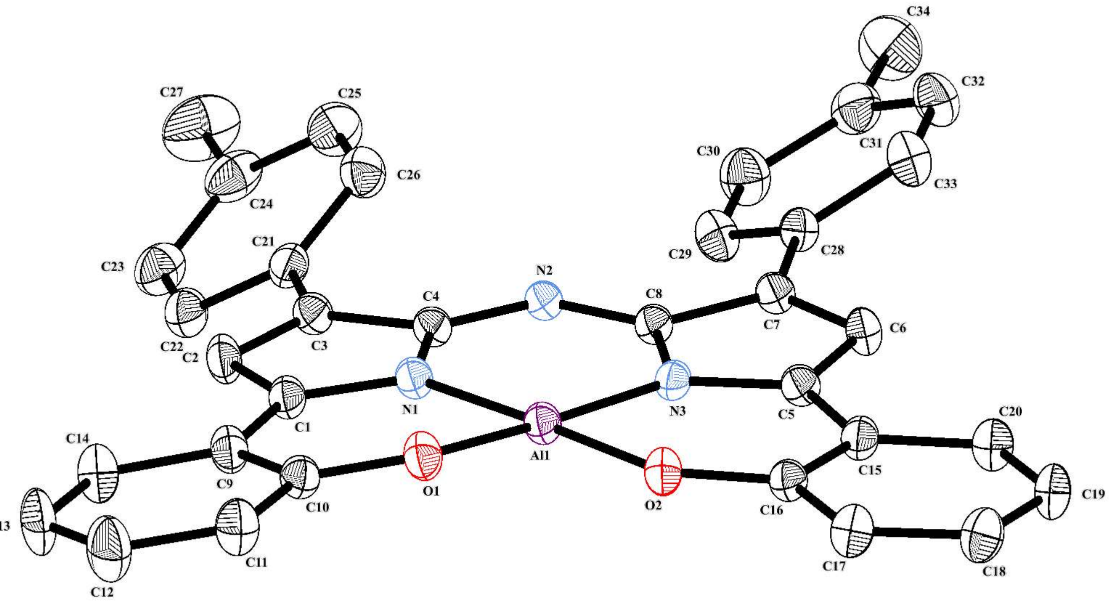

Figure S18. Single crystal X-ray diffraction structure of **2a**; ellipsoids are drawn at the 40% probability level. Axial bound pyridine ligands and hydrogen atoms omitted for clarity.

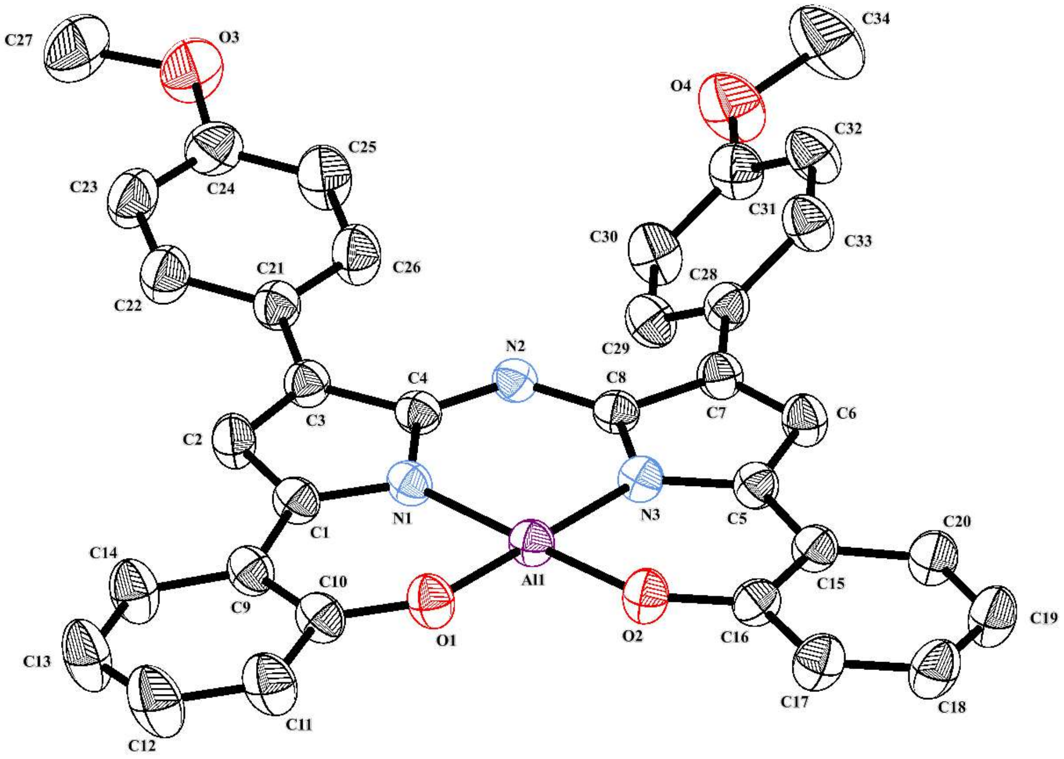

Figure S19. Single crystal X-ray diffraction structure of **2b**; ellipsoids are drawn at the 40% probability level. Axial bound pyridine ligands and hydrogen atoms omitted for clarity.

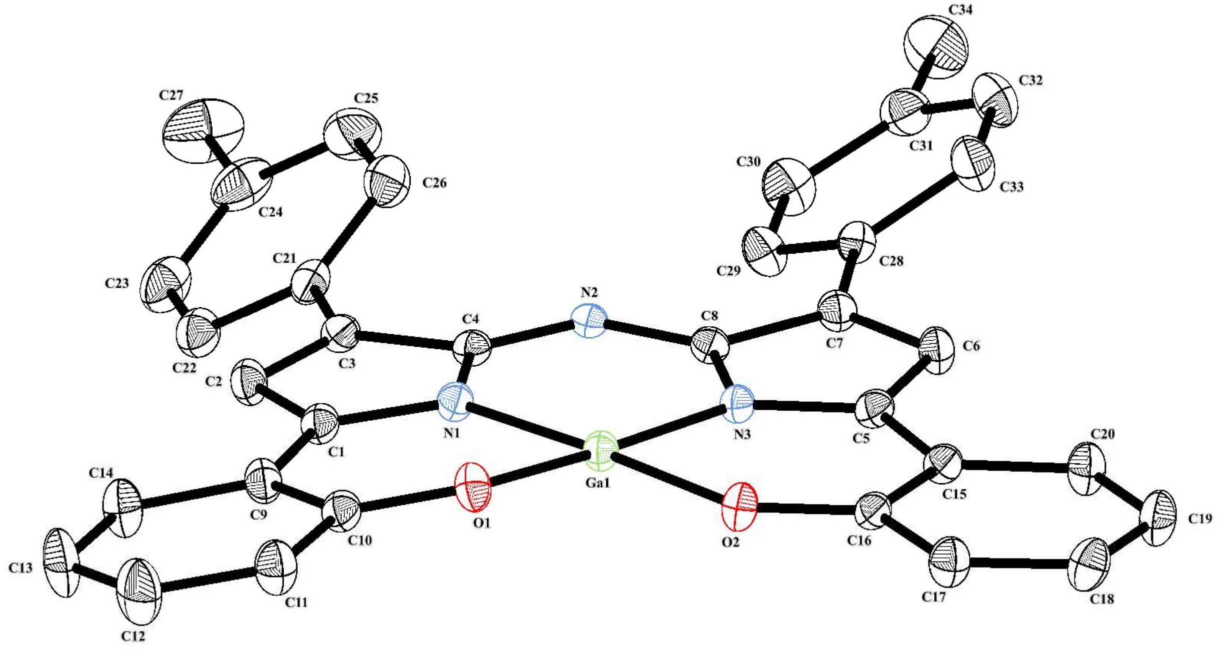

Figure S20. Single crystal X-ray diffraction structure of **3a**; ellipsoids are drawn at the 40% probability level. Axial bound pyridine ligands and hydrogen atoms omitted for clarity.

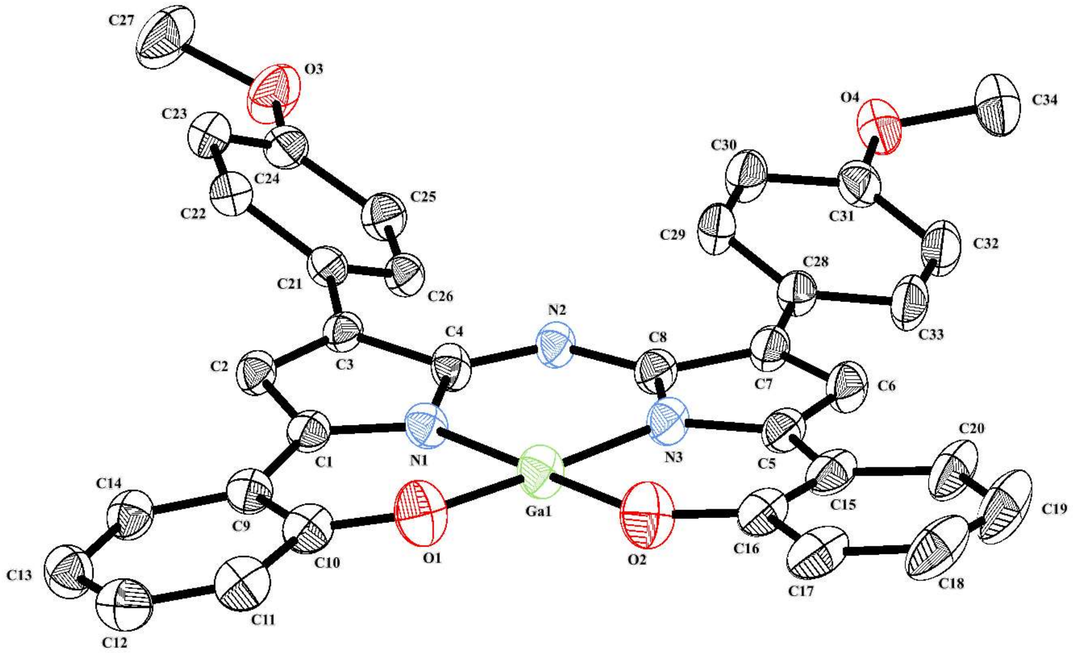

Figure S21. Single crystal X-ray diffraction structure of **3b**; ellipsoids are drawn at the 40% probability level. Axial bound pyridine ligands and hydrogen atoms omitted for clarity.

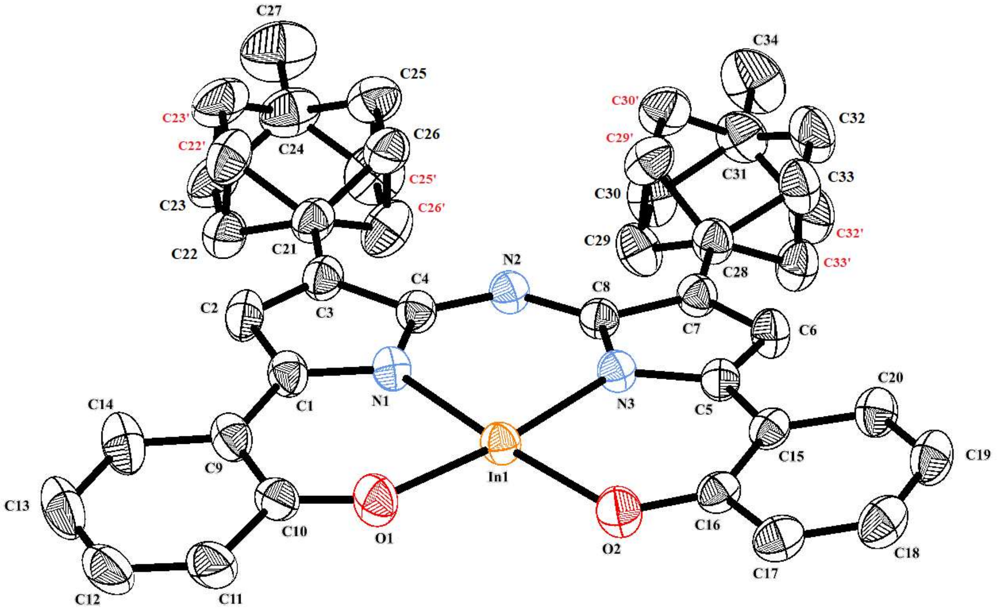

Figure S22. Single crystal X-ray diffraction structure of **4a**; ellipsoids are drawn at the 40% probability level. Axial bound pyridine ligands and hydrogen atoms omitted for clarity. Disordered carbon atoms on distal arenes are numbered in red.

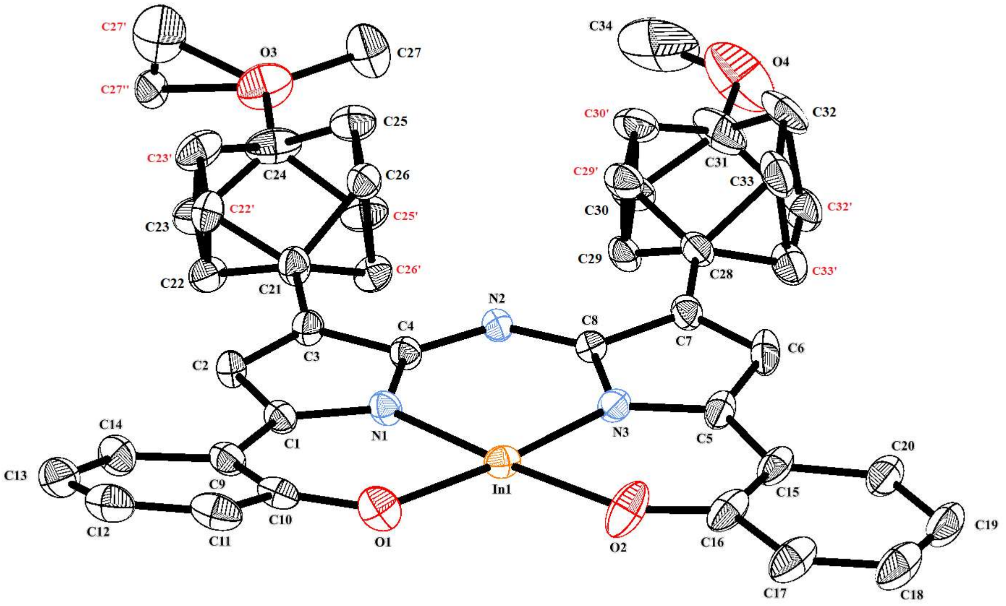

Figure S23. Single crystal X-ray diffraction structure of **4b**; ellipsoids are drawn at the 40% probability level. Axial bound pyridine ligands and hydrogen atoms omitted for clarity. Disordered carbon atoms on distal arenes are numbered in red.

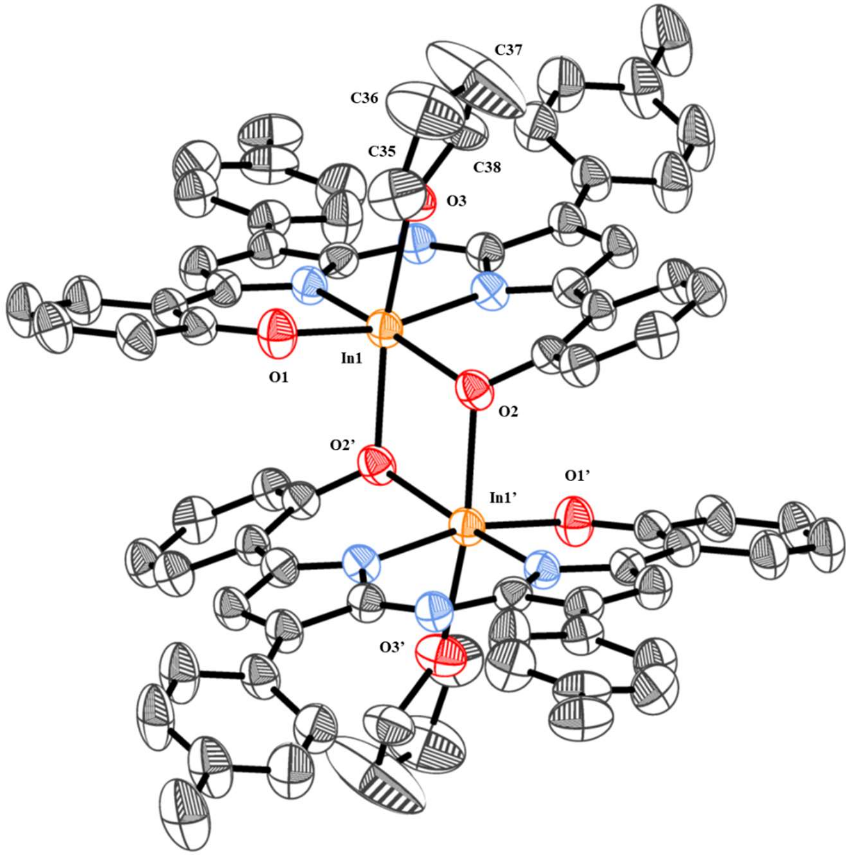

Figure S24. Single crystal X-ray diffraction structure of **4a'**; ellipsoids are drawn at the 40% probability level. Axial bound tetrahydrofuran ligands and hydrogen atoms omitted for clarity.

Description	1a (B-Me)	2a (Al-Me)	2b (Al-OMe)	3a (Ga-Me)
Empirical Formula	$C_{34}H_{24}N_3O_2B$	$C_{44}H_{34}N_5O_2Al$	$C_{44}H_{34}N_5O_4Al$	$C_{44}H_{34}N_5O_2Ga$
Formula Weight	517.37	691.74	723.74	734.48
Wavelength (Å)	0.71073	0.71073	0.71073	0.71073
Temperature (K)	293(2)	150(2)	293(2)	150(2)
Crystal system	Monoclinic	Triclinic	Triclinic	Triclinic
Space Group	*C* 2/*c*	*P*-1	*P*-1	*P*-1
a (Å)	17.8773(13)	9.5375(13)	9.4422(3)	9.5457(2)
b (Å)	17.6426(12)	13.824(2)	13.9608(4)	13.7422(3)
c (Å)	16.5701(12)	16.997(2)	16.9362(5)	17.0769(4)
α (°)	90	71.126(2)	105.914(2)	71.253(2)
β (°)	91.625(5)	89.184(2)	100.571(2)	89.086(2)
γ (°)	90	69.963(2)	106.290(2)	70.313(2)
V (Å^3)	5224.1(6)	1980.2(5)	1977.11(11)	1986.55(8)
Z	8	2	2	2
Density (g/cm^3)	1.316	1.160	1.216	1.228
μ (mm^{-1})	0.082	0.093	0.100	0.734
F (000)	2160	724	756	760
ϴ min., max. (°)	1.622, 27.908	2.2865, 23.8961	1.619, 27.407	1.266, 27.515
Crystal size (mm)	0.117 x 0.145 x 0.227	0.09 x 0.13 x 0.22	0.12 x 0.13 x 0.28	0.08 x 0.08 x 0.30
Index ranges	-23 < h < 23;<br>-22 < k < 23;<br>-21 < l < 21	-12 < h < 12;<br>-17 < k < 17;<br>-22 < l < 22	-12 < h < 12;<br>-18 < k < 18;<br>-21 < l < 21	-12 < h < 12;<br>-17 < k < 17;<br>-22 < l < 22
Reflections collected	35641	19176	27131	26145
R_{int} (%)	0.0317	0.0432	0.0420	0.0382
Data/restraints/parameters	6163/0/364	9025/0/471	8924/0/489	9069/0/471
Goodness-of-fit on F^2	1.036	0.999	1.024	1.053
R_1 [I > 2σ(I)]	0.0441	0.0476	0.0485	0.0378
wR_2 [I > 2σ(I)]	0.1226	0.1245	0.1480	0.0930
Largest diff. peak/hole (e Å^{-3})	0.285 and -0.225	0.263 and -0.255	0.211 and -0.328	0.470 and -0.572
Observed data [I > 2.0σ(I)]	4163	5574	5045	7000

Table S3. Crystallographic data and refinement parameters for **1a**, **2a-b** and **3a-b.**

Description	3b (Ga-OMe)	4a (In-Me)	4b (In-OMe)	4a' (In-Me-Dimer)
Empirical Formula	$C_{44}H_{34}N_5O_4Ga$	$C_{44}H_{34}N_5O_2In$	$C_{44}H_{34}N_5O_4In$	$C_{76}H_{64}N_6O_6In_2$
Formula Weight	766.48	779.58	811.58	1386.97
Wavelength (Å)	0.71073	0.71073	0.71073	0.71073
Temperature (K)	215(2)	293(2)	150(2)	293(2)
Crystal system	Orthorhombic	Triclinic	Triclinic	Triclinic
Space Group	*Fdd*2	*P*-1	*P*-1	*P*-1
a (Å)	20.9391(10)	9.7402(16)	9.7573(4)	8.7943(8)
b (Å)	25.3066(13)	9.9876(16)	9.8180(4)	11.7579(10)
c (Å)	29.4364(15)	19.774(3)	19.3367(9)	14.8580(13)
α (°)	90	104.509(8)	103.7920(10)	98.225(6)
β (°)	90	100.981(8)	98.2980(10)	90.613(6)
γ (°)	90	96.196(9)	95.5750(10)	90.271(6)
V ($Å^3$)	15598.3(14)	1803.5(5)	1763.40(13)	1520.4(2)
Z	16	2	2	1
Density (g/cm^3)	1.306	1.436	1.528	1.515
μ (mm^{-1})	0.754	0.701	0.724	0.821
F (000)	6336	796	828	708
ϴ min., max. (°)	2.123, 27.526	2.111, 27.749	2.129, 27.489	1.750, 28.332
Crystal size (mm)	0.26 x 0.26 x 0.28	0.08 x 0.24 x 0.55	0.07 x 0.19 x 0.24	0.038 x 0.082 x 0.085
Index ranges	-27 < h < 27;<br>-32 < k < 32;<br>-38 < l < 38	-12 < h < 12;<br>-13 < k < 12;<br>-25 < l < 25	-14 < h < 14;<br>-15 < k < 15;<br>-29 < l < 29	-11 < h < 11;<br>-15 < k < 15;<br>-19 < l < 19
Reflections collected	36867	23828	17040	20853
R_{int} (%)	0.0494	0.0247	0.0266	0.0781
Data/restraints/parameters	8973/1/490	8295/0/544	8022/1/631	7503/0/409
Goodness-of-fit on F^2	1.020	1.038	1.032	0.934
R_1 [I > 2σ(I)]	0.0359	0.0268	0.0295	0.0529
wR_2 [I > 2σ(I)]	0.0816	0.0721	0.0732	0.1379
Largest diff. peak/hole (e $Å^{-3}$)	0.215 and -0.304	0.520 and -0.297	0.700 and -0.605	0.763 and -0.865
Observed data [I > 2.0σ(I)]	7160	7473	7290	4065

Table S4. Crystallographic data and refinement parameters for **3b**, **4a-b** and **4a'**.

Compound	Planarity of M Center (°)				
	N1-M-N3	N1-M-O1	O1-M-O2	N3-M-O2	Planarity vs 360°
1a (B-Me)	103.41(11)	106.91(11)	107.20(11)	106.18(11)	63.70
2a (Al-Me)	88.44(6)	91.70(6)	89.23(6)	90.74(6)	0.13
2b (Al-OMe)	88.72(7)	91.16(7)	89.45(7)	90.66(7)	0.02
3a (Ga-Me)	88.97(6)	91.89(6)	88.10(6)	91.18(6)	0.16
3b (Ga-OMe)	88.71(11)	92.01(12)	86.65(12)	92.59(12)	-0.04
4a (In-Me)	85.47(6)	87.32(6)	99.75(6)	87.53(6)	0.09
4b (In-OMe)	85.84(6)	86.75(6)	99.81(7)	87.60(7)	0.03
4a' (In-Me-Dimer)	86.64(3)	87.73(5)	102.66(4)	85.20(1)	2.24

Table S5. Crystallographic structure bond angles of metal center in **1a**, **2a-b, 3a-b, 4a-b**, and **4a'**.

Compound	Axial Pyridine Ligands (°)	Proximal Dihedral Angles (°)		Phenolic Puckering (°)
	N4-M-N5	C2-C1-C9-C14	C6-C5-C15-C20	O1-N1-N3-O2
1a (B-Me)	--	-22.23(9)	-24.41(4)	-66.02(5)
2a (Al-Me)	176.55(7)	4.44(5)	-3.62(1)	3.31(8)
2b (Al-OMe)	179.07(7)	5.20(1)	-5.11(7)	-0.23(4)
3a (Ga-Me)	175.39(6)	4.35(5)	-3.32(8)	3.84(1)
3b (Ga-OMe)	175.3(3)	12.18(2)	0.51(1)	1.31(8)
4a (In-Me)	171.50(9)	1.34(1)	12.69(9)	2.89(6)
4b (In-OMe)	170.26(8)	2.17(4)	-8.81(5)	0.88(3)
4a' (In-Me-Dimer)	163.81(12)*	-5.23(9)	6.05(6)	17.44(3)

*Axial THF ligands measured through O3(THF)-In1-O2'.

Table S6. Crystallographic structure axial ligand angles and dihedral angles of phenolic puckering in **1a**, **2a-b, 3a-b, 4a-b**, and **4a'**.

References

1. G. Zhang, C. B. Musgrave, *J. Phys. Chem. A*, 2007, **111**, 1554-1561.
2. P. Ziegler, *Bruker TopSpin 3.2 User Manual,* 2013, 112-131.

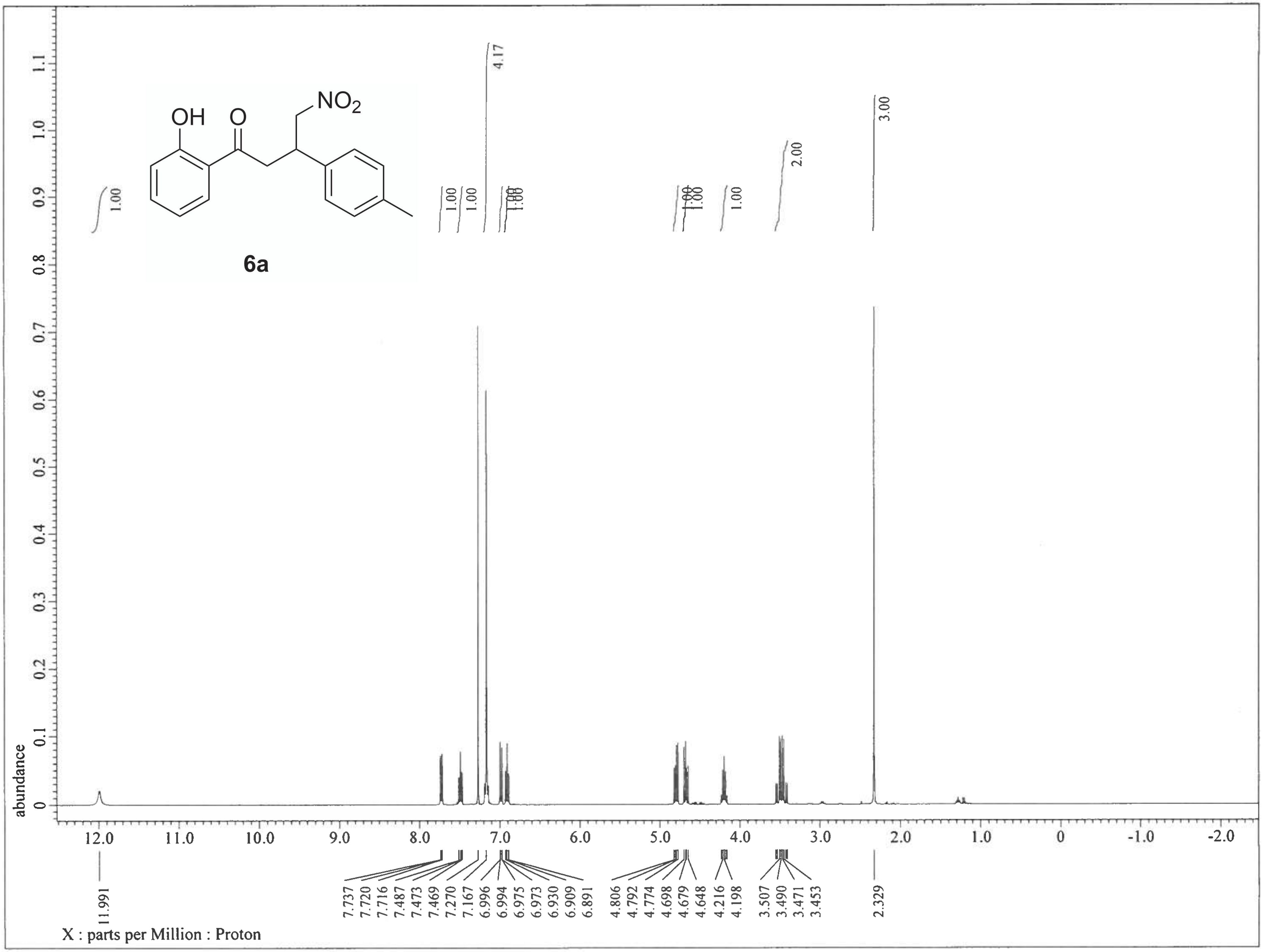

OH
O
NO2
6a
1.00
1.00
1.00
4.17
1.00
1.00
1.00
1.00
1.00
2.00
3.00
12.0
11.0
10.0
9.0
8.0
7.0
6.0
5.0
4.0
3.0
2.0
1.0
0
-1.0
-2.0
abundance
11.991
7.737
7.720
7.716
7.487
7.473
7.469
7.270
7.167
6.996
6.994
6.975
6.973
6.930
6.909
6.891
4.806
4.792
4.774
4.698
4.679
4.648
4.216
4.198
3.507
3.490
3.471
3.453
2.329
X : parts per Million : Proton

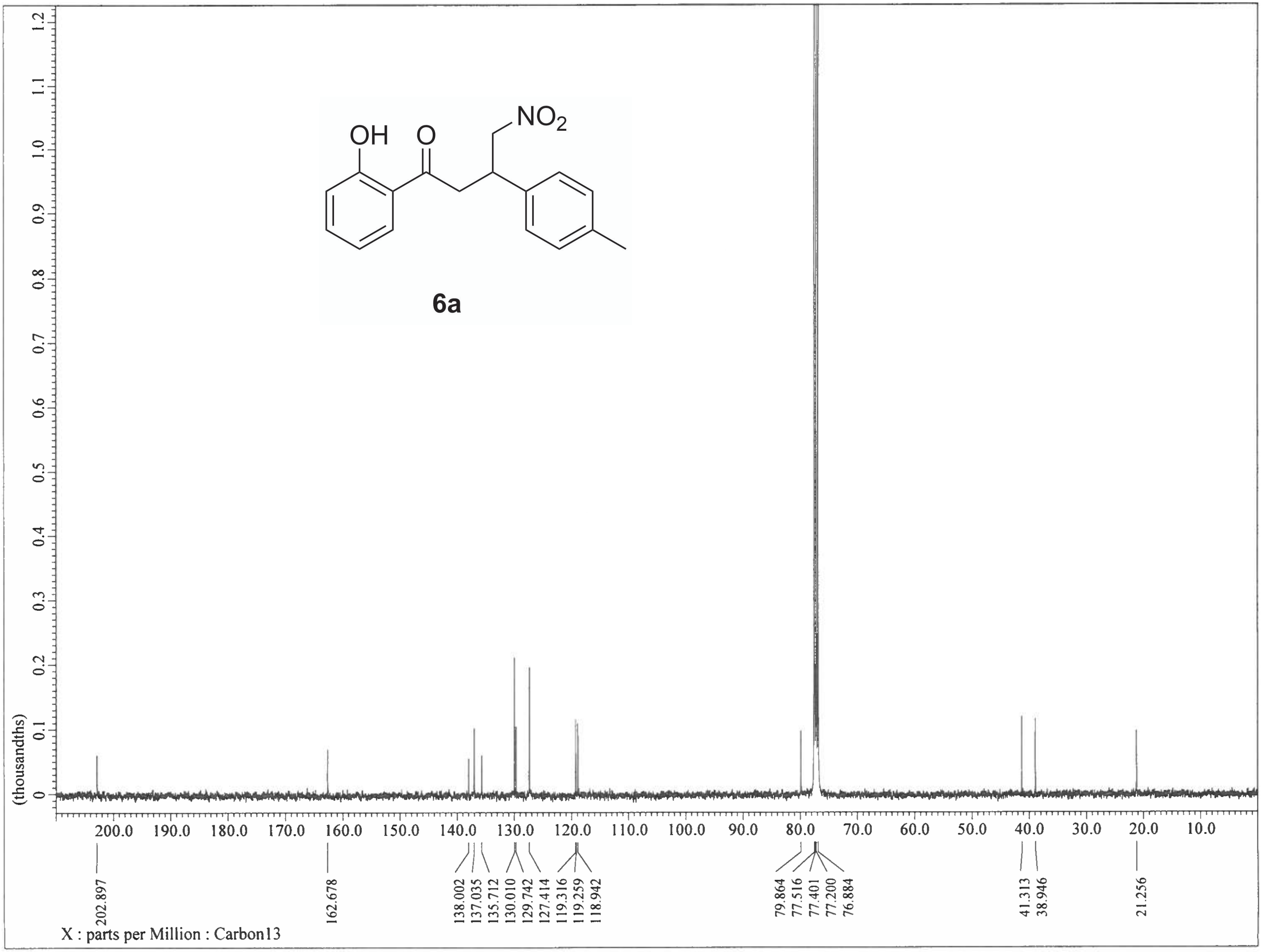

OH
O
NO2
6a
(thousandths)
0
0.1
0.2
0.3
0.4
0.5
0.6
0.7
0.8
0.9
1.0
1.1
1.2
200.0
190.0
180.0
170.0
160.0
150.0
140.0
130.0
120.0
110.0
100.0
90.0
80.0
70.0
60.0
50.0
40.0
30.0
20.0
10.0
202.897
162.678
138.002
137.035
135.712
130.010
129.742
127.414
119.316
119.259
118.942
79.864
77.516
77.401
77.200
76.884
41.313
38.946
21.256
X : parts per Million : Carbon13

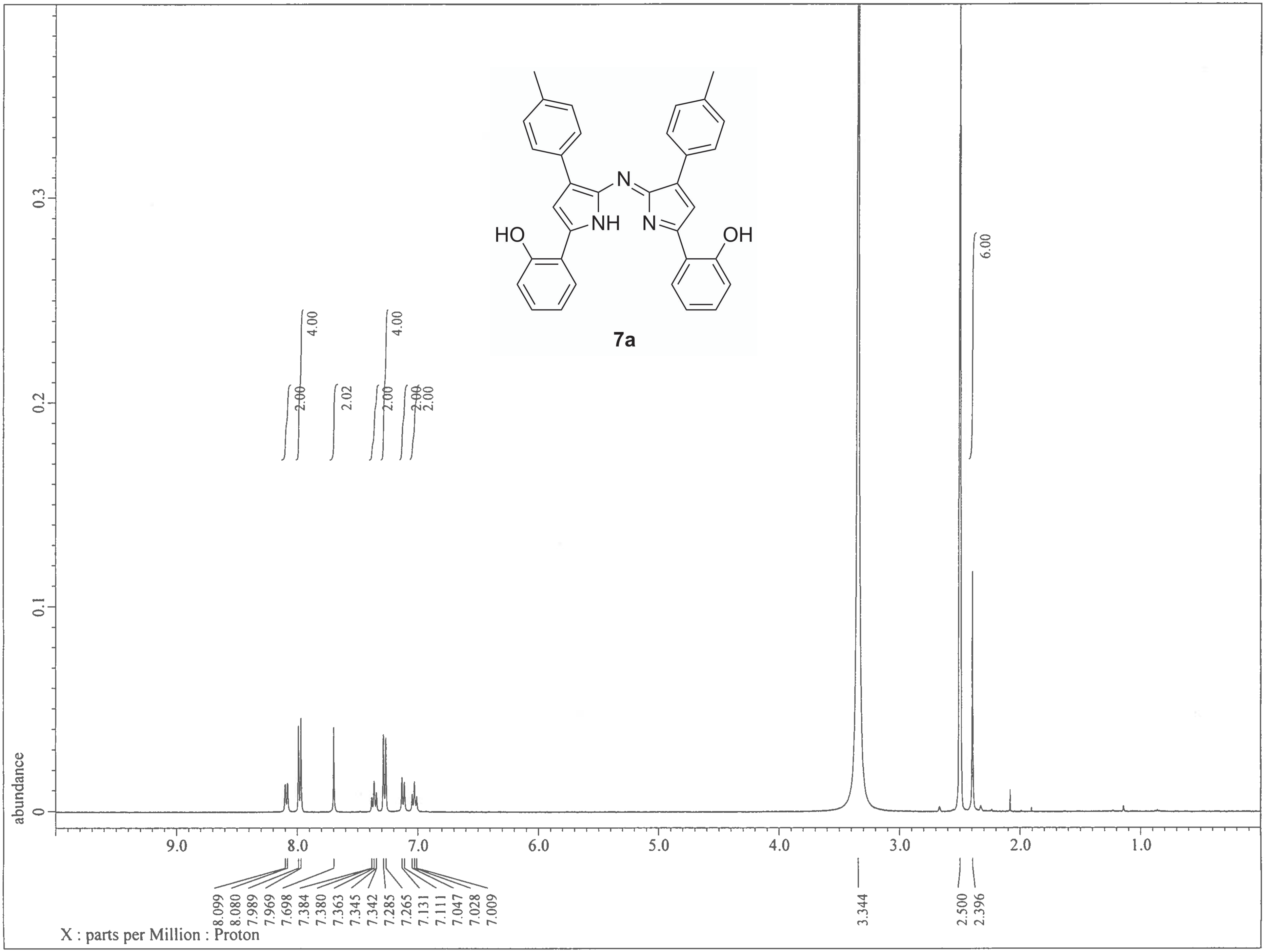
7a
HO
NH
N
N
OH
abundance
0
0.1
0.2
0.3
2.00
4.00
2.02
2.00
4.00
2.00
2.00
6.00
9.0
8.0
7.0
6.0
5.0
4.0
3.0
2.0
1.0
8.099
8.080
7.989
7.969
7.698
7.384
7.380
7.363
7.345
7.342
7.285
7.265
7.131
7.111
7.047
7.028
7.009
3.344
2.500
2.396
X : parts per Million : Proton

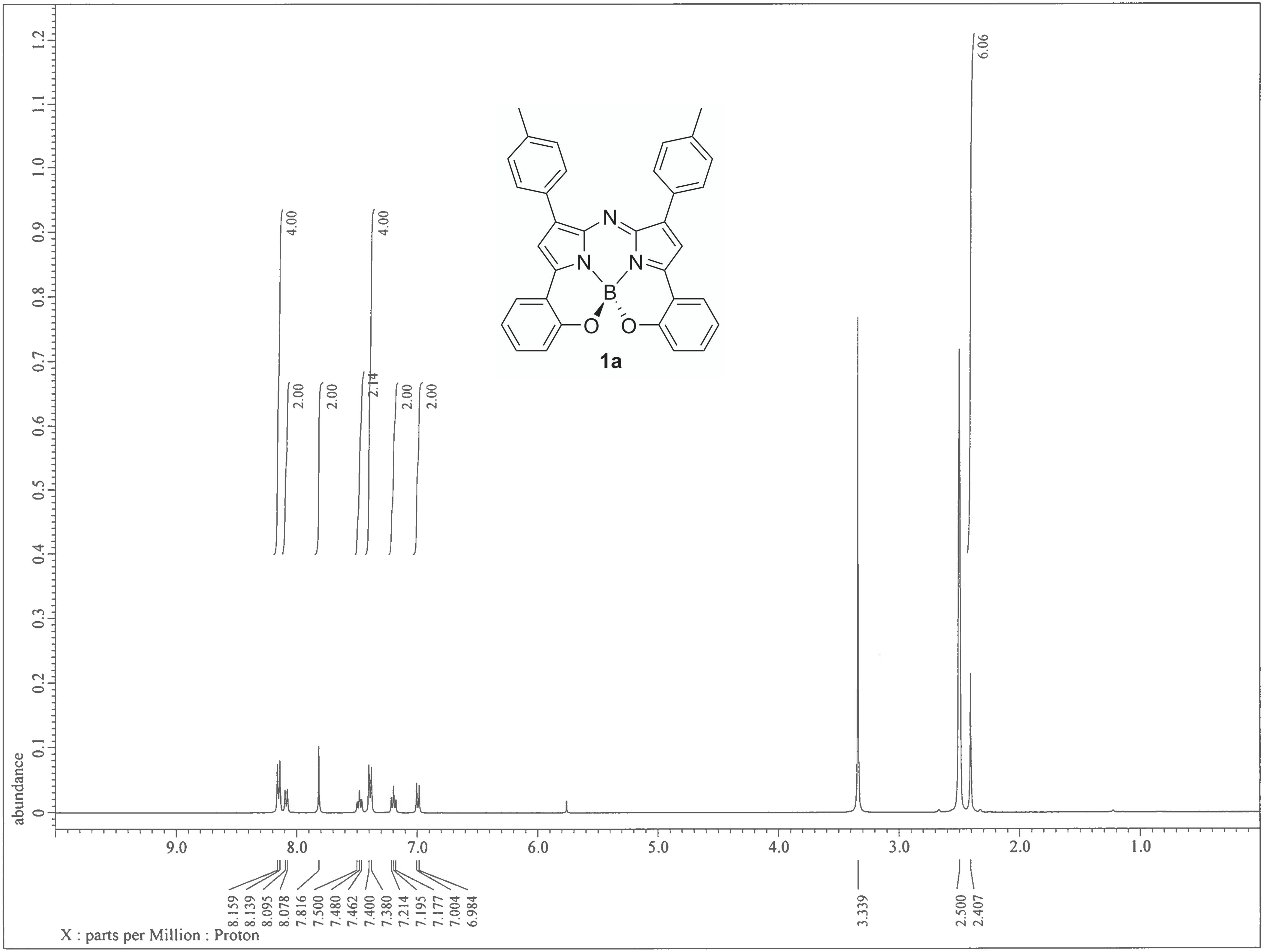
1a
abundance
X : parts per Million : Proton
4.00
2.00
2.00
4.00
2.14
2.00
2.00
6.06
8.159
8.139
8.095
8.078
7.816
7.500
7.480
7.462
7.400
7.380
7.214
7.195
7.177
7.004
6.984
3.339
2.500
2.407

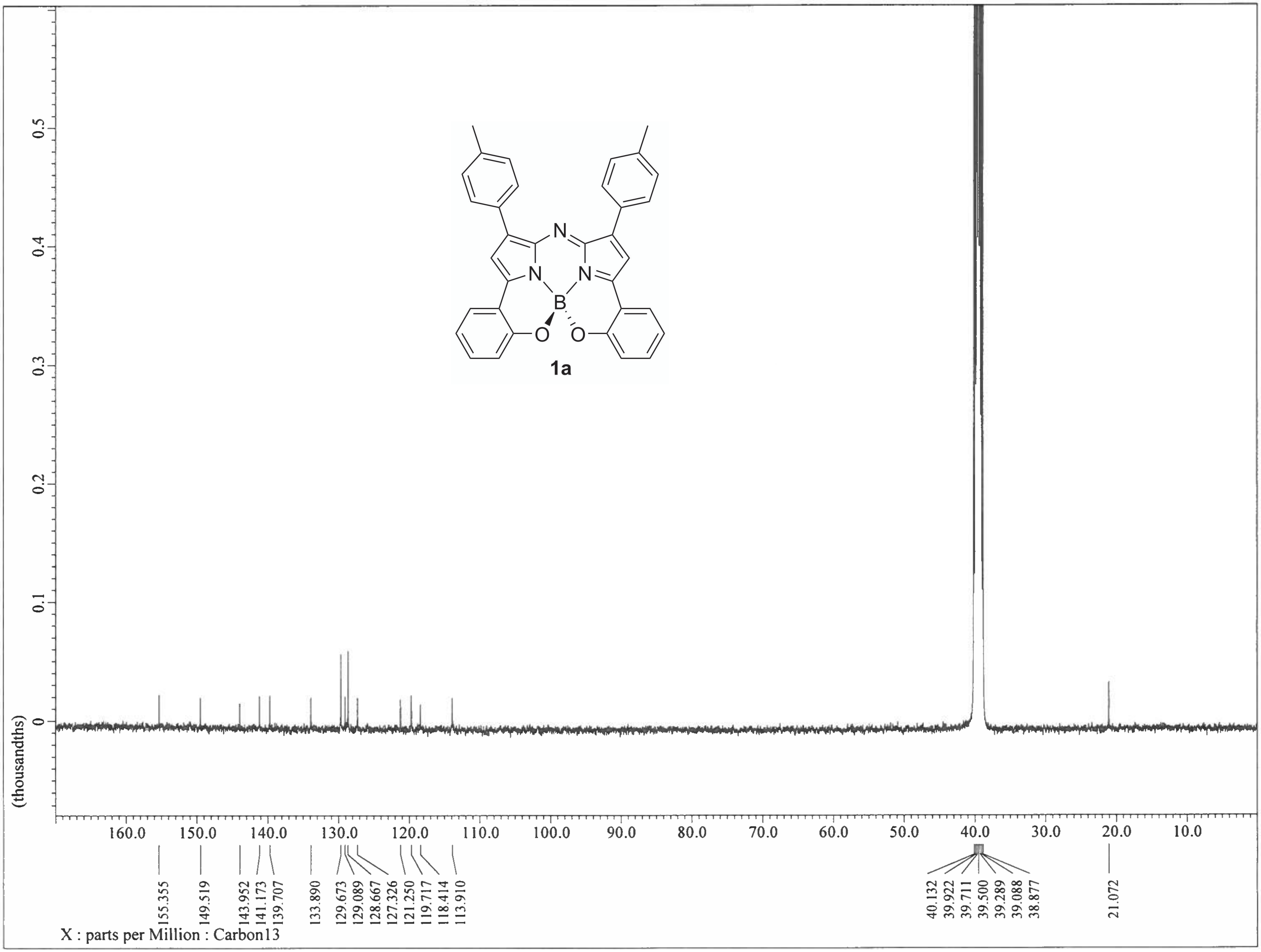
1a
0.5
0.4
0.3
0.2
0.1
0
(thousandths)
160.0 150.0 140.0 130.0 120.0 110.0 100.0 90.0 80.0 70.0 60.0 50.0 40.0 30.0 20.0 10.0
155.355
149.519
143.952
141.173
139.707
133.890
129.673
129.089
128.667
127.326
121.250
119.717
118.414
113.910
40.132
39.922
39.711
39.500
39.289
39.088
38.877
21.072
X : parts per Million : Carbon13

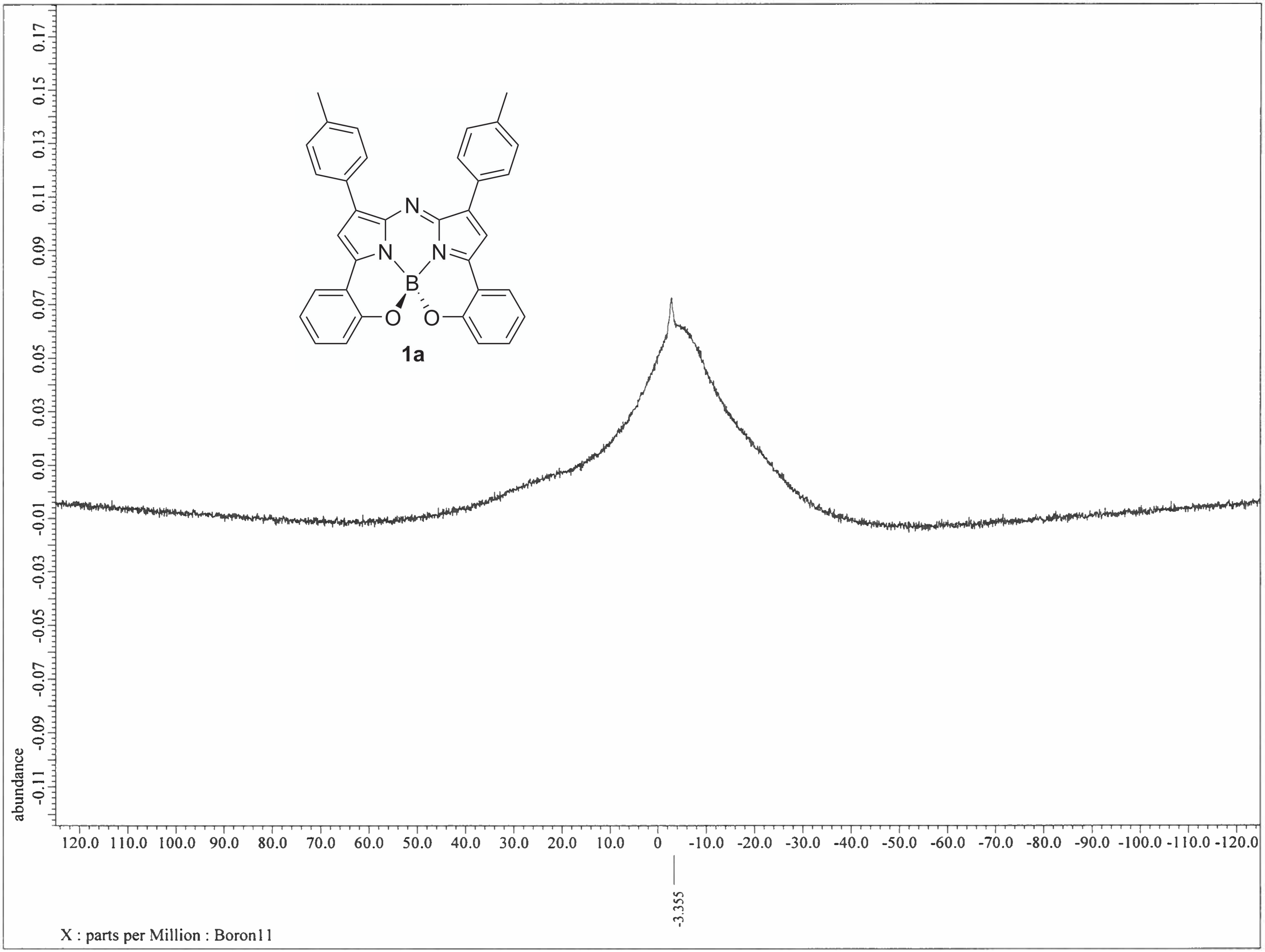
1a
N
B
O
abundance
0.17
0.15
0.13
0.11
0.09
0.07
0.05
0.03
0.01
-0.01
-0.03
-0.05
-0.07
-0.09
-0.11
120.0 110.0 100.0 90.0 80.0 70.0 60.0 50.0 40.0 30.0 20.0 10.0 0 -10.0 -20.0 -30.0 -40.0 -50.0 -60.0 -70.0 -80.0 -90.0 -100.0 -110.0 -120.0
-3.355
X : parts per Million : Boron11

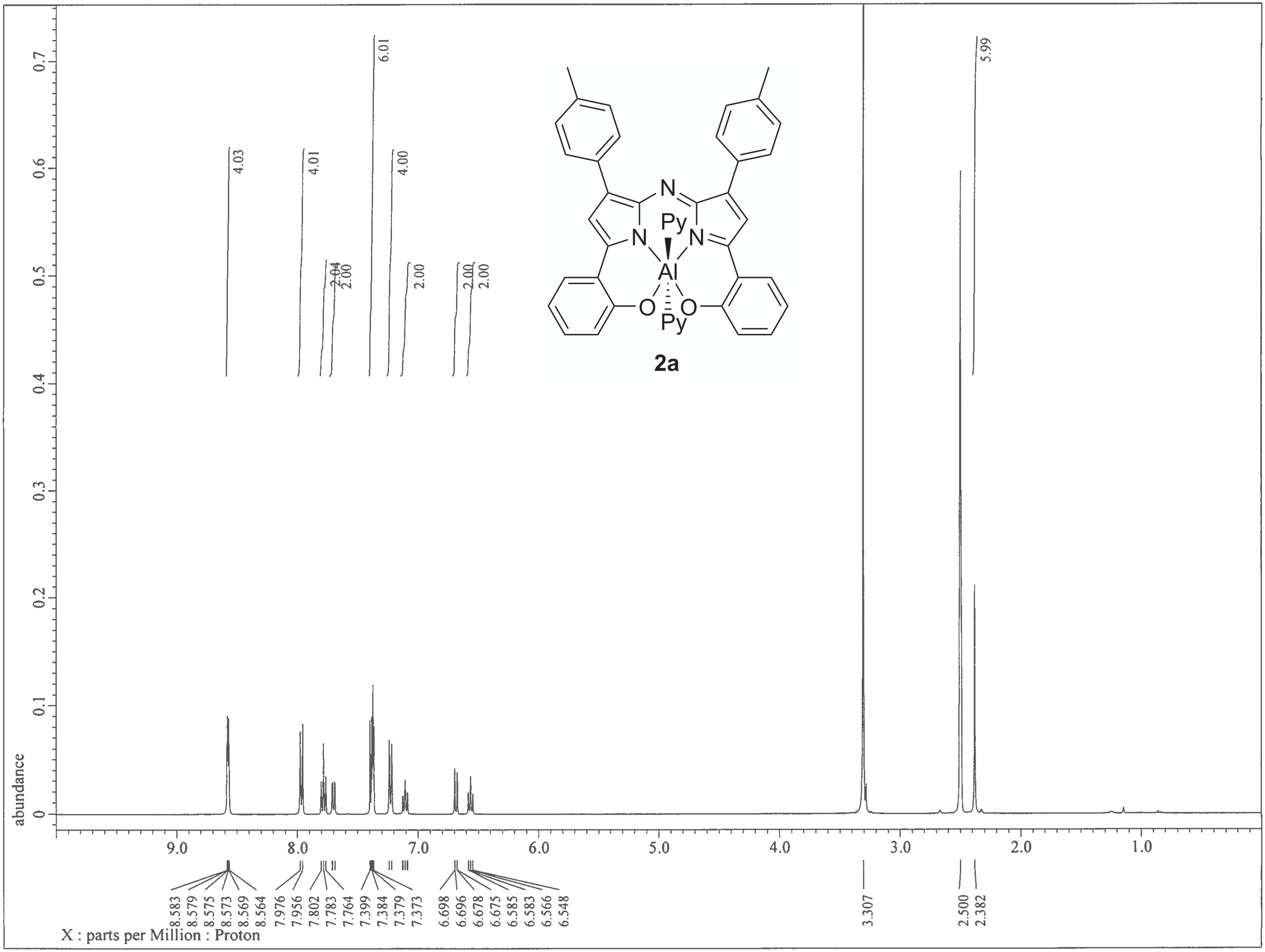

2a
Py
Al
N
O
abundance
0
0.1
0.2
0.3
0.4
0.5
0.6
0.7
9.0
8.0
7.0
6.0
5.0
4.0
3.0
2.0
1.0
X : parts per Million : Proton
4.03
4.01
2.04
2.00
6.01
4.00
2.00
2.00
2.00
5.99
8.583
8.579
8.575
8.573
8.569
8.564
7.976
7.956
7.802
7.783
7.764
7.399
7.384
7.379
7.373
6.698
6.696
6.678
6.675
6.585
6.583
6.566
6.548
3.307
2.500
2.382

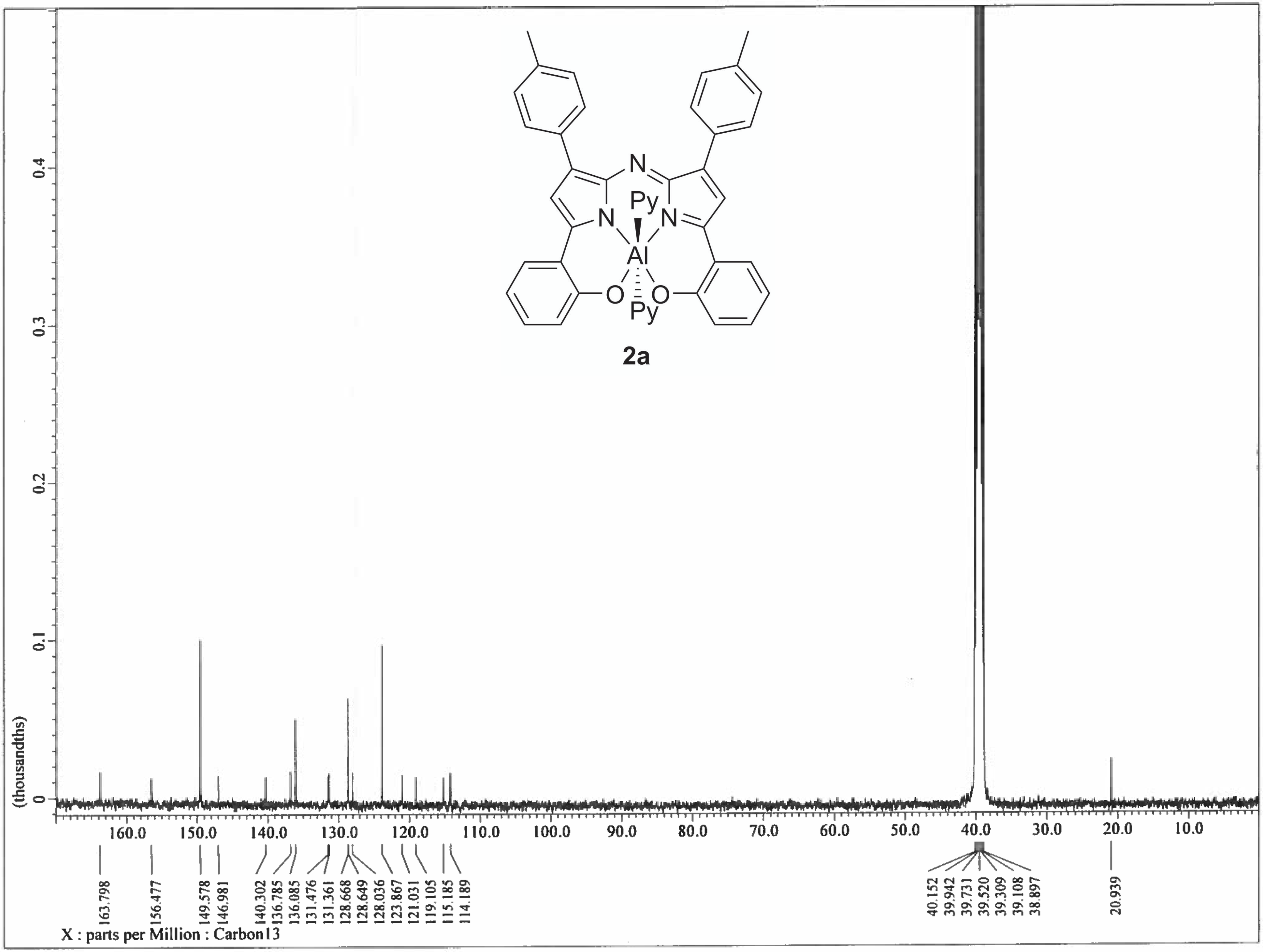

Py
N
N
N
Al
O
O
Py
2a
(thousandths)
0.4
0.3
0.2
0.1
0
160.0
150.0
140.0
130.0
120.0
110.0
100.0
90.0
80.0
70.0
60.0
50.0
40.0
30.0
20.0
10.0
163.798
156.477
149.578
146.981
140.302
136.785
136.085
131.476
131.361
128.668
128.649
128.036
123.867
121.031
119.105
115.185
114.189
40.152
39.942
39.731
39.520
39.309
39.108
38.897
20.939
X : parts per Million : Carbon13

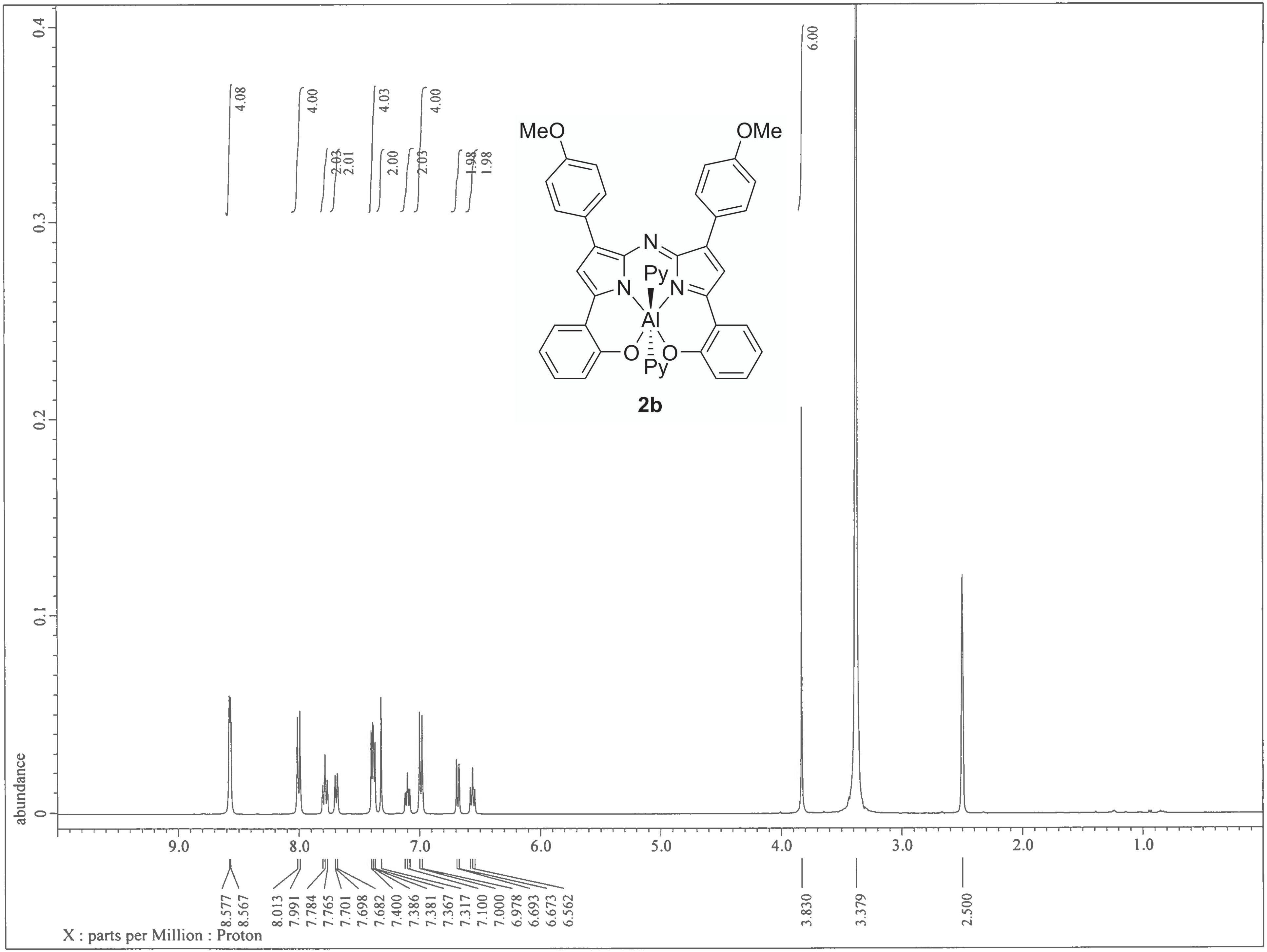

MeO
OMe
N
Py
N
N
Al
O
O
Py
2b
4.08
4.00
2.03
2.01
4.03
2.00
2.03
4.00
1.98
1.98
6.00
0.4
0.3
0.2
0.1
0
abundance
9.0
8.0
7.0
6.0
5.0
4.0
3.0
2.0
1.0
8.577
8.567
8.013
7.991
7.784
7.765
7.701
7.698
7.682
7.400
7.386
7.381
7.367
7.317
7.100
7.000
6.978
6.693
6.673
6.562
3.830
3.379
2.500
X : parts per Million : Proton

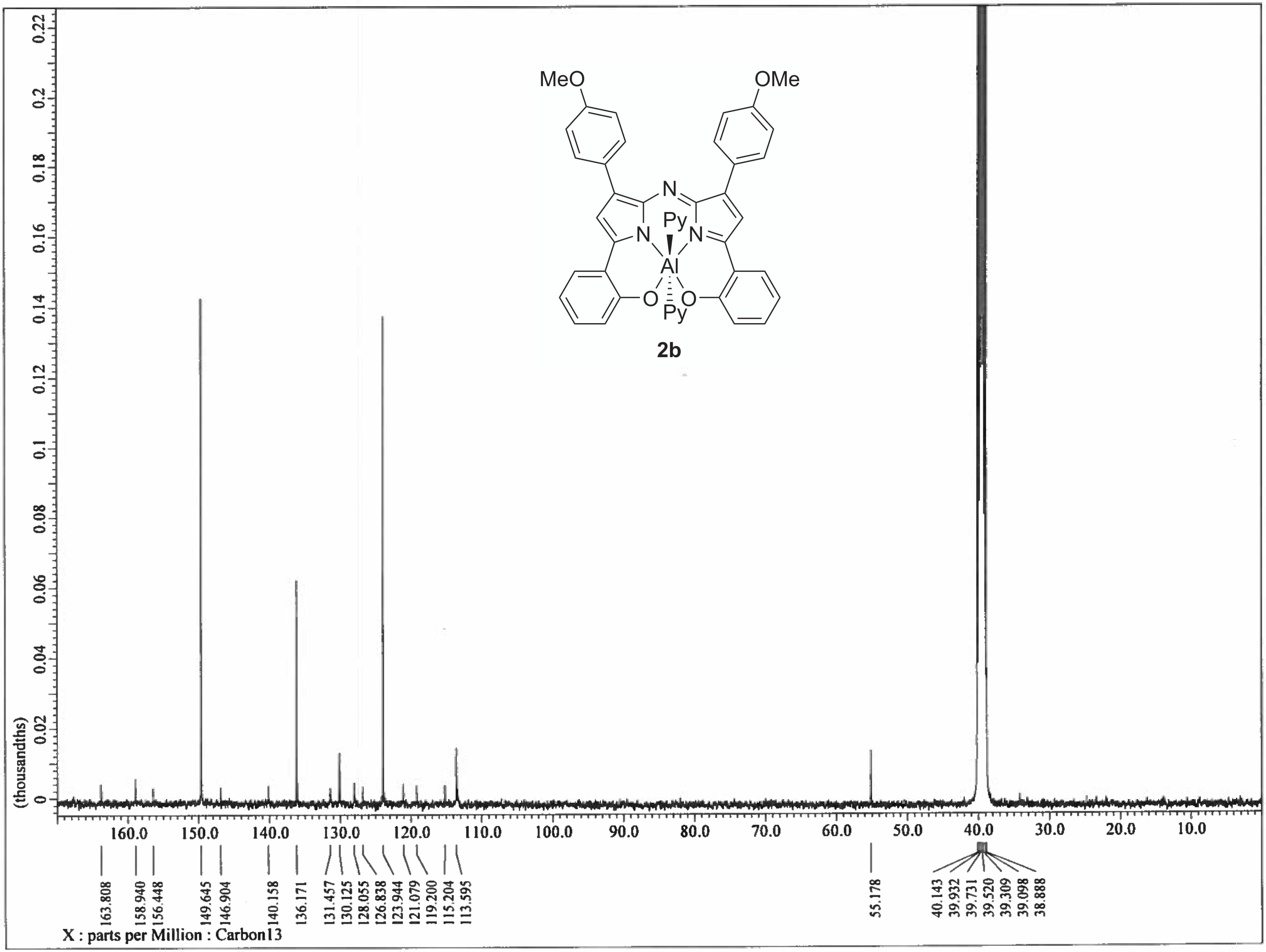
MeO
OMe
N
Py
N
N
Al
O
O
Py
2b
(thousandths)
0
0.02
0.04
0.06
0.08
0.1
0.12
0.14
0.16
0.18
0.2
0.22
160.0
150.0
140.0
130.0
120.0
110.0
100.0
90.0
80.0
70.0
60.0
50.0
40.0
30.0
20.0
10.0
163.808
158.940
156.448
149.645
146.904
140.158
136.171
131.457
130.125
128.055
126.838
123.944
121.079
119.200
115.204
113.595
55.178
40.143
39.932
39.731
39.520
39.309
39.098
38.888
X : parts per Million : Carbon13

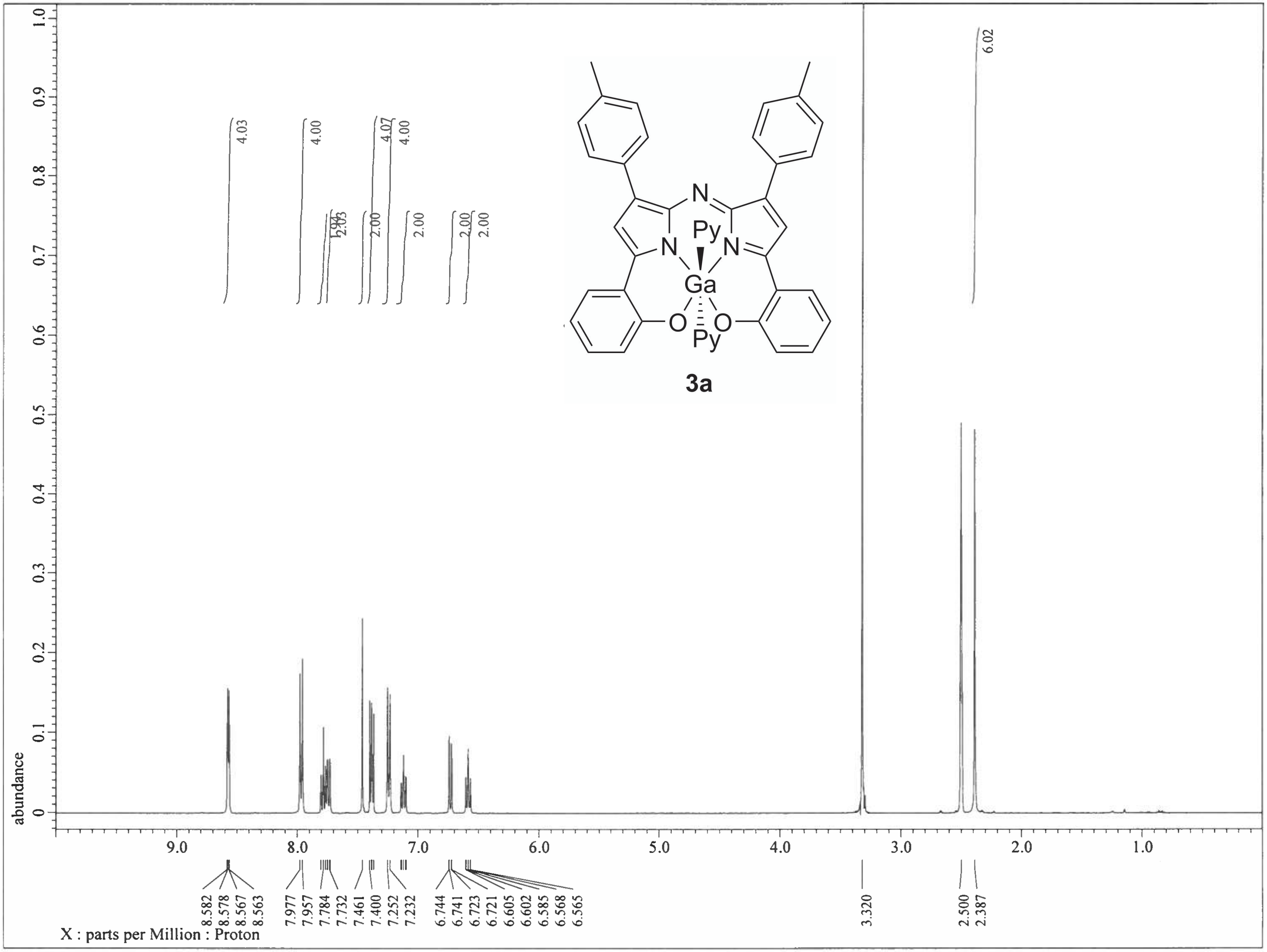

3a
Py
N
Ga
O
abundance
X : parts per Million : Proton
9.0
8.0
7.0
6.0
5.0
4.0
3.0
2.0
1.0
4.03
4.00
4.07
4.00
2.00
2.00
2.00
2.00
6.02
8.582
8.578
8.567
8.563
7.977
7.957
7.784
7.732
7.461
7.400
7.252
7.232
6.744
6.741
6.723
6.721
6.605
6.602
6.585
6.568
6.565
3.320
2.500
2.387

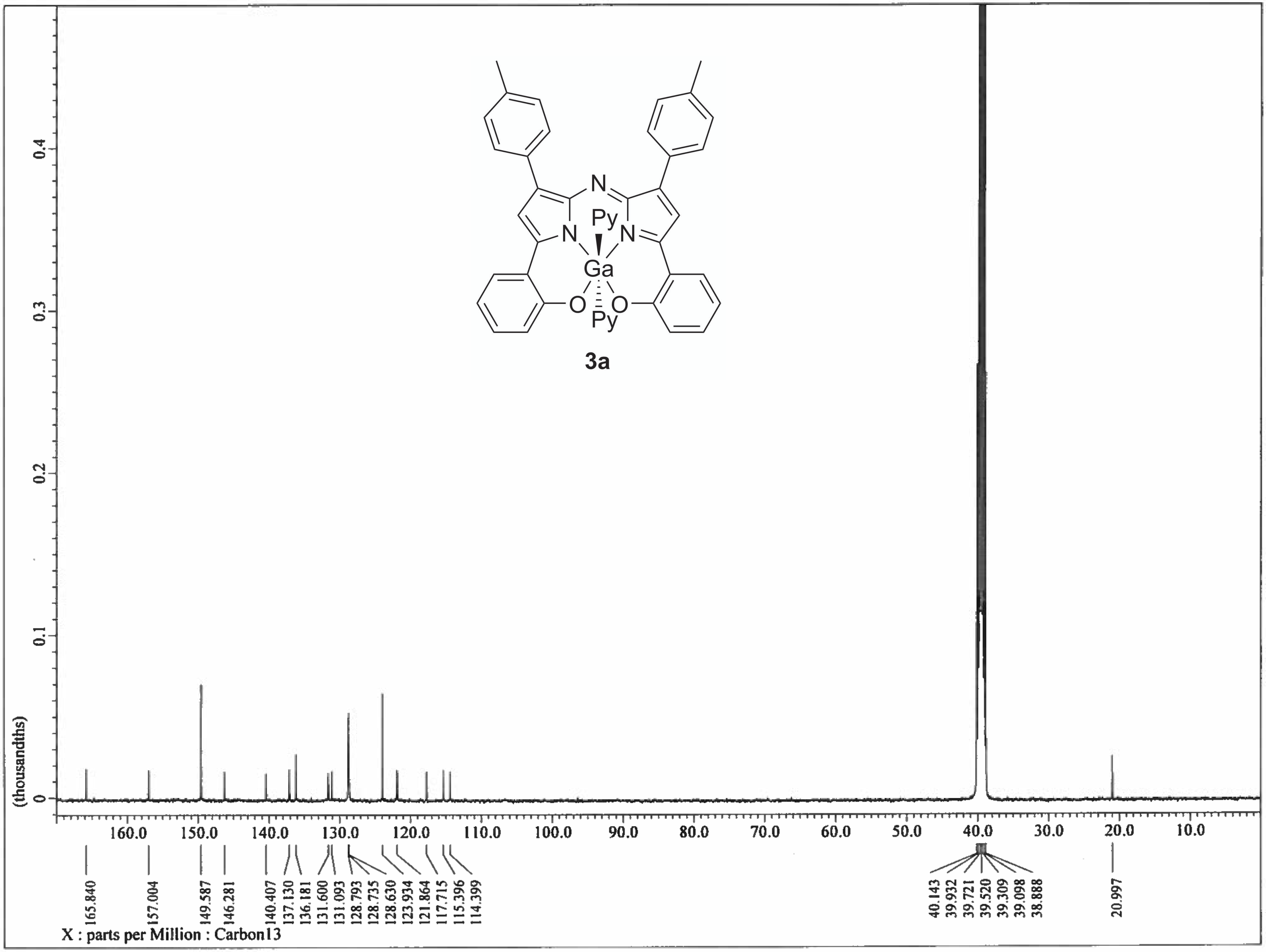

N
Py
N
N
Ga
O
O
Py
3a
0.4
0.3
0.2
0.1
0
(thousandths)
160.0
150.0
140.0
130.0
120.0
110.0
100.0
90.0
80.0
70.0
60.0
50.0
40.0
30.0
20.0
10.0
165.840
157.004
149.587
146.281
140.407
137.130
136.181
131.600
131.093
128.793
128.735
128.630
123.934
121.864
117.715
115.396
114.399
40.143
39.932
39.721
39.520
39.309
39.098
38.888
20.997
X : parts per Million : Carbon13

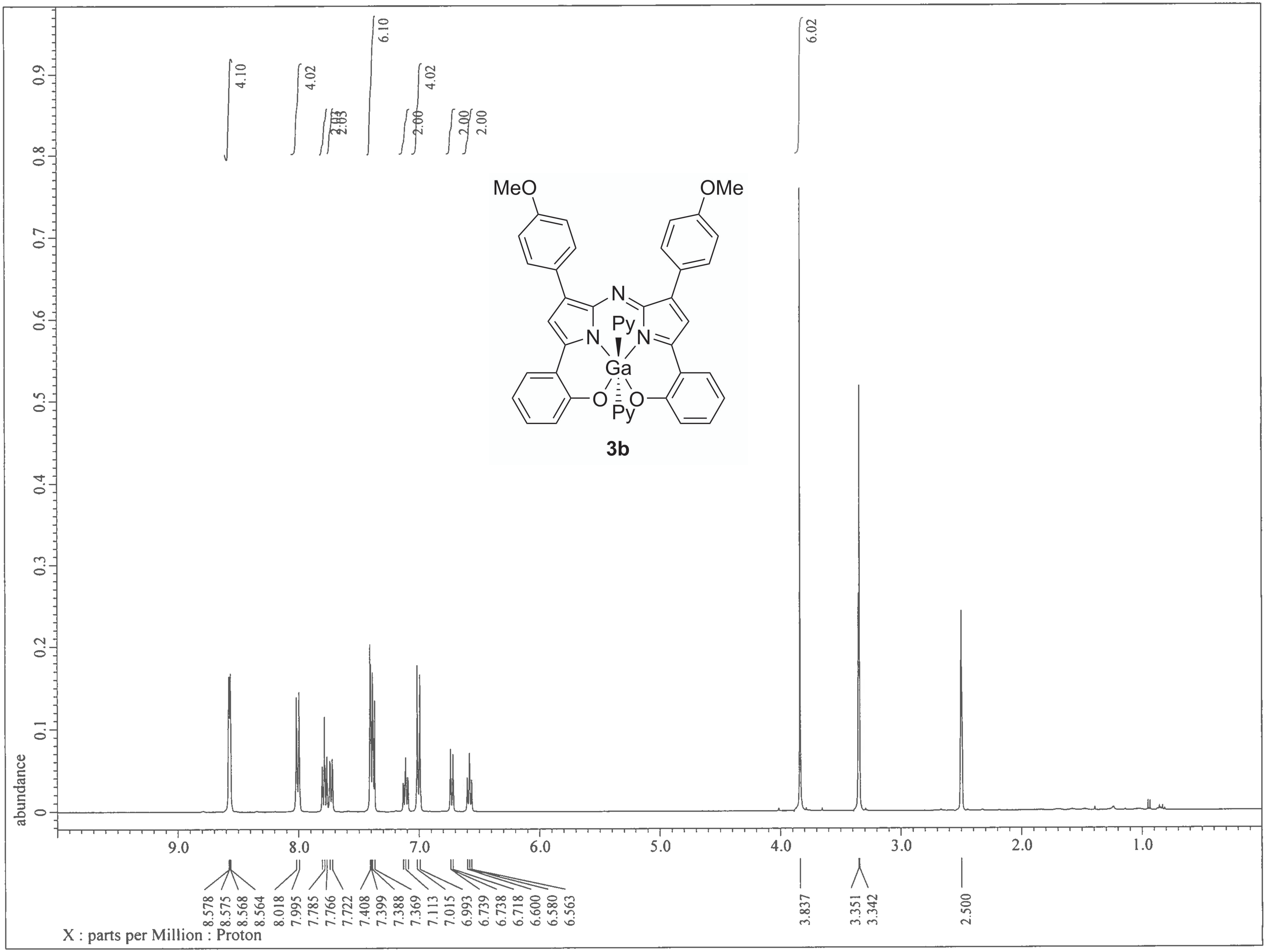

MeO
OMe
N
Py
N
N
Ga
O
O
Py
3b
4.10
4.02
6.10
2.00
4.02
2.00
2.00
6.02
abundance
0
0.1
0.2
0.3
0.4
0.5
0.6
0.7
0.8
0.9
9.0
8.0
7.0
6.0
5.0
4.0
3.0
2.0
1.0
8.578
8.575
8.568
8.564
8.018
7.995
7.785
7.766
7.722
7.408
7.399
7.388
7.369
7.113
7.015
6.993
6.739
6.738
6.718
6.600
6.580
6.563
3.837
3.351
3.342
2.500
X : parts per Million : Proton

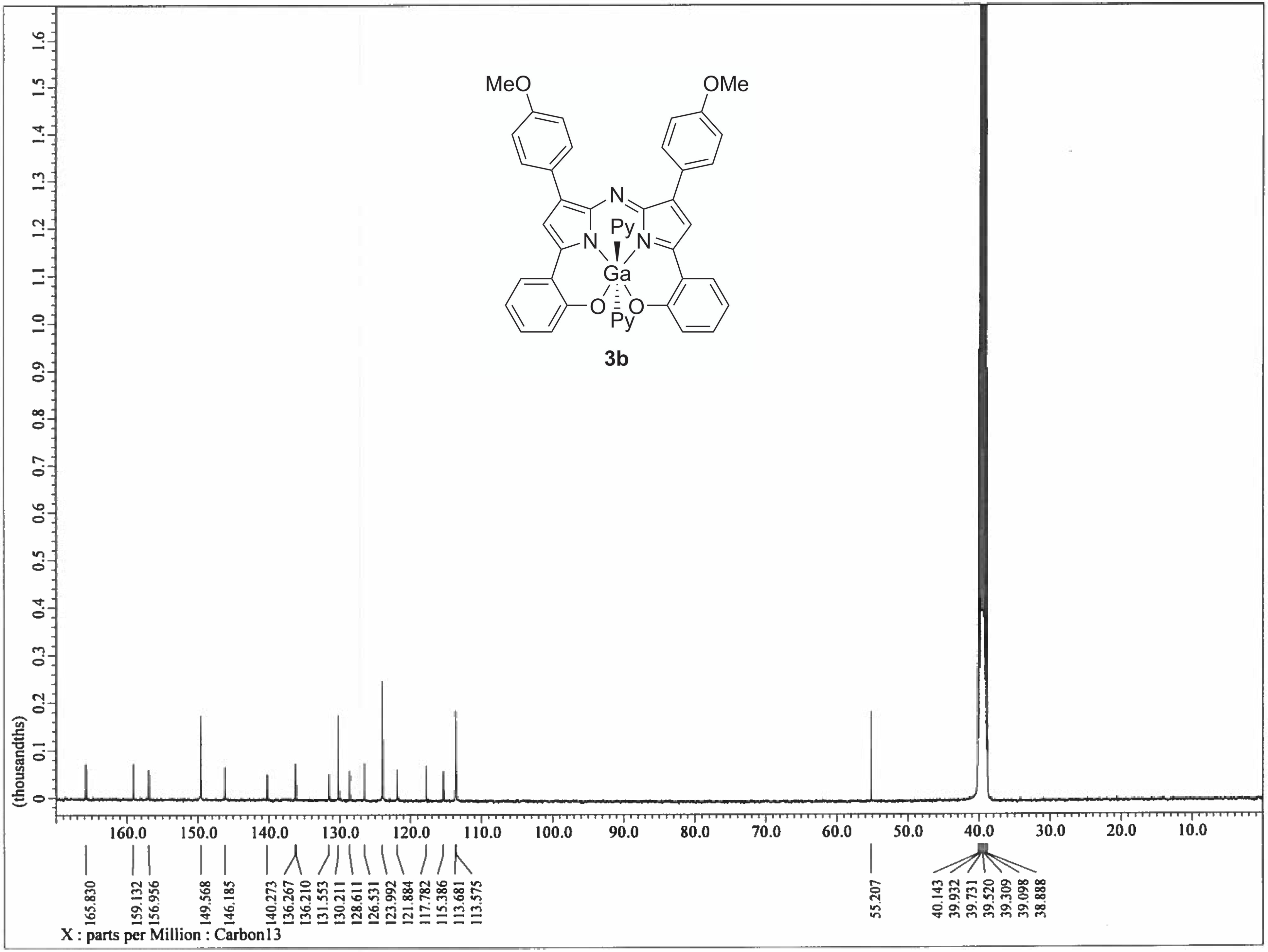
MeO
OMe
N
Py
N
N
Ga
O
O
Py
3b
(thousandths)
0
0.1
0.2
0.3
0.4
0.5
0.6
0.7
0.8
0.9
1.0
1.1
1.2
1.3
1.4
1.5
1.6
160.0
150.0
140.0
130.0
120.0
110.0
100.0
90.0
80.0
70.0
60.0
50.0
40.0
30.0
20.0
10.0
165.830
159.132
156.956
149.568
146.185
140.273
136.267
136.210
131.553
130.211
128.611
126.531
123.992
121.884
117.782
115.386
113.681
113.575
55.207
40.143
39.932
39.731
39.520
39.309
39.098
38.888
X : parts per Million : Carbon13

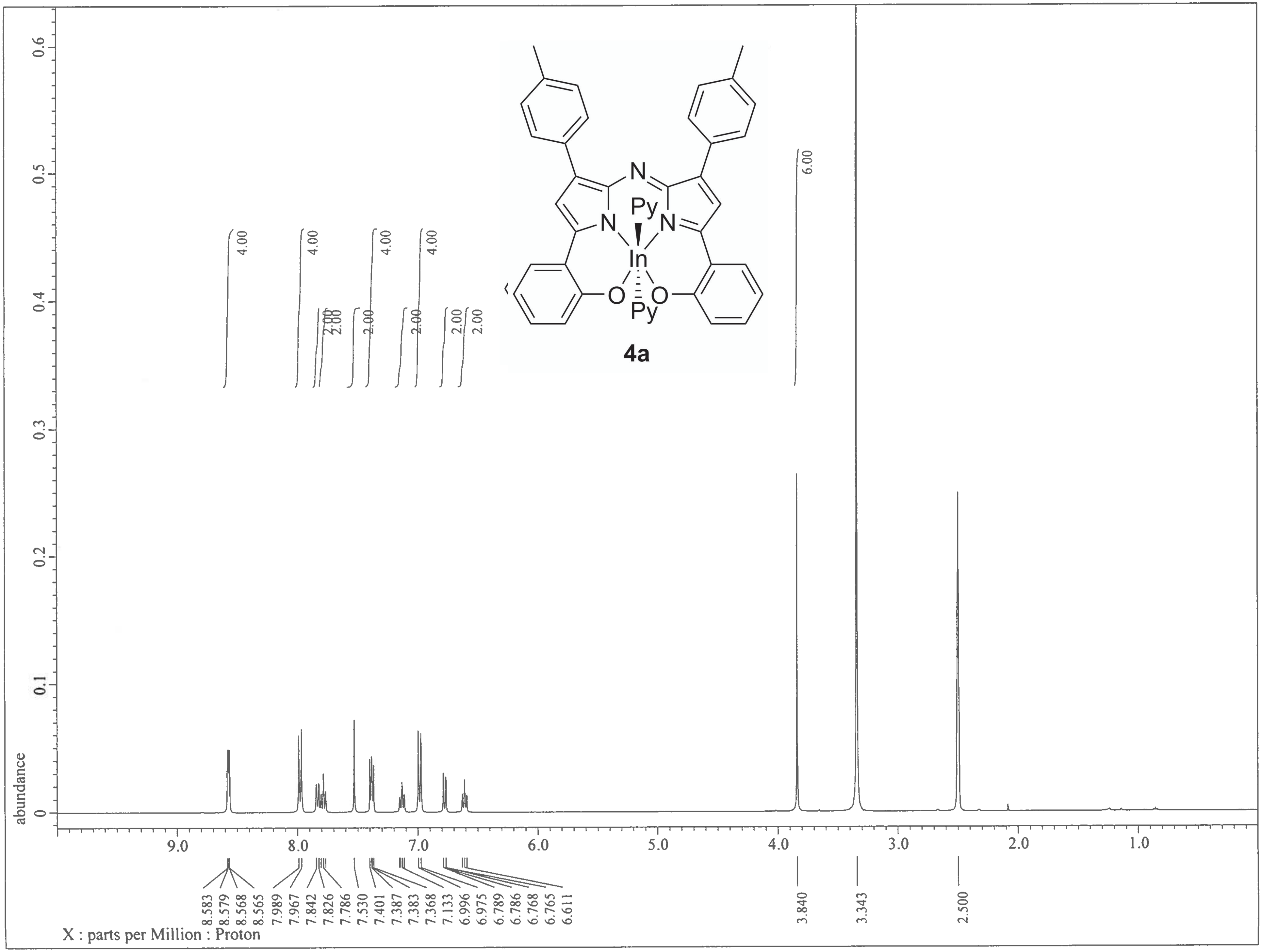

4a
N
Py
In
O
abundance
0.6
0.5
0.4
0.3
0.2
0.1
0
4.00
4.00
2.00
2.00
2.00
4.00
2.00
4.00
2.00
2.00
6.00
9.0
8.0
7.0
6.0
5.0
4.0
3.0
2.0
1.0
8.583
8.579
8.568
8.565
7.989
7.967
7.842
7.826
7.786
7.530
7.401
7.387
7.383
7.368
7.133
6.996
6.975
6.789
6.786
6.768
6.765
6.611
3.840
3.343
2.500
X : parts per Million : Proton

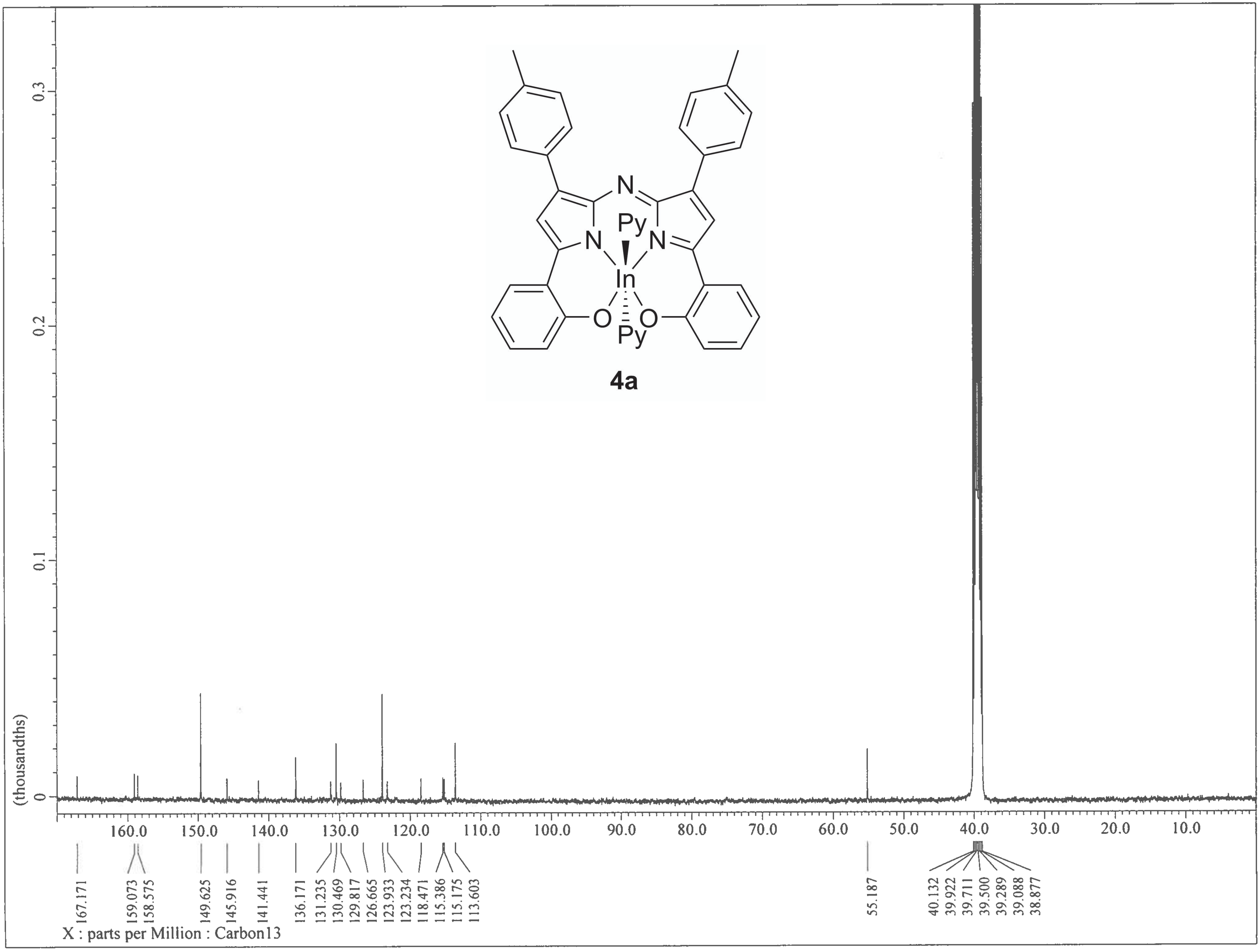
N
Py
N
N
In
O
O
Py
4a
0.3
0.2
0.1
0
(thousandths)
160.0
150.0
140.0
130.0
120.0
110.0
100.0
90.0
80.0
70.0
60.0
50.0
40.0
30.0
20.0
10.0
167.171
159.073
158.575
149.625
145.916
141.441
136.171
131.235
130.469
129.817
126.665
123.933
123.234
118.471
115.386
115.175
113.603
55.187
40.132
39.922
39.711
39.500
39.289
39.088
38.877
X : parts per Million : Carbon13

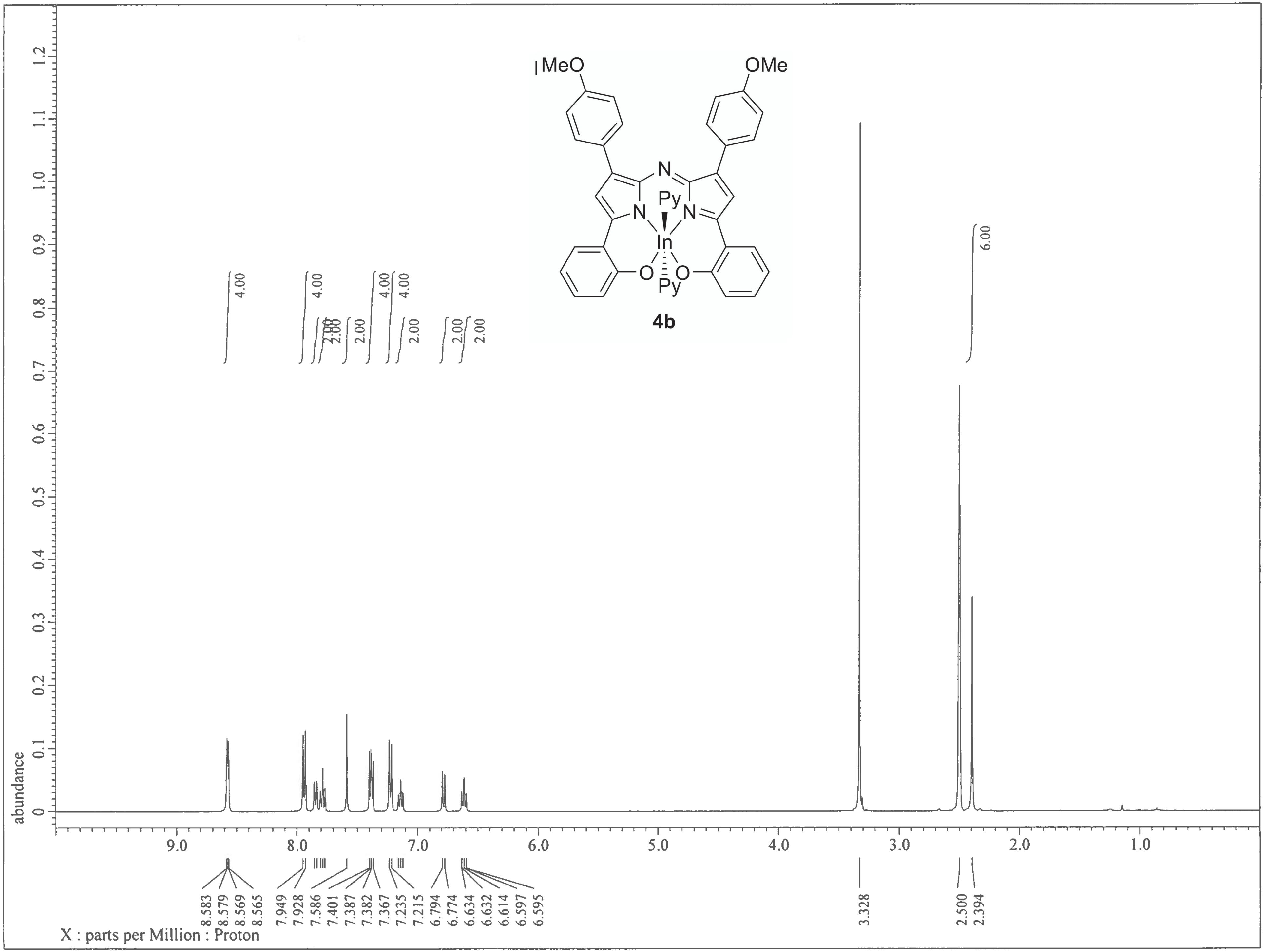
MeO
OMe
N
Py
N
N
In
O
O
Py
4b
4.00
4.00
2.00
2.00
2.00
4.00
4.00
2.00
2.00
2.00
6.00
abundance
0
0.1
0.2
0.3
0.4
0.5
0.6
0.7
0.8
0.9
1.0
1.1
1.2
9.0
8.0
7.0
6.0
5.0
4.0
3.0
2.0
1.0
8.583
8.579
8.569
8.565
7.949
7.928
7.586
7.401
7.387
7.382
7.367
7.235
7.215
6.794
6.774
6.634
6.632
6.614
6.597
6.595
3.328
2.500
2.394
X : parts per Million : Proton

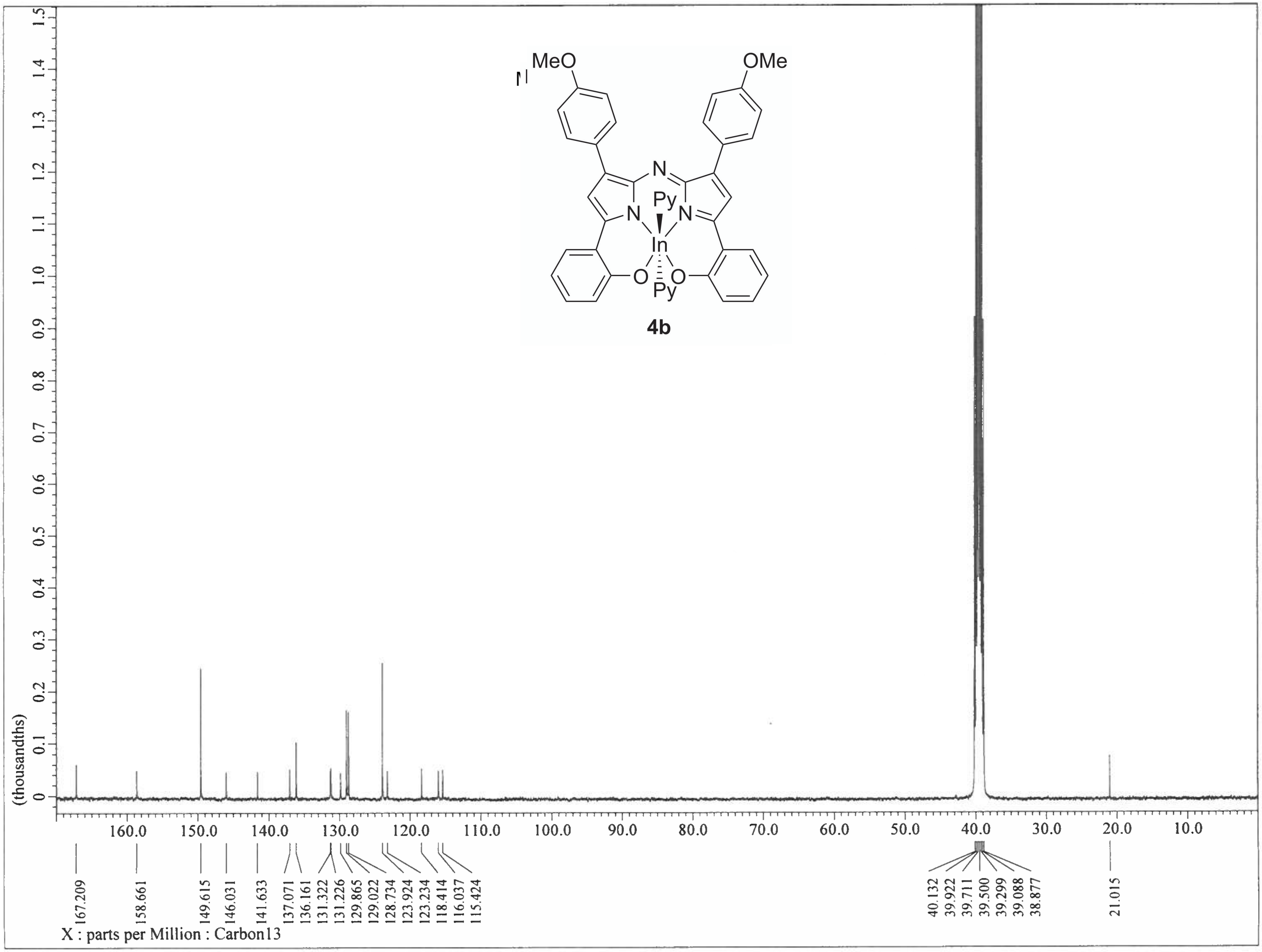
MeO
OMe
N
Py
N
N
In
O
O
Py
4b
(thousandths)
0
0.1
0.2
0.3
0.4
0.5
0.6
0.7
0.8
0.9
1.0
1.1
1.2
1.3
1.4
1.5
160.0
150.0
140.0
130.0
120.0
110.0
100.0
90.0
80.0
70.0
60.0
50.0
40.0
30.0
20.0
10.0
167.209
158.661
149.615
146.031
141.633
137.071
136.161
131.322
131.226
129.865
129.022
128.734
123.924
123.234
118.414
116.037
115.424
40.132
39.922
39.711
39.500
39.299
39.088
38.877
21.015
X : parts per Million : Carbon13
